# Usability of Electronic Health Record–Generated Discharge Summaries: Heuristic Evaluation

**DOI:** 10.2196/25657

**Published:** 2021-04-15

**Authors:** Patrice D Tremoulet, Priyanka D Shah, Alisha A Acosta, Christian W Grant, Jon T Kurtz, Peter Mounas, Michael Kirchhoff, Elizabeth Wade

**Affiliations:** 1 Department of Psychology Rowan University Glassboro, NJ United States; 2 Device Evaluation ECRI Plymouth Meeting, PA United States; 3 Department of Biochemistry Rowan University Glassboro, NJ United States; 4 Department of Computer Science Rowan University Glassboro, NJ United States; 5 Department of Biological Sciences Rowan University Glassboro, NJ United States; 6 Department of Emergency Medicine Cooper Medical School Rowan University Camden, NJ United States; 7 Concord Hospital Concord, NH United States

**Keywords:** discharge summary, usability, electronic health record (EHR), care coordination, elderly patients, patient safety, heuristic evaluation, human factors

## Abstract

**Background:**

Obtaining accurate clinical information about recent acute care visits is extremely important for outpatient providers. However, documents used to communicate this information are often difficult to use. This puts patients at risk of adverse events. Elderly patients who are seen by more providers and have more care transitions are especially vulnerable.

**Objective:**

This study aimed to (1) identify the information about elderly patients’ recent acute care visits needed to coordinate their care, (2) use this information to assess discharge summaries, and (3) provide recommendations to help improve the quality of electronic health record (EHR)–generated discharge summaries, thereby increasing patient safety.

**Methods:**

A literature review, clinician interviews, and a survey of outpatient providers were used to identify and categorize data needed to coordinate care for recently discharged elderly patients. Based upon those data, 2 guidelines for creating useful discharge summaries were created. The new guidelines, along with 17 previously developed medical documentation usability heuristics, were applied to assess 4 simulated elderly patient discharge summaries.

**Results:**

The initial research effort yielded a list of 29 items that should always be included in elderly patient discharge summaries and a list of 7 “helpful, but not always necessary” items. Evaluation of 4 deidentified elderly patient discharge summaries revealed that none of the documents contained all 36 necessary items; between 14 and 18 were missing. The documents each had several other issues, and they differed significantly in organization, layout, and formatting.

**Conclusions:**

Variations in content and structure of discharge summaries in the United States make them unnecessarily difficult to use. Standardization would benefit both patients, by lowering the risk of care transition–related adverse events, and outpatient providers, by helping reduce frustration that can contribute to burnout. In the short term, acute care providers can help improve the quality of their discharge summaries by working with EHR vendors to follow recommendations based upon this study. Meanwhile, additional human factors work should determine the most effective way to organize and present information in discharge summaries, to facilitate effective standardization.

## Introduction

Rising rates of burnout among outpatient clinicians have been linked to the adoption of electronic health records (EHRs) [1,2], which are known to have poor usability [3,4]. Other work has shown that updating EHRs is a burden for many outpatient providers [5,6]. However, few researchers have explored how inpatient providers’ adoption of EHRs impacted outpatient clinicians’ ability to assimilate information about recently discharged patients [7,8].

Most acute care facilities in the United States currently use EHRs to generate documents intended to communicate important information about patients and their recent acute care visits to outpatient providers, including skilled nursing facilities (SNFs), and long-term care facilities. These may be called transition of care documents, clinical handover documents, end-of-visit summaries, or, most commonly, discharge summaries (DSs). Since each acute care organization’s EHR system is “customized” during implementation, outpatient providers who treat patients that utilize different acute care facilities must extract information from DSs that can vary greatly in content, format, and organization. Moreover, EHR customization is performed by information technology professionals who work for EHR vendors or inpatient providers. These professionals do not possess a complete understanding of the data needs, priorities, and information presentation preferences of outpatient providers. Not surprisingly, acute care and outpatient providers have different impressions about how useful EHR-generated DSs are [9].

DSs that either make it difficult for outpatient providers to find, or do not include, all of the information needed to coordinate care put patients at risk of adverse events [10]. Elderly patients (65 years old or older) are especially vulnerable: They tend to have more comorbidities and thus are often followed by multiple outpatient specialists, and they are more likely to have multiple postacute care transitions than younger, less complex patients. One study found discrepancies between medication lists from a referring hospital and a home health care agency for *all* 770 elderly patient participants [11], putting them at risk of medication errors. Other care transition–related adverse events include treatment delays and unnecessary tests [12-14]. However, a literature search yielded only 1 study, conducted in Canada nearly a decade ago, that suggests creating specialized DSs for elderly patients [15].

A standard method of presenting information in US discharge summaries could boost patient safety by (1) facilitating information transfer, by making it easier for outpatient clinicians to find the data needed to coordinate care for their patients and (2) reducing risk of burnout-related adverse events, by decreasing frustration associated with trying to extract information from poorly organized documents [16]. A first step towards standardizing all US DSs is identifying the data that US outpatient providers need to coordinate care for recently discharged patients who are transitioning to SNFs. We focused on this scenario because we judged that medically complex elderly patients would be more likely to transition from acute care to a SNF setting than to other outpatient care settings. Moreover, the data needed to coordinate care for adults transitioning to SNFs should be a superset of the data needed to coordinate care for other elderly patients and younger adults.

This study addresses 2 gaps in knowledge: (1) Through qualitative methods, it *identifies the information that clinicians in US SNFs need to coordinate care for elderly patients* who were recently discharged from acute care and (2) through a well-established human factors technique, called heuristic evaluation, it *provides insight into how well US acute care providers’ DSs currently support outpatient providers* who coordinate care for elderly patients.

## Methods

The study included 2 phases. First, the data requirements of outpatient providers who coordinate care for elderly patients were identified through an exploratory, descriptive effort, and the knowledge gleaned from that effort was applied to specify 2 new guidelines for creating useful DSs. Second, the new guidelines were combined with 17 previously developed medical documentation usability heuristics [7] (see Multimedia Appendix 1) and applied to assess how well 4 examples of elderly patient discharge summaries support care coordination. The second part not only shed light on how difficult it can be for outpatient providers to use the discharge summaries currently being produced by inpatient providers’ EHRs but also yielded several recommendations for improving the quality of EHR-generated discharge summaries. All research processes and procedures for both parts of this study were approved by Rowan University’s Institutional Review Board, and exemptions were granted by the institutional review boards of the 2 hospitals that provided deidentified examples of discharge summaries.

### Development of DS Content Guidelines

A literature review served as the first step towards identifying the data that are necessary or helpful when coordinating care for a recently discharged elderly patient. Aggregating the items identified at the Transitions of Care Consensus Conference [17], which includes 6 items that the Joint Commission mandates be included in all DSs [18], with the items in the standardized DSs used in Australia [19] and the items recommended in a Canadian study that focused specifically upon creating DSs for elderly patients [15], yielded a list of 26 items. See Multimedia Appendix 2 for items recommended by different sources. Next, 15 outpatient care providers who frequently care for elderly patients were interviewed. These care providers included primary care physicians, nurse practitioners, directors of nursing, social workers, transition-of-care nurses, and medical directors. They were asked to categorize each of those 26 items as “always necessary,” “helpful, but not required,” or “not relevant/distracting” and then invited to name any additional data that they recommend be included in elderly patient DSs. After the 15 interviews, the list had grown to 36 items. See Multimedia Appendix 3 to view the structured interview questions. Those items were included in an online survey sent to 2500 members of a Continuing Care Risk Management community that asked participants to categorize the 36 items using the same 3 categories. See Multimedia Appendix 4 to view the survey questions. A majority of the 58 respondents indicated that all 36 items were helpful and that 29 of the 36 should always be included in elderly patient DSs. Thus, 2 content guidelines were established: One states that each of 29 items should be included in DSs, and the second recommends that the remaining 7 items be considered for inclusion (see Results section).

### Creation of Simulated DSs

**Figure 1:**
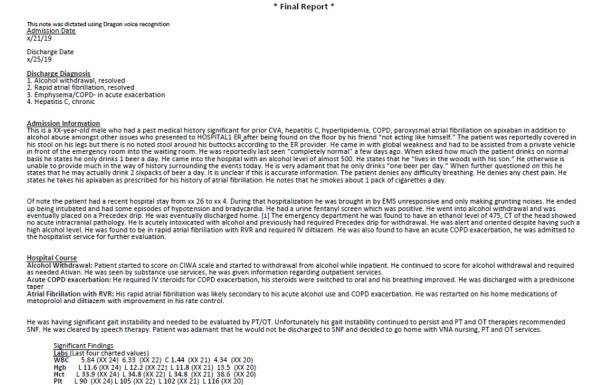
The top portion of a simulated discharge summary from hospital 1.

**Figure 2:**
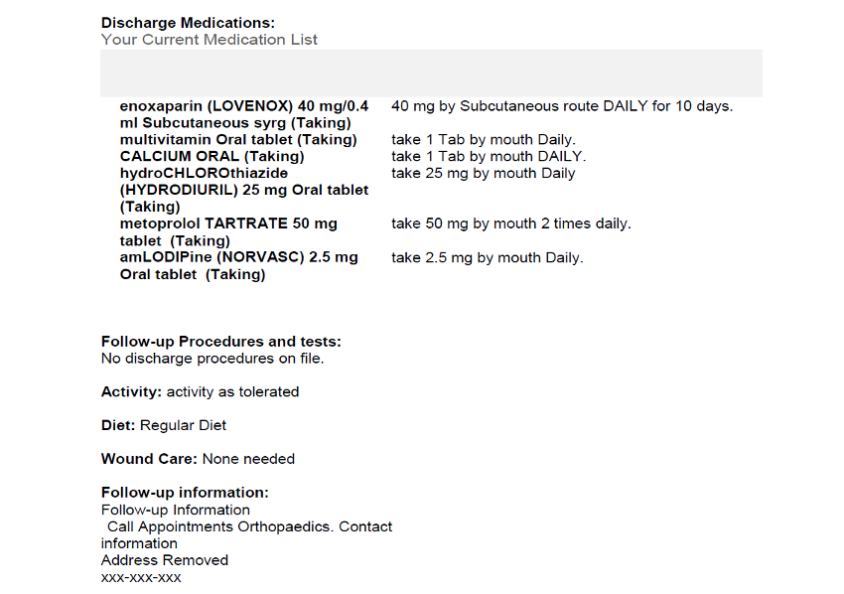
The middle portion of a simulated discharge summary from hospital 2.

Two hospitals, which use systems from different EHR vendors, each provided 10 deidentified elderly patient DSs that were produced by their EHR system. Two of the DSs from each hospital were randomly selected. Simulations of those 4 DSs, which appeared the same as the original documents (eg, same font size and style, layout, headings), were created to keep heuristic evaluation participants blind to the hospitals. The safe harbor method was applied in accordance with the Health Insurance Portability and Accountability Act of 1996 Privacy Rule. This entailed replacing not only all protected health information about the patient (which had already been deidentified) but also all doctors’ names and all health care organization names with fictitious data. The 4 simulated DSs, which are referred to as H1P1 (Hospital 1, Patient 1), H1P2 (Hospital 1, Patient 2), H2P1 (Hospital 2, Patient 1), and H2P2 (Hospital 2, Patient 2), were then reviewed for validity, including faithful replication of formatting, layout, and organization. Figures 1 and 2 show portions of 2 of the simulated DSs. See Multimedia Appendices 5-8 to view the full simulated DSs.

### Heuristic Evaluation

Heuristic evaluation is a usability assessment technique in which 3-5 trained experts independently apply a set of design best practices, called heuristics, to identify potential usability problems [20]. The participants also rate the severity of the issues, and then all issues and ratings are analyzed collectively [21]. Participants are not expected to discover the same issues, but together they generally identify the most important usability problems [22]. Proponents of heuristic evaluation recommend developing a set of relevant heuristics for the particular type of item being assessed, which can serve as guidance for developing useable products as well as tools for assessing usability [20].

In this study, 5 human factors experts independently assessed each of the 4 simulated discharge summaries. Next, 5 clinical experts experienced in providing outpatient care each independently rated the severity of all of the potential issues identified by the human factors team. Clinical experts were also given the opportunity to report any additional issues they found in the simulated discharge summaries. This iterative approach to heuristic evaluation, which reduces the time required by clinical experts, has been used successfully to evaluate medical technology [7,23]. This approach is also consistent with Nielsen’s recommendation that experts first be asked to use heuristics to identify issues and later be asked to rate the severity of all issues [24].

The human factors team was given a set of 19 assessment tools: 17 previously developed medical document usability heuristics [7] (see Multimedia Appendix 1) and the 2 new discharge summary content guidelines (see Results section) and instructions on how to apply the heuristics to assess discharge summaries. The 5 human factors team members independently identified potential usability issues by looking for violations of the heuristics or the guidelines. Once each team member had evaluated each document, the issues were aggregated into 4 lists (one per simulated discharge document). Then, the items in the lists were paraphrased, and duplicates were removed.

**Figure 3:**
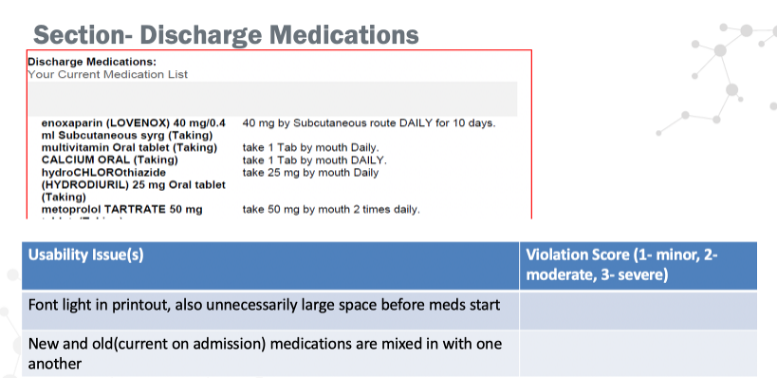
Screenshot of a slide used to summarize potential issues found by the human factors team.

Each of the 4 issue lists was ordered and grouped based upon the way information was presented in the simulated discharge summaries and then described in a set of slides (see Figure 3). Next, 5 clinical experts independently reviewed the simulated discharge documents, provided severity ratings for each issue, and reported and rated any additional usability issues they identified. The clinical experts were provided with a 4-step severity scale. We adapted Sauro’s 3-point scale [25] by adding a level 0 for “not an issue” and then replacing the term “critical” with the word “severe” for level 3. The latter change was intended to prevent participants from avoiding use of that rating; in prior work, some clinicians were reluctant to use “critical,” reserving that for issues that indisputably cause harm [7]. Table 1 describes the levels and provides an example of an issue at each level.

**Table 1:** Descriptions of the 4 levels used to rate the severity of issues found in simulated discharge summaries and examples of issues at each level.

Rating	Description	Example issue
0	Not a problem	Note stating that contents were produced using Dragon Dictate not needed
1	Minor: hesitation or slight irritation	Redundant information found in hospital course
2	Moderate: causes delays and moderate irritation	Nutritional status on discharge and Do not resuscitate orders “hidden” in relatively lengthy hospital course section
3	Severe: causes frustration or potential for error; must be fixed	No indication of whether medication is temporary or permanent

In some cases, the evaluators suggested specific solutions to issues (eg, restrict use of all CAPS, never abbreviate medication names, include page numbers in the format “page X of Y”). All solutions were summarized and grouped so that common themes could be extracted. These themes were transformed into recommendations (see Discussion section).

## Results

The knowledge assembled during the literature review, interviews, and survey was applied to develop 2 guidelines for creating useful, easy-to-use discharge summaries. These guidelines are shown in Tables 2 and 3.

**Table 2:** First new guideline, with the list of items that should be included in elderly patient discharge summaries and how many of the 4 simulated discharge summaries were missing each item.

Guideline 1. Include each of the following in elderly patient discharge summaries	Number of documents missing this item (n=4)
Patient identifiers (eg, LAST name, first name, middle name, date of birth, age, gender, medical record number)	2
Date of admission/discharge	0
Hospital admission diagnosis	0
Principal/primary diagnosis (responsible for the largest portion of the patient's stay)	3
List of discharge diagnosis	0
Discharge medications (when patient is discharged from acute care)	0
History of present illness for hospitalization	2
Hospital course (events occurring during patient’s hospital stay)	0
Procedures performed in hospital	0
Laboratory tests and investigation results (including pending results & tests due)	3
Patient physical and cognitive functional ability at discharge	3
Discharge status/Patient's discharge condition (how the patient is doing, relevant physical findings, patient's health status)	4
Medication on hospital admission	4
(Reasons for) Changes in medication during patient's stay in hospital	4
Adverse reactions during stay (including allergies to medications and other allergies)	4
Discharge instructions	2
Appointments after discharge	2
Life-sustaining treatments preferences (DNR^a^, lifesaving instructions, POLST^b^)	3
Nutritional status at discharge from hospital	3
Immunization history	4
Patient demographics (eg, address, phone number)	4
Follow-up issues	1
Emergency contact information	4
Nutritional status at hospital admission	4
Patient’s physical and cognitive functional ability at admission	3
Goals of care and treatment plan during hospital stay	4
Discharging physician contact information	4
Contact information of clinician(s) who consulted patient in hospital	4
Patient weight	3

^a^DNR: do not resuscitate.

^b^POLST: portable medical orders.

**Table 3:** Second new guideline, with the list of items that should be considered to be included in elderly patient discharge summaries and how many of the 4 simulated discharge summaries were missing each item.

Guideline 2. Consider including the following items in elderly patient discharge summaries	Number of simulated documents missing this item (n=4)
Family history	4
Social and lifestyle history	4
Free-text comments (a field for clinicians to share miscellaneous notes about the patient)	3
Type of medical devices or equipment (eg, bariatric beds) that are needed for the patient in the SNF^a^	4
Goals of care and treatment plan post hospital discharge	4
Activities of daily living (ADL) status	4
Wound, skin, fall assessment	1

^a^SNF: skilled nursing facility.

When these 2 guidelines were combined with 17 previously developed medical document usability items (shown in Multimedia Appendix 1) and applied to assess the 4 simulated discharge documents, the human factors team identified 98 issues. See Multimedia Appendices 9-12 for the Powerpoint slides used to present these 98 issues to clinical experts. Clinical experts identified 19 additional issues. The average severity rating across all issues and all documents was 1.69, between minor and moderate. The total number of issues and average severity ratings for each of the simulated DSs were as follows: H1P1: 40, 1.91; H1P2: 30, 1.67; H2P1: 21, 1.73; H2P2: 26, 1.36. The entire set of issues was combined and then grouped based upon how they impact the 5 categories of document usability identified in a previous study [7]: readability, comprehensibility, minimalism, content, and organization. The counts and average severity ratings of the issues associated with each of these categories were as follows: comprehensibility: 8, 2.00; content: 42, 1.99; organization: 31, 1.77; readability: 36, 1.27; minimalism: 0. Finally, Table 4 lists all issues with average severity ratings greater than or equal to 2.

**Table 4:** Usability issues with average severity ratings ≥2.

Issue	Average severity rating
Postoperative patient with no wound care instructions	3
Treatable condition listed in discharge diagnoses, but patient status, plan, and medication missing	3
No indication whether medications are temporary or permanent^a^	3
Formatting makes medication section difficult to read or understand^a^	3
Medications need to be prioritized^a^	3
Medication frequency missing^a^	3
Length of time on IV^b^ meds is missing^a^	3
No diagnosis linked to the medications^a^	3
Medication is missing information on “PRN” (when is it needed?) ^a^	3
New and “old” medications are mixed in with one another^a^	2.67
Electronic signature gives name but no phone or email	2.67
Indenting gives impression that patient discharge condition and time spent on discharge are subordinate to physical exam	2.5
Nutritional status and Do Not Resuscitate orders “hidden” in Hospital Course section	2.33
No page numbers	2.13
Follow-up appointments listed in Hospital Course section	2.08
Heading with no content	2
Formatting made significant findings section hard to read	2

^a^Related to the section(s) listing medications.

^b^IV: intravenous.

Each of the clinical experts indicated that the simulated discharge summaries were representative of discharge summaries that they had seen before. They further characterized the examples as “fairly good” in quality when asked to compare them to those that they typically encounter. However, they agreed that each had significant room for improvement. They also agreed that it would be beneficial if DSs from all acute care providers could be standardized.

## Discussion

### Principal Findings

The knowledge developed through this effort may be broadly applied by acute care organizations seeking to assess or improve the usability of their discharge documents. More specifically, acute care providers can work with EHR vendors to apply the 2 guidelines introduced here to help ensure that they produce discharge summaries that contain the information that outpatient providers need to coordinate care for elderly patients. In addition, acute care providers should consider implementing the recommendations listed in the following sections, which are based upon the results of assessing 4 simulated discharge summaries that were based on real documents. These recommendations have been divided into 2 groups: The first contains those that can be implemented in software, and the second contains those that must be fulfilled by humans — though EHR software could definitely provide prompts or help verify these recommendations have been followed. See Multimedia Appendix 13 to review how several of the issues identified by clinical experts are associated with particular recommendations.

### Recommendations

Textbox 1 and Textbox 2 include the recommendations for adapting EHRs so they produce more useable discharge summaries.

**Recommendations for adapting electronic health records (EHRs).**

1. Require users to make sure that medication information is complete.
  - For each medication, require and display the following: medication name; medication strength, dose, dosage unit, route of administration, and frequency; indication for the medication (which diagnosis or complaint is targeted by this medication; if PRN, ensure that the “as needed” criteria are defined within the medication section of the discharge summary); start date; end or refill date; indicator of whether the medication is temporary (end at completion of course) or permanent or chronic (will need refills).
  - If any information (dose, frequency, end or refill date) is missing, prompt users to insert missing information.
2. Present all medication information clearly and consistently.
  - Apply formatting and layout to draw attention to needed information (eg, bold the medication name, separate information with spaces or dashes).
  - Do not use abbreviations.
  - List medications by generic name only, not a mix of generic and brand names.
  - Differentiate new start versus continued medications versus medications that should be stopped.
3. Provide a section for durable medical equipment, so it can be separated from medications.
4. Require and display contact information so outpatient providers can follow-up with an inpatient provider.
  - Prompt for an email address or phone number if only a name is given.
5. Display start and end dates for all procedures.
  - Prompt for dates if any are missing.
6. Print page numbers, in the form “xx of yy pages” when documents are printed, and display page numbers when viewed online to facilitate conversations where one person is viewing a print out and one is viewing online.
7. Use colors or shading that provide sufficient contrast if documents are printed in black and white.
8. Show all headings, even if there is no content in a section.
  - When no accompanying text is provided, prompt for input or obtain user’s approval to populate it with “—“ so readers can verify that the section has been intentionally left blank.
9. Don't allow page breaks between section headings and section content.
10. Apply consistent font style, font sizing, spacing, layout, indentation, and heading style.
  - Font size of the text must be at least 12 points to be easily readable. In some cases (eg, older audiences), selective use of a larger font (14 points) may be advisable, since it can help readers more easily see and focus attention on the most important information. Since the font size depends on the font type selected, maintain a size of 16 pixels at minimum [26].
  - Maintain a line height that is 130% to 150% larger than the font size [27].
  - Ensure that the section headings and subheadings stand out. Consider increasing the font size, or bold the heading text.

**Recommendations that require user action (but that software can prompt or try to verify).**

1. Provide an explanation of patient’s condition that includes at least a grade (eg, poor, good) and add a justification for any deviation from good.
2. Do not abbreviate medication or procedure names (eg, AMOX for amoxicillin) to ensure absolute clarity of the conveyed information.
3. Avoid using “all caps,” which makes the content hard to read.
4. Emphasize important information. Ensure that the date of exam(s) or lab test(s) is prominently mentioned.
5. Ensure that all procedures undergone during an acute visit are listed. Examples include the use of feeding tubes, dietary restrictions, total parenteral nutrition (TPN), or urinary catheter.
6. Ensure that the content matches the headings and subheadings within each section.

Acute care providers may also consider working with EHR vendors to redesign after visit summaries (AVSs) based upon the these recommendations, since prior research has revealed that AVSs are frequently used to develop care plans [7]. Using an AVS, which is the document that is presented to a patient upon discharge from acute care, as a surrogate for a DS is not optimal practice. However, given the current practical limitations in EHR system provider-to-provider communication, it is in patients’ best interests for acute care providers to adapt their AVSs so they contain the information that outpatient physicians need to coordinate care for recently discharged patients.

### Limitations

While this work represents a necessary starting point for eventually standardizing adult patient discharge summaries, it has several limitations. Since the only incentive offered to survey recipients was access to survey results, only 58 out of 2500 (2.32%) of them responded, even after sending multiple reminders. Accordingly, the lists of important “discharge summary components” identified in this study need to be verified by a larger number of outpatient providers. Furthermore, a larger sample of discharge summaries or other usability testing methods may have revealed more issues, leading to additional recommendations. Finally, given the complexity and high level of customization of hospital EHR systems, it is currently unclear how difficult it would be for hospitals to configure their systems to output documents that follow our recommendations or to support users in following them. For example, some current systems are designed to group medication list items into Stop, Start, and Continue categories, which could make it hard to separate durable medical equipment from medications. On the other hand, it would make sense for inpatient providers to request that EHR vendors use the recommendations from this study to make changes to their products that could then be pushed out as software upgrades to *all* inpatient providers.

### Comparison With Prior Work

A heuristic evaluation of 4 AVSs generated by acute care providers’ EHR systems revealed formatting and organizational issues that made those documents very difficult to use [7]. The DSs evaluated in this study had fewer formatting and organizational issues, and those issues were rated lower in terms of severity than the ones found in the AVSs. The DSs in this study also had much less irrelevant or unhelpful text than the AVSs in the prior study. On the other hand, there were many more issues related to missing or unclear content in the DSs, and those issues were generally considered to be fairly severe (rated 2-3). This is not surprising because the content guidelines developed for this effort had not been established when the AVSs were evaluated, so their content was only assessed superficially, using the generic heuristics found in Multimedia Appendix 1.

Australia and the United Kingdom have developed national standards for electronic DSs [18,28], and Canadian researchers have explored standardizing DSs in Nova Scotia [29]. In contrast, most US research aimed at increasing patient safety during care transitions focuses upon improving discharge planning processes (eg, training clinicians to generate more useful discharge documentation [30,31] or involving patients and caregivers in discharge planning [31,32]).

A few US organizations have developed templates, outlines, or checklists to standardize their own DSs [33-35], but each of them organizes patient information differently. Moreover, even though a 2009 Transitions of Care Consensus Conference produced a list of items that participants recommended be included in transition records [17], recent research indicates that EHR-generated discharge summaries in the United States continue to omit data that outpatient providers need to effectively coordinate care [27,36-39].

### Conclusions

In summary, this project addresses 2 current gaps in knowledge among inpatient providers and EHR vendors: (1) What data do outpatient providers need to coordinate care for elderly patients recently discharged from acute care facilities to an outpatient facility? and (2) How well do the EHR-generated DSs currently being produced by acute care providers meet outpatient providers’ needs? This work revealed not only that documents currently being produced do not fully meet outpatient provider needs but also that 2 DSs produced by the same acute care provider organization can vary significantly in layout, organization, structure, and content. The current heterogeneity among DSs makes it unnecessarily difficult for outpatient providers to coordinate care for recently discharged patients. This puts patients at risk of adverse events and may contribute to outpatient provider burnout. This work is especially timely given that the Office of the National Coordinator for Health Information Technology and the Centers for Medicare and Medicaid Services recently released proposed rules to facilitate “seamless and secure” electronic transfer of patient data [40]. Seamlessly and securely sharing patient information is not sufficient to ensure high-quality care coordination. Patient data must be delivered in a form that enables clinicians to quickly and easily locate and understand the information most relevant to them. In short, there is an urgent need for additional applied human factors research focused upon improving the quality of the clinical documentation produced by EHR systems. This research should be conducted in parallel with ongoing interoperability efforts, so that once it is possible for EHRs to seamlessly transfer patient data, they will be shared in a concise, well-organized, easy-to-understand form.

## Appendix

Multimedia Appendix 1 Medical Document Usability Heuristics adapted from Tremoulet et al, 2018.

Multimedia Appendix 2 Discharge Summary components recommended by different sources.

Multimedia Appendix 3 Outpatient Provider Structured Interview Questions.

Multimedia Appendix 4 Survey used to help develop two new discharge summary heuristics.

Multimedia Appendix 5 Simulated Discharge Summary for patient 1 from hospital 1.

Multimedia Appendix 6 Simulated Discharge Summary from patient 2 from hospital 1.

Multimedia Appendix 7 Simulated Discharge Summary for patient 1 from hospital 2.

Multimedia Appendix 8 Simulated Discharge Summary for patient 2 from hospital 2.

Multimedia Appendix 9 Potential usability issues in simulated discharge summary for patient 1 from hospital 1.

Multimedia Appendix 10 Potential usability issues in simulated discharge summary for patient 2 from hospital 1.

Multimedia Appendix 11 Potential usability issues in simulated discharge summary for patient 1 from hospital 2.

Multimedia Appendix 12 Potential usability issues in simulated discharge summary for patient 2 from hospital 2.

Multimedia Appendix 13 Locations of several DS issues identified by clinical experts and the recommendations that are related to those issues.

## Abbreviations

AVS: after visit summary
DS: discharge summary
EHR: electronic health record
SNF: skilled nursing facility
