# Supplementary material for: Usability of Electronic Health Record–Generated Discharge Summaries: Heuristic Evaluation
Source: J Med Internet Res. 2021 Apr 15;23(4):e25657. doi: 10.2196/25657 (PMC8085750; doi:10.2196/25657)
Supplement: Multimedia Appendix 1 [file jmir_v23i4e25657_app1.docx]

**Appendix 1**

**Table A1: Medical Document Usability Heuristics adapted from (Tremoulet et al, 2018)**

| **Heuristic Category** | **Heuristic Name** | **Description** |
| --- | --- | --- |
| Readability | Color and Contrast | Text should have sufficient contrast |
|  | Layout and Position | Layout should be appealing, clear and consistent across the document |
|  | Font and Capitalization | Font style and size should be consistent and readable. |
|  | Structure/ format | Structure and format of each section should be effective and uniform |
|  |  |  |
| Minimalism | Simple and Direct | Language and sentence structure should be simple, direct, specific, concrete and concise |
|  | Progressive level of detail | Document should present the most important information first, following with increasing levels of detail |
|  |  |  |
| Comprehensibility | Terminology | Complex and technical terms must be used correctly and consistently |
|  | Clarity of Headings | Headings should be clear and understandable |
|  |  |  |
| Content | Clarity of Content | Purpose of the material should be obvious |
|  | Emphasis | Important points should be emphasized appropriately. It should be it clear why certain text is emphasized. |
|  | Context | Document should include creation or printing date and contact information |
|  | Relevance | All content should be relevant to the patient's condition and context. (There should not be extraneous information) |
|  | Absence/ Lack of information | No important content should be missing |
|  |  |  |
| Organization | Grouping | Information should be grouped in a meaningful format; Groups should be reasonably sized |
|  | Order | Information should be ordered logically |
|  | Use of Subheadings | Document should use prominent and meaningful headings and subheadings |
|  | Navigational Tools | Material should have navigational tools to help orient the reader |
