# Supplementary material for: Usability of Electronic Health Record–Generated Discharge Summaries: Heuristic Evaluation
Source: J Med Internet Res. 2021 Apr 15;23(4):e25657. doi: 10.2196/25657 (PMC8085750; doi:10.2196/25657)
Supplement: Multimedia Appendix 2 [file jmir_v23i4e25657_app2.docx]

**Appendix 2**

**Table A2: Discharge summary components recommended by the different sources which were used to develop an initial list to share with outpatient providers.**

| **Source** | **Components recommended (or mandated) for inclusion in discharge summaries** |
| --- | --- |
| Joint Commission (Kind et. al., 2008) | 1. Reason for hospitalization, 2. Significant findings, 3. Procedures and treatment provided, 4. Patient's discharge condition, 5. Patient and family instructions (as appropriate), 6. Attending physician's signature |
| Transitions of Care Consensus Conference (Snow et. al., 2009) | Six items specified by Joint commission (above) and: 7. Coordinating clinicians, 8. Care plans/transition record, 9. Principle diagnosis and problem list, 10. Medication list (reconciliation) including over the counter/herbals, allergies, and drug interactions, 11. Clearly identifies the medical home/transferring coordinating physician/institution and their contact information, 12. Patient's cognitive status, 13. Test results/pending results |
| Australian Commission on Safety and Quality in Health Care (“National Guidelines for On-Screen Presentation of Discharge Summaries”, 2019) | 1. Patient details, 2. Hospital details, 3. Recipients, 4. Author, 5. Presentation details (presentation date, discharge date, length of stay, episode type, clinical unit, senior clinician, discharge destination), 6. Problems and diagnoses, 7. Procedures, 8. Clinical summary, 8. Allergies/Adverse reactions, 9. Medications on discharge, 10. Ceased medicines, 11, Alerts, 12. Recommendations, 13. Follow-up appointments, 14. Information provided to the patient, 15. Recipients, 16. Selected investigation results |
| Institut universitaire de gériatrie de Montréal (Kergoat et al, 2010) | 1. Reason for admission, 2. Main diagnosis and other active diagnoses (specify if allergy, chronic pain, tobacco, alcohol), 3. Non-active diagnoses, 4. Social and life-style history upon admission (marital status, household arrangements, guaranteed income supplement, legal protection measures, services received, etc.), 5. Pertinent findings of the medical history-taking or the physical exam (specifically vision, audition, musculoskeletal, and neurological), 6. Investigations and consultations (labs, imaging, other) 7. Mental functions (Cognitive status, affective status, neurobehavioral symptoms associated with dementia, facultative status), 8. Functional status (activities of daily living, instrumental activities of daily living, urinary or fecal incontinence, mobility/transfer, technical support, facultative/walking speed), 9. Nutritional status, 10. Psychosocial assessment, 11. Evolution of clinical problems during hospitalization, 12. Instructions at discharge and follow-up, 13. Patient orientation, 14. Additional notes, 15. Signature of primary hospital physician, 16. Name of family physician, 17. Local community service center attended, 18. Resource-person, 19. Copy given to (patient, other) |
