# Supplementary material for: Usability of Electronic Health Record–Generated Discharge Summaries: Heuristic Evaluation
Source: J Med Internet Res. 2021 Apr 15;23(4):e25657. doi: 10.2196/25657 (PMC8085750; doi:10.2196/25657)
Supplement: Multimedia Appendix 3 [file jmir_v23i4e25657_app3.docx]

# In-person Clinician Interview Questions

1. What document(s) do you usually receive from the acute care facility: Discharge summary or a transfer form, After Visit Summary, other?
2. How do you receive discharge summaries?
   1. With the patient (paper)
   2. Fax
   3. Emailed PDF
   4. Imported to electronic health record
   5. Provider access to their credential/access portal
3. When do you receive discharge summaries?
   1. Before patient arrives
   2. As the patient arrives
   3. After plan of care was developed
   4. Other: Please specify __________________________________________
4. On average, how many recently discharged patients do you need to coordinate care for each month?
   1. less than 5
   2. 5-10
   3. greater than 10
5. How many hospitals discharge patients into your care?
   1. 1-2
   2. 3-4
   3. 5+
6. What important information do you find missing from elderly patient discharge summaries?
7. Is there any information that should be eliminated from all discharge summaries?
8. Is there anything else about discharge summaries you would like us to know?
9. Has the adoption of electronic records by inpatient providers changed your workflow? If so how?
10. When comparing EHR-generated and handwritten or dictated discharge summaries, how have the following changed?
    1. Content
    2. Quality
    3. Usability (including readability, comprehensibility, and organization)
    4. Length
11. How do you prefer the information in discharge summaries to be organized? Please use our index cards to group and order the items below. (We will create new cards if there are additional items you believe should be included in elderly patient discharge summaries.)

|  |
| --- |
| Admission diagnosis |
| Date of admission/discharge |
| Principal/Primary diagnosis (Diagnosis responsible for the largest portion of the patient's stay) |
| Patient identifiers (DOB, Name, MRN) |
| Patient demographics (Address, Phone #, Marital status etc) |
| History of present illness for hospitalization |
| Medication on admission |
| Family history |
| Social and lifestyle history |
| Physical findings relevant ~~to diagnosis~~ at Discharge |
| Hospital course (a description of the events occurring to a patient during his/her hospital stay) |
| Procedures performed in hospital |
| Laboratory tests and investigation results (including pending results) |
| Changes in medication during patient’s stay in hospital |
| Discharge status/Patient’s discharge condition (refers to how the patient is doing at discharge or the patient’s health status on discharge). |
| Patient’s: physical and cognitive functional ability at discharge |
| Patient’s: physical and cognitive functional ability at admission |
| Nutritional status at discharge |
| Nutritional status at admission |
| List of discharge diagnoses |
| Discharge instructions |
| Discharge medications |
| Follow-up issues |
| Appointments after discharge |
| Goals of care and treatment plan during hospital stay |
| Life-sustaining treatment preferences |
| Free-text comments |
| Discharging physician contact information |
| Contact information for doctors who consulted patient in hospital |
| Emergency contact information |
| Adverse reactions during medical stay |
| Immunization |
