# Supplementary material for: Usability of Electronic Health Record–Generated Discharge Summaries: Heuristic Evaluation
Source: J Med Internet Res. 2021 Apr 15;23(4):e25657. doi: 10.2196/25657 (PMC8085750; doi:10.2196/25657)
Supplement: Multimedia Appendix 4 [file jmir_v23i4e25657_app4.docx]

ECRI Institute Usability Project: EHR-Generated Discharge Documents

*1. Please specify your position

|  | Admissions Nurse |
| --- | --- |
|  | Care Coordinator/ Transition of Care Nurse |
|  | Care Nurse |
|  | Charge Nurse/ Nursing Shift Manager |
|  | Director of Nursing |
|  | Medical Director |
|  | Nurse Practitioner |
|  | Outpatient physician |
|  | Unit Nurse Manager |
|  | I prefer not to disclose |
|  | Other (please specify):  |

2. What document(s) do you usually receive from the acute care facility?

|  | After Visit Summary/ Clinical Summary/ Patient Instructions |
| --- | --- |
|  | Discharge Summary |
|  | Envelope/ Packet |
|  | Transfer Form/ Universal Transfer Form |
|  | Progress Notes |
|  | Referral Form |
|  | Other:  |

3. How do you most frequently receive patient discharge information?

|  |  | Paper |
| --- | --- | --- |
|  |  | Fax |
|  |  | Email |
|  |  | Provider access to credential/ access portal |
|  |  | Imported to EHR (Electronic Health Record) |
|  |  | Other:  |

4. When do you receive patient discharge information?

|  | Before the patient arrives |
| --- | --- |
|  | As the patient arrives/ With the patient |
|  | After the plan of care was developed |
|  | Other:  |

5. On average, how many hospital admissions and/or readmissions do you receive monthly? *(i.e., new admissions of patients getting discharged from acute care and re-admissions of patients who are returning from the hospital)*

|  | less than 30 |
| --- | --- |
|  | 30-60 |
|  | greater than 60 |

6. How many different referring hospitals do you typically receive admissions from?

|  | 1-2 |
| --- | --- |
|  | 3-4 |
|  | 5 or more |

7. For each of the items listed below that may be included in a discharge document, please select the box that best describes the usefulness of the item

Necessary for all clinicians Helpful but not always necessary Not necessary

| Admission diagnosis |  |  |  |
| --- | --- | --- | --- |
| Date of admission/discharge |  |  |  |
| Principal/Primary diagnosis *(Diagnosis responsible for the largest portion of the patient's stay)* |  |  |  |
| Patient identifiers *(E.g.: DOB, Name, MRN)* |  |  |  |
| Patient demographics *(E.g.: Address, Phone number, Marital status)* |  |  |  |
| History of present illness for hospitalization |  |  |  |
| Medication on admission |  |  |  |

| Family history |  |  |  |
| --- | --- | --- | --- |
| Social and lifestyle history |  |  |  |
| Physical findings relevant at discharge |  |  |  |
| Hospital course *(A description of the events occurring to a patient during his/her hospital stay)* |  |  |  |
| Procedures performed in hospital |  |  |  |
| Laboratory tests and investigation results *(Including pending results)* |  |  |  |
| Changes in medication during patient’s stay in hospital |  |  |  |
| Discharge status/Patient’s discharge condition (Refers to how the patient is doing at discharge or the patient’s health status on discharge) |  |  |  |
| Patient’s: physical and cognitive functional ability at discharge |  |  |  |
| Patient’s: physical and cognitive functional ability at admission |  |  |  |
| Nutritional status at discharge |  |  |  |
| Nutritional status at admission |  |  |  |
| List of discharge diagnoses |  |  |  |
| Discharge instructions |  |  |  |
| Discharge medications |  |  |  |
| Follow-up issues |  |  |  |
| Appointments after discharge |  |  |  |
| Goals of care and treatment plan during hospital stay |  |  |  |
| Life-sustaining treatment preferences |  |  |  |
| Free-text comments (A field for clinicians to share miscellaneous notes about the patient) |  |  |  |
| Discharging physician contact information |  |  |  |
| Contact information for doctors who consulted patient in hospital |  |  |  |
| Emergency contact information |  |  |  |
| Adverse reactions during medical stay *(Including allergies to medication)* |  |  |  |
| Immunization |  |  |  |

8. What important information do you find missing from discharge documents?

9. Is there any information that should be eliminated from discharge documents?

10. Is there anything else about discharge documents you would like us to know?

11. Would you like a copy of our survey results?

|  | Yes |
| --- | --- |
|  | No |
