# Supplementary material for: Usability of Electronic Health Record–Generated Discharge Summaries: Heuristic Evaluation
Source: J Med Internet Res. 2021 Apr 15;23(4):e25657. doi: 10.2196/25657 (PMC8085750; doi:10.2196/25657)
Supplement: Multimedia Appendix 5 [file jmir_v23i4e25657_app5.pdf]

# Hospital 1, Patient 1 (H1P1)

*** Final Report ***

This note was dictated using Dragon voice recognition
Admission Date
X/5/2019
Discharge X/20/2019

**Admission Information**
Patient 1 is a xx year-old male with past medical history significant for active tobacco abuse, COPD on 2-3L home oxygen, diabetes, hypertension, coronary artery disease s/p CABG x5, morbid obesity, urinary tract obstruction with chronic indwelling Foley catheters the past 2 years and multiple prior abdominal surgeries who was transferred from HospitalABC X/5/2019 where he presented initially to his primary care doctor with 2 days of decreased p.o. intake, focal abdominal discomfort and malaise. He was sent from his primary care office to the ER at HospitalABC for a CAT scan which showed evidence of incarcerated ventral wall hernia. He was transferred down to Hospital1 for definitive surgical care. Initially had a white blood cell count of 14,000 and a creatinine of 2.34 complained of moderate abdominal pain.

**Hospital Course**
Patient presented in transfer from HospitalABC on X/5/2019 with incarcerated ventral wall hernia with obstruction. On X/6 he was brought to the operating room for exploratory laparotomy with lysis of adhesions and partial resection of the transverse colon as well as to small bowel resection secondary to enterotomies. His abdomen was left open at that time due to instability.

Postoperatively he was brought to the ICU intubated in shock on pressors and mechanically ventilated. On X/7 he was brought back to the operating room for removal of wound VAC/abthera, abdominal exploration with resection of cecum and remaining ascending and proximal transverse colon, resection of portion of distal transverse colon, resection of approximately 1 foot of distal small bowel, creation of end ileostomy. Fascia was closed and abdominal wound remained open with packing for dressing changes.

Patient remained intubated to the ICU. He continued on pressors, IV fluid hydration and broad-spectrum antibiotics. Vent weaning was initiated. A feeding tube was eventually placed and started to trickle tube feeds. He eventually weaned off pressors. He was able to get out of bed to chair. VAC dressing was placed on the abdominal wound on X/11/2019. Tube feeds were advanced to goal. He completed a 7-day course of Zosyn for enterococcus and Klebsiella in his urine. He required bronchoscopy on X/12. Patient was successfully extubated on X/13 to high flow nasal cannula. Feeding tube was removed. Patient passed swallow exam and diet was slowly advance started with clear liquids. He continued to undergo weaning of nasal oxygen with goal to achieve his baseline 2-3 L/min.

The patient demonstrated some intermittent oxygen desaturations and did have a sleep study during his stay. He was diagnosed with mild obstructive sleep apnea with some prolonged episodes of apnea, CPAP was recommended but the patient declined and was not interested in any follow-up with sleep medicine but rather prefers to continue on his usual 3 L nasal cannula supplemental oxygen.

He continued to have adequate ostomy output. He is currently tolerating a consistent carbohydrate diet, his caloric/protein take is still below his recommended amount and supplements have been recommended.

Given his multiple comorbidities, he was evaluated by palliative care during his stay. He was made a DNR. He will have ongoing palliative care as an outpatient. He was started on nystatin swish and swallow for oral thrush and should complete a 7-day course.

He was felt need of ongoing rehabilitation for physical therapy and Occupational Therapy and will go to a skilled nursing facility.

**Significant Findings**

*Incidental radiographic findings: Colonic diverticula, osteopenia*

*Intra-Op findings:*
**X/6:**
1. Small portion of transverse colon incarcerated, strangulated, with perforation in an incisional hernia
2. Large piece of mesh, multiple incisional hernias found at edges of mesh
3. Extensive adhesions between small bowel and abdominal wall and mesh, numerous enterotomies

**X/7:**
Patient had a very short remaining proximal colonic segment, the distal transverse colonic segment looked mildly cyanotic, the small bowel appeared viable

**Reason For Exam**
Diminished pulses
**VL Lower Ext Arterial ABI/TBI Limited**
Ordered By: ClinicianA
**********************************
SUMMARY:
**********************************
Limited doppler waveforms focally at the ankle, ankle-brachial indices, and toe-brachial indices were measured. Suboptimal flow signals were obtained bilaterally. The ankle-brachial indices are consistent with moderate arterial insufficiency at .5 on the right, and consistent with moderate arterial insufficiency at .52 on the left. Toe-brachial indices are consistent with moderately severe insufficiency at .35 on the right, and consistent with moderately severe insufficiency at .33 on the left.

# Hospital 1, Patient 1 (H1P1)

Procedures and Treatment Provided
**Procedure Date**
X/6/2019
**Procedure**
Expiratory laparotomy, extensive lysis of adhesions, partial resection of transverse colon, small bowel resection x2, small bowel anastomosis x2, placement of abdominal VAC dressing
**Operator**
OperatorA
**Preoperative Diagnosis**
Incarcerated incisional hernia with transverse colon, strangulated
**Postoperative Diagnosis**
Same

**Procedure Date**
X/7/2019
**Procedure**
Removal of wound VAC/abthera, abdominal exploration resection of cecum and remaining ascending and proximal transverse colon, resection of portion of distal transverse colon, resection of approximately 1 foot of distal small bowel, creation of end ileostomy
**Operator**
OperatorB
**Preoperative Diagnosis**
Second look abdominal exploration after previous day surgery for strangulated perforated transverse colon and ventral hernia
**Postoperative Diagnosis**
Same

**Physical Exam**
Vitals & Measurements
**Temperature**: 36.8 $^{o}$C (Tympanic) **TMIN**: 36.7 $^{o}$C (Oral) **TMAX**: 37.1 $^{o}$C (Oral) **Heart Rate**: 72(Monitored) **RR**: 20 **Blood Pressure**: 135/72 **Sp02**: 98% **Weight**: 96.8 kg
GEN: Awake, alert, conversant, no acute distress
PULM: Clear to auscultation bilaterally, non labored breathing on high flow nasal cannula
CV: Regular rate and rhythm. Normal S1 and S2
GI: Obese, soft, approximately tender around wound vac, scant serosanguineous from the VAC, ileostomy pink and viable with gas and liquid stool in bag
EXT: Warm and well perfused, 1+ peripheral edema

**Discharge Diagnoses and Plan**
1. Incarcerated ventral hernia
   Status post exploratory laparotomy with small bowel and colonic resection, creation of end ileostomy as noted above.
   Patient has progressed well post-operatively, is tolerating a consistent carbohydrate with supplements. He is not consistently meeting all of his nutritional requirements by mouth at this time, his ileostomy is functioning well.
   Encourage PO intake, Ensure supplementation to make sure he is meeting his caloric and protein needs.
   Follow-up with Hospital1 surgical associates for a postop check on with ClinicianB on X/27 at 3 PM.
2. COPD without exacerbation
   Improving oxygenation, now on baseline supplemental oxygen.
   Has been followed by pulmonary medicine as an inpatient, has been started on Splriva.
   Should have outpatient follow-up with pulmonary medicine either locally or here at Hospital1 in 4 to 6 weeks after discharge for PFTs and adjustment of medications. This appointment will need to be scheduled.
3. Diabetes mellitus
   Hemoglobin A1c was 5.7. He was managed with sliding scale insulin during his stay.
   Resume his usual home does of metformin.
4. Obstruction of urinary tract
   Chronic.
   Has indwelling Foley catheter which is managed at HospitalDEF with monthly catheter changes. He was due to get a suprapubic tube placed at HospitalDEF but ended up at Hospital1 with an acute incarcerated hernia. Foley catheter was changed by urology during his stay. Patient was noted to have some ventral urethral erosion likely from his indwelling Foley catheter. Continuing ongoing indwelling Foley catheter and can follow-up when stable at HospitalDEF for placement of suprapubic tube.
5. Small bowel obstruction
   Due to incarcerated ventral hernia as noted above.
6. Arteriosclerotic coronary artery disease
   Continued on aspirin and statin.
7. Morbid obesity
8. Personal history of smoking
   Managed with a nicotine patch during his stay.
9. Hypertension
   Well-controlled on his usual home medications including lisinogril and metoprolol.
10. Urinary tract infection + Kleb, enterococcus
    Completed a course of antibiotics
11. Obstructive sleep apnea
    Newly diagnosed during the stay, CPAP recommended but the patient declined, he also declined follow-up with sleep medicine.

# Hospital 1, Patient 1 (H1P1)

Discharge Medications

Home

- acetaminophen 500 mg oral tablet, 1000 mg= 2 tab, Oral, Every 6 hr, PRN
- amLODIPine 10 mg oral tablet, 10 mg= 1 tab, Oral, Daily
- aspirin 81 mg oral delayed release tablet, 81 mg = 1 tab, Oral, Daily
- insulin lispro 100 units/mL injectable solution, 0-14 units, Subcutaneous, 4 times/day (before meals & at bed)
- Lisinopril 20 mg oral tablet, 20 mg= 1 tab, Oral, Daily
- metFORMIN 500 mg oral tablet, 500 mg = 1 tab, Oral, 2 times/day
- metoprolol succinate 50 mg oral capsule, extended release, 50 mg = 1 cap, Oral, Daily
- nystatin 100,000 units/g topical powder, 1 app, Topical, 3 times/day
- nystatin 100,000 units/mL oral suspension, 500000= 5 mL, Oral, 4 times/day
- oxybutynin 10 mg/24 hr oral tablet, extended release, 10 mg = 1 tab, Oral, Daily
- polyethylene glycol 3350, Oral, Daily, PRN
- pravastatin 20 mg oral tablet, 20 mg = 1 tab, Oral, At bedtime daily
- umeclidinium 62.5 mcg/inh inhalation powder, 62.5 mcg = 1 puffs, Inhalation, Daily
- zinc sulfate 220 mg oral capsule, 220 mg = 1 cap, small bowel feeding tube, 2 times/day

Patient Discharge Condition
Stable

Discharge Disposition
To skilled nursing facility

Time Spent on Discharge
Greater than 30 minutes

**Signature Line**

Electronically Signed on X/22/19 06:59 AM

xxxSIGNATURExxx________________________________________

Electronically Signed on X/20/19 06:59 AM

xxxSIGNATURExxx________________________________________

Result type: Discharge Summary
Result date: X 20, 2019 11:58 EDT
Result status: Auth (Verified)
Result title: Discharge Summary
Performed by: Clinicianxxx on X 18, 2019 14:35 EDT
Verified by: Clinicianxyx on X 20, 2019 11:58 EDT
Encounter info: 1234567890, Hospital1, Inpatient, X/05/19 – X/20/19
