# Supplementary material for: Usability of Electronic Health Record–Generated Discharge Summaries: Heuristic Evaluation
Source: J Med Internet Res. 2021 Apr 15;23(4):e25657. doi: 10.2196/25657 (PMC8085750; doi:10.2196/25657)
Supplement: Multimedia Appendix 6 [file jmir_v23i4e25657_app6.pdf]

Hospital 1, Patient 2 (H1P2)

*** Final Report ***

This note was dictated using Dragon voice recognition
Admission Date
x/21/19

Discharge Date
x/25/19

Discharge Diagnosis
1. Alcohol withdrawal, resolved
2. Rapid atrial fibrillation, resolved
3. Emphysema/COPD- in acute exacerbation
4. Hepatitis C, chronic

Admission Information
This is a XX-year-old male who had a past medical history significant for prior CVA, hepatitis C, hyperlipidemia, COPD, paroxysmal atrial fibrillation on apixaban in addition to alcohol abuse amongst other issues who presented to HOSPITAL1 ER after being found on the floor by his friend "not acting like himself." The patient was reportedly covered in his stool on his legs but there is no noted stool around his buttocks according to the ER provider. He came in with global weakness and had to be assisted from a private vehicle in front of the emergency room into the waiting room. He was reportedly last seen "completely normal" a few days ago. When asked how much the patient drinks on normal basis he states he only drinks 1 beer a day. He came into the hospital with an alcohol level of almost 500. He states that he "lives in the woods with his son." He otherwise is unable to provide much in the way of history surrounding the events today. He is very adamant that he only drinks "one beer per day." When further questioned on this he states that he may actually drink 2 sixpacks of beer a day. It is unclear if this is accurate information. The patient denies any difficulty breathing. He denies any chest pain. He states he takes his apixaban as prescribed for his history of atrial fibrillation. He notes that he smokes about 1 pack of cigarettes a day.

Of note the patient had a recent hospital stay from xx 26 to xx 4. During that hospitalization he was brought in by EMS unresponsive and only making grunting noises. He ended up being intubated and had some episodes of hypotension and bradycardia. He had a urine fentanyl screen which was positive. He went into alcohol withdrawal and was eventually placed on a Precedex drip. He was eventually discharged home. [1] The emergency department he was found to have an ethanol level of 475, CT of the head showed no acute intracranial pathology. He is acutely intoxicated with alcohol and previously had required Precedex drip for withdrawal. He was alert and oriented despite having such a high alcohol level. He was found to be in rapid atrial fibrillation with RVR and required IV diltiazem. He was also found to have an acute COPD exacerbation, he was admitted to the hospitalist service for further evaluation.

Hospital Course
Alcohol Withdrawal: Patient started to score on CIWA scale and started to withdrawal from alcohol while inpatient. He continued to score for alcohol withdrawal and required as needed Ativan. He was seen by substance use services, he was given information regarding outpatient services.
Acute COPD exacerbation: He required IV steroids for COPD exacerbation, his steroids were switched to oral and his breathing improved. He was discharged with a prednisone taper
Atrial Fibrillation with RVR: His rapid atrial fibrillation was likely secondary to his acute alcohol use and COPD exacerbation. He was restarted on his home medications of metoprolol and diltiazem with improvement in his rate control.

He was having significant gait instability and needed to be evaluated by PT/OT. Unfortunately his gait instability continued to persist and PT and OT therapies recommended SNF. He was cleared by speech therapy. Patient was adamant that he would not be discharged to SNF and decided to go home with VNA nursing, PT and OT services.

Significant Findings
Labs (Last four charted values)

**WBC**	5.84 (XX 24)	6.33 (XX 22)	C **1.44** (XX 21)	4.34 (XX 20)
**Hgb**	L **11.6** (XX 24)	L **12.2** (XX 22)	L **11.8** (XX 21)	13.5 (XX 20)
**Hct**	L **33.9** (XX 24)	L **34.8** (XX 22)	L **34.8** (XX 21)	38.6 (XX 20)
**Plt**	L **90** (XX 24)	L **105** (XX 22)	L **102** (XX 21)	L **116** (XX 20)
**Na**	136 (XX 25)	L **133** (XX 24)	L **132** (XX 23)	L **134** (XX 22)
**K**	L **3.2** (XX 25)	3.7 (XX 24)	3.6 (XX 23)	C **2.9** (XX 22)
**CO2**	25 (XX 25)	27 (XX 24)	28 (XX 23)	29 (XX 22)
**Cr**	L **0.52** (XX 25)	L **0.52** (XX 24)	L **0.57** (XX 23)	L **0.48** (XX 22)
**BUN**	18 (XX 25)	15 (XX 24)	14 (XX 23)	8 (XX 22)
**Glucose Random**	H **118** (XX 25)	H **117** (XX 24)	H **116** (XX 23)	H **170** (XX 22)
**PT**	H **12.7** (XX 20)			
**INR**	1.2 (XX 20)			

CT Brain/Head w/o Contrast
Ordered By: ClinicianA

HISTORY: Altered level of consciousness

COMPARISON: CT brain YY 25, 2019

TECHNIQUE: Axial CT images of the brain are acquired without intravenous contrast. Coronal and sagittal reformats are obtained.

Radiation dose limiting techniques utilized include automated exposure control and iterative reconstruction.

FINDINGS:
There are scattered mostly periventricular white matter hypodensities compatible with chronic small vessel disease. There is chronic diffuse parenchymal volume loss. There is atherosclerosis of the carotid arteries. There are no signs of acute large vascular territory infarction, hemorrhage, nor extra-axial collection. No herniation, hydrocephalus, no significant positive mass effect. Paranasal sinuses are well aerated. Chronic changes to the right mastoids with small mastoid fluid. No evidence of acute osseous abnormality. Bones are osteopenic. No suspicious orbital lesions. Missing teeth in mandible and maxilla.

IMPRESSION:
1. Signs of chronic small vessel disease, volume loss, atherosclerosis. No superimposed acute intracranial pathology. If symptoms persist MRI may prove useful.
[2]

Hospital 1, Patient 2 (H1P2)

XR Chest 2 Views
Ordered By: ClinicianB
STUDY: XR Chest 2 Views

INDICATION: Difficulty breathing.

TECHNIQUE: PA and lateral views of the chest.

COMPARISON: CT pulmonary angiogram XX/26/2019 and chest radiograph of XX/25/2019.

FINDINGS:
The cardiac silhouette is within normal limits for size. Apical lucencies are present consistent with emphysema. There is no consolidation, pulmonary edema, pleural effusion, or pneumothorax. No acute osseous abnormality is identified.

IMPRESSION:
No radiographic acute cardiopulmonary abnormality.
[3]

Physical Exam

Vitals & Measurements
Temperature: 36.5 °C (Oral) **TMIN**: 36.4 °C (Oral) **TMAX**: 36.6 °C (Oral) **Heart Rate**: 78(Monitored) **RR**: 20 **Blood Pressure**: 120/93 **Sp02**: 97%
Constitutional: Alert, no acute distress. Appears tired but is answering questions appropriately.
Skin: normal color, no rashes, warm.
Neck: supple, no adenopathy.
Cardiovascular: Irregularly irregular
Respiratory: Lung sounds are diminished but clear, few scattered wheezes.
Abdomen: nondistended, nontender, no guarding, normal BS.
Excremities: full joint motion, no peripheral edema
Psych: Affect and mood appropriate.

Discharge Medications
Home
acetaminophen 325 mg oral tablet, 650 mg= 2 tab, Oral, Every 6 hr, PRN
apixaban 5 mg oral tablet, 5 mg=1 tab, Oral, 2 times/day
Cardizem CD 120 mg/24 hours oral capsule, extended release, 120 mg= 1 cap, Oral 2 times/day
metoprolol tartrate 50 mg oral tablet, 50 mg= 1 tab, Oral, 2 times/day
multivitamin adult, oral tablet, 1 tab, Oral, Every morning
perdiSONE 20 mg oral tablet, See Instructions
ProAir HFA 90 mcg/inh inhalation aerosol, 1-2 puffs, Inhalation, Every 6 hr, PRN
thlamine 100 mg oral tablet, 100 mg = 1 tab, Oral, Daily

Patient Discharge Condition
Stable, Improved

Discharge Disposition
Home with services, patient declined SNF

Discharge instructions:
-Diet: House
-Discussed worsening signs or symptoms and when to return to the ED or call PCP.

Discharge Medication changes:
Follow up: XX/31/2019 @ 9AM

Time Spent on Discharge
35 minutes

[1] Admission H&P clinicianXYZ XX/20/2019 21:11 EDT
[2] CT Brain/Head w/o Contrast clinicianXYZ XX/20/2019 19:00 EDT
[3] XR Chest 2 Views; clinicianXYZ XX/20/2019 21:54 EDT

Signature Line
Electronically Signed on XX/25/19 01:53 PM

xxxSIGNATURExxx

Result type: Discharge Summary
Result date: XX 25, 2019 13:40 EDT
Result status: Auth (Verified)
Result title: Discharge Summary
Performed by: Clinicianxxx on XX 25, 2019 13:53 EDT
Verified by: Clinicianxxx on XX 25, 2019 13:53 EDT
Encounter info: 9876543210, Hospital1, Inpatient, XX/20/19 – XX/25/19
