# Supplementary material for: Usability of Electronic Health Record–Generated Discharge Summaries: Heuristic Evaluation
Source: J Med Internet Res. 2021 Apr 15;23(4):e25657. doi: 10.2196/25657 (PMC8085750; doi:10.2196/25657)
Supplement: Multimedia Appendix 7 [file jmir_v23i4e25657_app7.pdf]

ChartH2P1

MRN: XXXXXXXX

XXXXXXXX, MD
Physician
Cardiology

Discharge Summary
Signed

Note Time: xx/xx/xxxx 11:09 AM
Creation Time: xx/xx/xxxx 11:09 AM

Physician Discharge Summary

Pt Name ChartH2P1 **MRN:** XXXXXXXX
Age: xx y.o. **DOB:** xx/xx/xxxx

Admit date: xx/xx/xxxx 3:11 PM

Discharge date: xx/xx/xxxx

Primary Care Physician:
XXXXXXXX, MD
Address Removed
Phone: xxx-xxx-xxxx
Fax: xxx-xxx-xxxx

Allergies: Patient has no known allergies.

Admitting Physician: XXXXXXXX, MD

Discharge Physician: XXXXXXXX, MD

Admission Diagnoses: NSTEMI (non-ST elevated myocardial infarction) (HCC) [I21.4]

Discharge Diagnoses:
1. Late presentation of MI now s/p 2V CABG (LIMA-LAD and SVG-OM)
2. 2D echo xx/xx/xxxx with EF 45-50% (improved from Preop EF 40%)
3. Hypotension-improving
4. Post op AFib x several days
5. Leukocytosis

Hospital Course:
Ms. XXXXXXXX is a xx year old female who presented as transfer due to NSTEMI. She underwent cardiac cath on xx/xx/xxxx which showed severe distal LM and LAD/diagonal disease, mild LV dysfunction with severe antero-lateral hypokinesis- EF 40%. CT surgery was consulted and she underwent CABG x 2 (LIMA-LAD, SVG-OM) on xx/xx/xxxx. 2D echo post surgery did show some improvement of EF, with LV function 45-50%. Post op stay was complicated by Hypotension, AKI and low urine output, all of which have improved. However, she does remain mildly hypotensive at times (SBP 90s-100s), though asymptomatic. In addition, she went into post op Afib. She was started on amiodarone and oral anticoagulation with coumadin. CT surgery prefers use of coumadin for first 4 weeks post surgery. After that time, patient can be switched to DOAC if needed. She had no complaints of chest pain or SOB.

Significant Diagnostic Studies:

1. CXR xx/xx/xxxx stable, mild bibasilar opacities.
2. 2D echo xx/xx/xxxx showing 45-50%, small hematoma overlying RV but it is difficult to distinguish from pericardial fat, mild global hypokenesis, mod- severe TR

Disposition: to rehab
Patient Instructions:
Discharge Medications:
Your Current Medication List

**acetaminophen (TYLENOL) 325 mg Oral tablet (Taking)**	take 2 Tabs by mouth Every 6 hours as needed.
**AMIODArone (PACERONE) 400 mg Oral tablet (Taking)**	take 1 Tab by mouth Every 8 hours for 2 days.
**AMIODArone (PACERONE) 200 mg Oral tablet**	**Starting on xx/xx/xxxx.** take 1 Tab by mouth Daily.
**aspirin 81 mg Oral tablet**	**Starting on xx/xx/xxxx.** 1 Tab by chew and swallow route Daily.
**atorvastatin (LIPITOR) 80 mg Oral tablet (Taking)**	take 1 Tab by mouth Every evening.
**docusate sodium (COLACE) 100 mg Oral capsule (Taking)**	take 1 Cap by mouth 2 times daily.
**metoprolol succinate XL 25 mg extended-release tablet (Taking)**	take 1 Tab by mouth Daily.
**pantoprazole (PROTONIX) 40 mg Oral tablet**	**Starting on xx/xx/xxxx.** take 40 mg by mouth DAILY at 6AM.
**polyethylene glycol (MIRALAX) 17 gram Oral packet**	**Starting on xx/xx/xxxx.** take 1 Packet by mouth Daily.
**warfarin (COUMADIN) 2.5 mg Oral tablet (Taking)**	take 1 Tab by mouth Daily.
**estrogens, conjugated, (PREMARIN) 0.625 mg/gram Vaginal crea (Taking)**	by Vaginal route. As instructed

Follow-up information:
Follow-up Information
XXXXXXXX, MD. Schedule an appointment as soon as possible for a visit in 2 weeks.
Specialty: REFERRING
Contact information
Address Removed
xxx-xxx-xxxx

Please follow up.
Why: need labwork: bmp/cbc in 1 week; PT/INR on xx/xx/xxxx

XXXXXXXX, APN On xx/xx/xxxx.
Why: at 2pm
Contact information
Address Removed

Phone xxx.xxx.xxxx

XXXXXXXX, MD. Go on xx/xx/xxx.
Specialty: SURGERY,CARDIOTHORACIC
Why: @ 11:30 am.
Contact information
Address Removed
xxx.xxx.xxx

Additional Providers to receive this summary:

Signed:
XXXXXXXX, APN
xx/xx/xxx
11:09 AM
