# Supplementary material for: Usability of Electronic Health Record–Generated Discharge Summaries: Heuristic Evaluation
Source: J Med Internet Res. 2021 Apr 15;23(4):e25657. doi: 10.2196/25657 (PMC8085750; doi:10.2196/25657)
Supplement: Multimedia Appendix 8 [file jmir_v23i4e25657_app8.pdf]

ChartH2P2

MRN: XXXXXXXX

XXXXXXXX, MD
Physician
Hospitalist

Discharge Summary
Signed

Note Time: xx/xx/xxxx 1:42 PM
Creation Time: xx/xx/xxxx 1:42 PM

Physician Discharge Summary

Pt Name: ChartH2P2

MRN: XXXXXXXX

Age: xx y.o.
DOB: xx/xx/xxxx

Admit date: xx/xx/xxxx 8:13 PM

Discharge date: xx/xx/xxxx

Primary Care Physician:
Physician, No Pcp
Phone: xxx-xxx-xxxx
Fax: xxx-xxx-xxxx

Allergies: Patient has no known allergies.

Admitting Physician: XXXXXXXX, DO

Discharge Physician: XXXXXXXX, MD

Admission Diagnoses: Hip fracture (HCC) [S72.009A]

Discharge Diagnoses:

Active Hospital Problems

Diagnosis

- *Femur fracture, left (HCC)

Resolved Hospital Problems

Diagnosis

No resolved problems to display.

Admitting Condition: poor

Discharged Condition: good

Hospital Course: XXXXXXXX is an xx y.o. xxxxx female w/ PMHx of HTN and rheumatic fever who presented with left proximal intertrochanteric comminuted fracture after mechanical fall now s/p cephalomedullary nail (xx/xx). Patient was in her backyard and she picked up a ball and lost her balance when she threw the ball back. Patient lives alone and is able to carry out most of her ADLS. Post-operatively patient required 1 unit PRBC with appropriate response in hemoglobin. On admission patient was also found to be hypotensive with AKI, likely pre-renal. Home anti-hypertensives were held.

Acute pathologic left Hip Fracture
- POD #3 s/p L hip cephalomedullary nail
- pain control
- vitamin D, TSH, PTH all within normal limits
- PT consult, WBAT
- pending rehab placement

Acute urinary retention:
- likely related to post-op as patient has been immobile as well as pain meds
- repeat bladder scans and straight cath as needed
- may require foley catheter

AKI- resolved
- baseline Cr 0.8
- likely pre-renal in the setting of post-op and relative hypotension
- gentle hydration
- continue to monitor

Acute blood loss Anemia:
- normocytic at baseline
- exacerbated post-op
- continue to monitor
- tranfuse for Hgb <7

Hx of HTN
- home regimen metoprolol 50 mg BID, HCTZ 25 mg, amlodipine 2.5 mg
- initially hypotensive so BP meds held, will slowly restart as BP tolerates

Significant Diagnostic Studies:

XR FEMUR LEFT 2 VW

Final Result

Intraoperative and postoperative radiographs demonstrating open reduction internal fixation of left femoral intertrochanteric neck fracture.

Signing Provider: XXXXXXXX

XR FEMUR LEFT 2 VW

Final Result

Intraoperative and postoperative radiographs demonstrating open reduction internal fixation of left femoral intertrochanteric neck fracture.

Signing Provider: XXXXXXXX

XR TECH TIME NO IMAGING 60 MINUTES

Final Result

XR TECH TIME NO IMAGING 60 MINUTES

Final Result

XR TECH TIME NO IMAGING 60 MINUTES

Final Result

STORE IMAGES OUTSIDE XRAY

Final Result

XR PORTABLE FEMUR 2 VIEWS LEFT
Final Result

Again seen is a comminuted intertrochanteric left femoral fracture
Signing Provider: XXXXXXXX

XR ELBOW LEFT 3 VW MIN
Final Result

No acute osseous abnormality.
Signing Provider: XXXXXXXX

CT ABDOMEN AND PELVIS W IV CONTRAST
Final Result

1. Acute, comminuted fracture of the left proximal femur intertrochanteric region, as above. Associated hematoma within the muscles of the left proximal thigh. There are a few areas of high density material associated with these hematomas, as detailed above. Differential diagnosis includes bony fragments versus small amount of contrast blush related to active hemorrhage

2. Low-density indeterminate nodule in the right adrenal gland measuring 1.5 cm, may be further evaluated with nonemergent multiphase CT or MRI.

3. Aneurysmal dilatation of the descending thoracic aorta measuring up to 3.8 cm

COMMUNICATION: Findings discussed with Dr. XXXXXXXX on xx/xx/xxxx at 9:22 PM

Signing Provider: XXXXXXXX

XR PORTABLE CHEST 1 VIEW ONLY
Final Result

No acute cardiopulmonary disease

Signing Provider: XXXXXXXX

Disposition: Data Unavailable
Patient Instructions:

Discharge Medications:
Your Current Medication List

**enoxaparin (LOVENOX) 40 mg/0.4 ml Subcutaneous syrg (Taking)**	40 mg by Subcutaneous route DAILY for 10 days.
**multivitamin Oral tablet (Taking)**	take 1 Tab by mouth Daily.
**CALCIUM ORAL (Taking)**	take 1 Tab by mouth DAILY.
**hydroCHLOROthiazide (HYDRODIURIL) 25 mg Oral tablet (Taking)**	take 25 mg by mouth Daily
**metoprolol TARTRATE 50 mg tablet (Taking)**	take 50 mg by mouth 2 times daily.
**amLODIPine (NORVASC) 2.5 mg Oral tablet (Taking)**	take 2.5 mg by mouth Daily.

Follow-up Procedures and tests:
No discharge procedures on file.

Activity: activity as tolerated

Diet: Regular Diet

Wound Care: None needed

Follow-up information:
Follow-up Information
Call Appointments Orthopaedics. Contact information
Address Removed
xxx-xxx-xxx

Additional Providers to receive this summary:

Signed:
XXXXXXXX, MD
xx/xx/xxx
1:42 PM
