# Supplementary material for: Usability of Electronic Health Record–Generated Discharge Summaries: Heuristic Evaluation
Source: J Med Internet Res. 2021 Apr 15;23(4):e25657. doi: 10.2196/25657 (PMC8085750; doi:10.2196/25657)
Supplement: Multimedia Appendix 9 [file jmir_v23i4e25657_app9.pptx]

## Slide 1
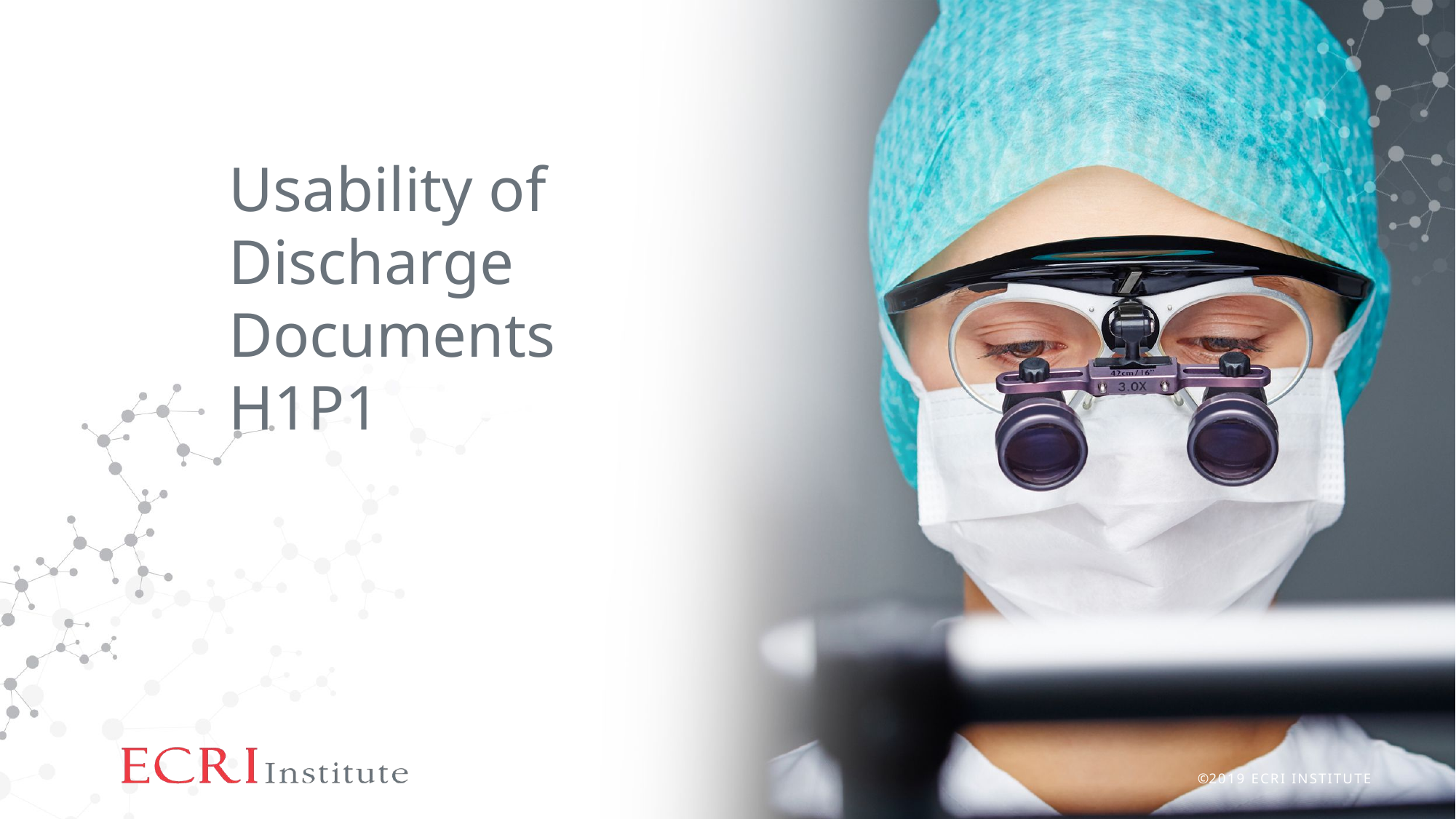

# Usability of Discharge Documents H1P1

## Slide 2
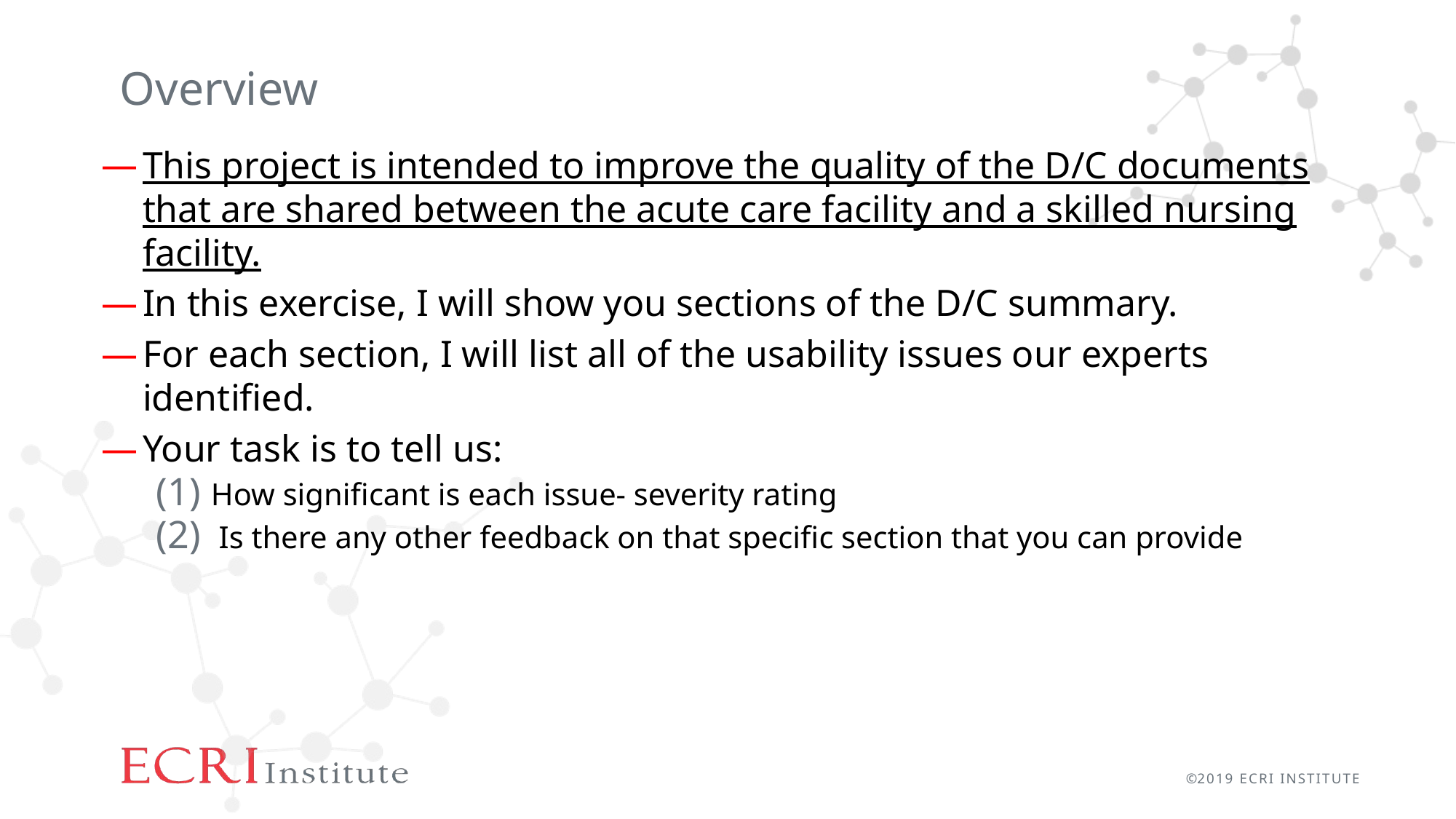

# Overview
This project is intended to improve the quality of the D/C documents that are shared between the acute care facility and a skilled nursing facility.
In this exercise, I will show you sections of the D/C summary.
For each section, I will list all of the usability issues our experts identified.
Your task is to tell us:
How significant is each issue- severity rating
 Is there any other feedback on that specific section that you can provide

## Slide 3
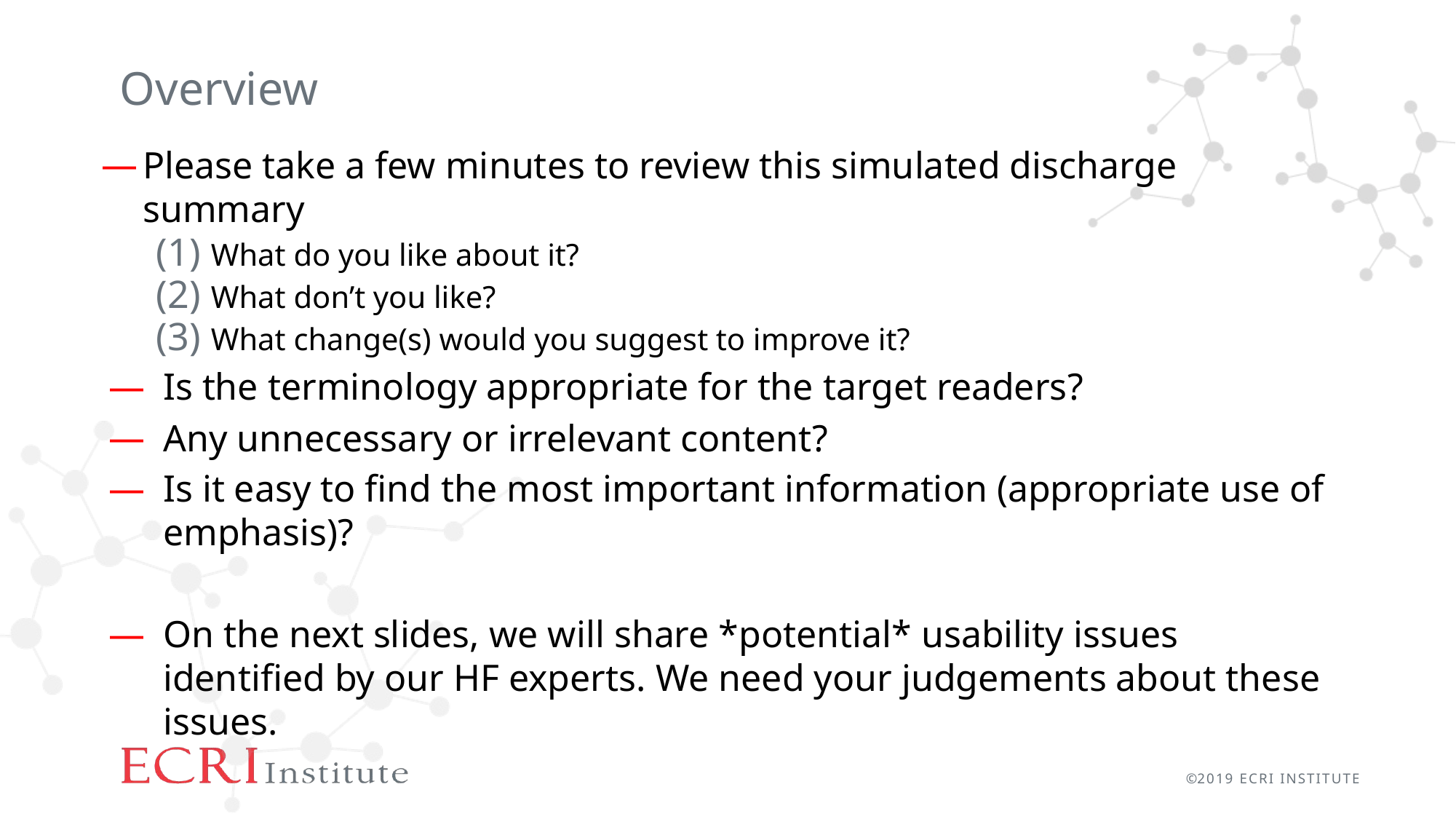

# Overview
Please take a few minutes to review this simulated discharge summary
What do you like about it?
What don’t you like?
What change(s) would you suggest to improve it?
Is the terminology appropriate for the target readers?
Any unnecessary or irrelevant content?
Is it easy to find the most important information (appropriate use of emphasis)?
On the next slides, we will share *potential* usability issues identified by our HF experts. We need your judgements about these issues.

## Slide 4
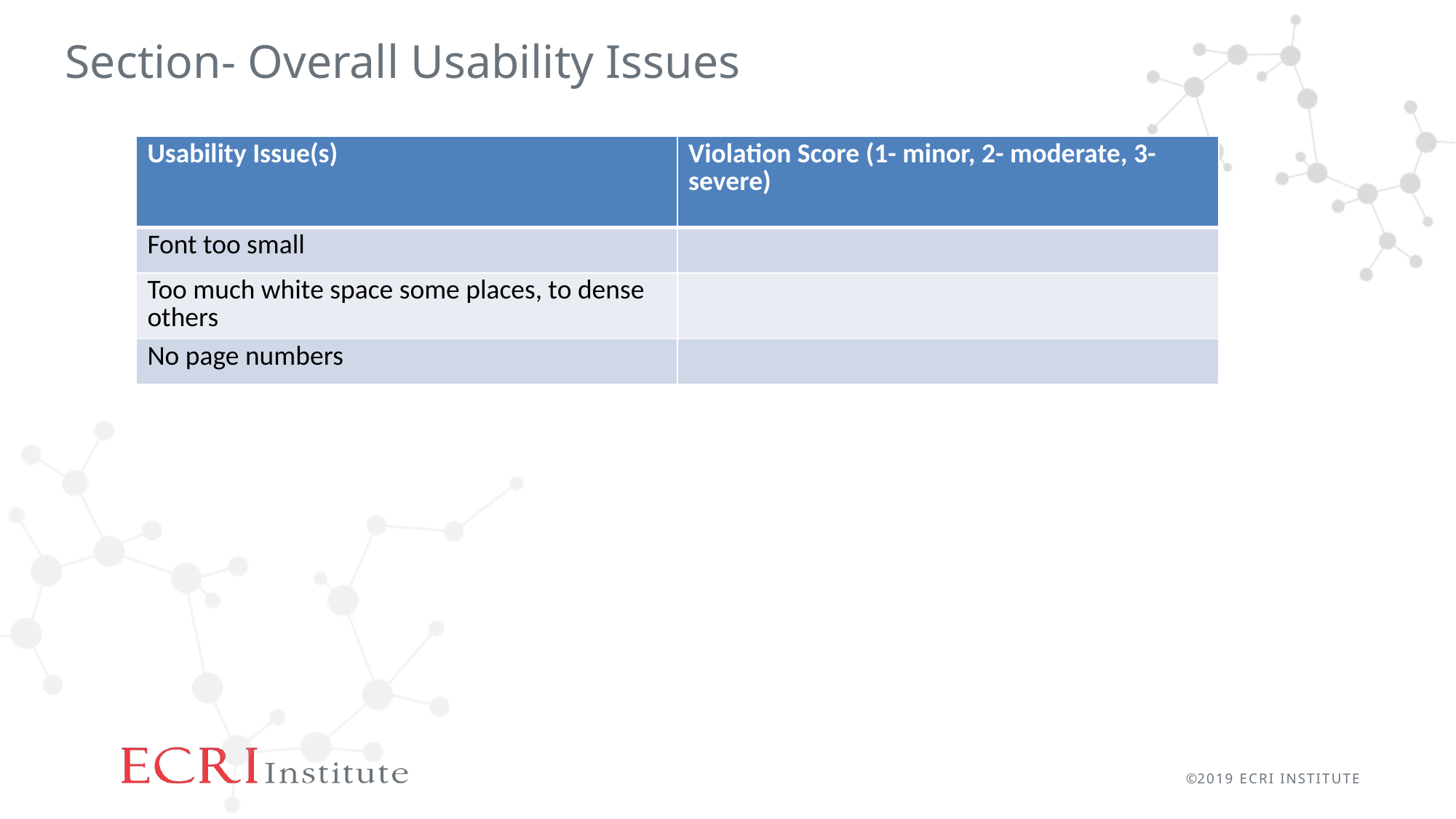

# Section- Overall Usability Issues
| Usability Issue(s) | Violation Score (1- minor, 2- moderate, 3- severe) |
| --- | --- |
| Font too small | |
| Too much white space some places, to dense others | |
| No page numbers | |

## Slide 5
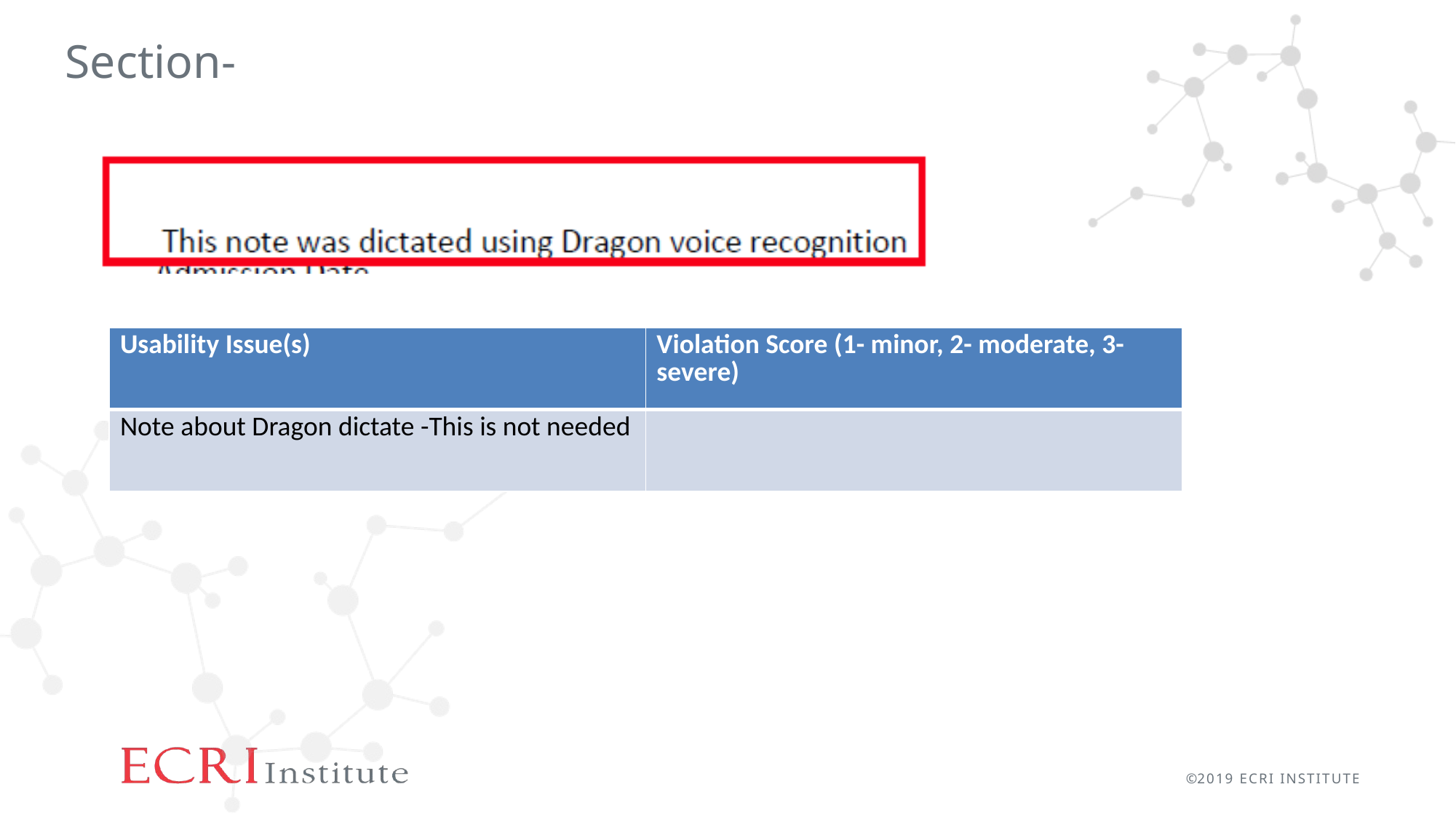

# Section-
| Usability Issue(s) | Violation Score (1- minor, 2- moderate, 3- severe) |
| --- | --- |
| Note about Dragon dictate -This is not needed | |

## Slide 6
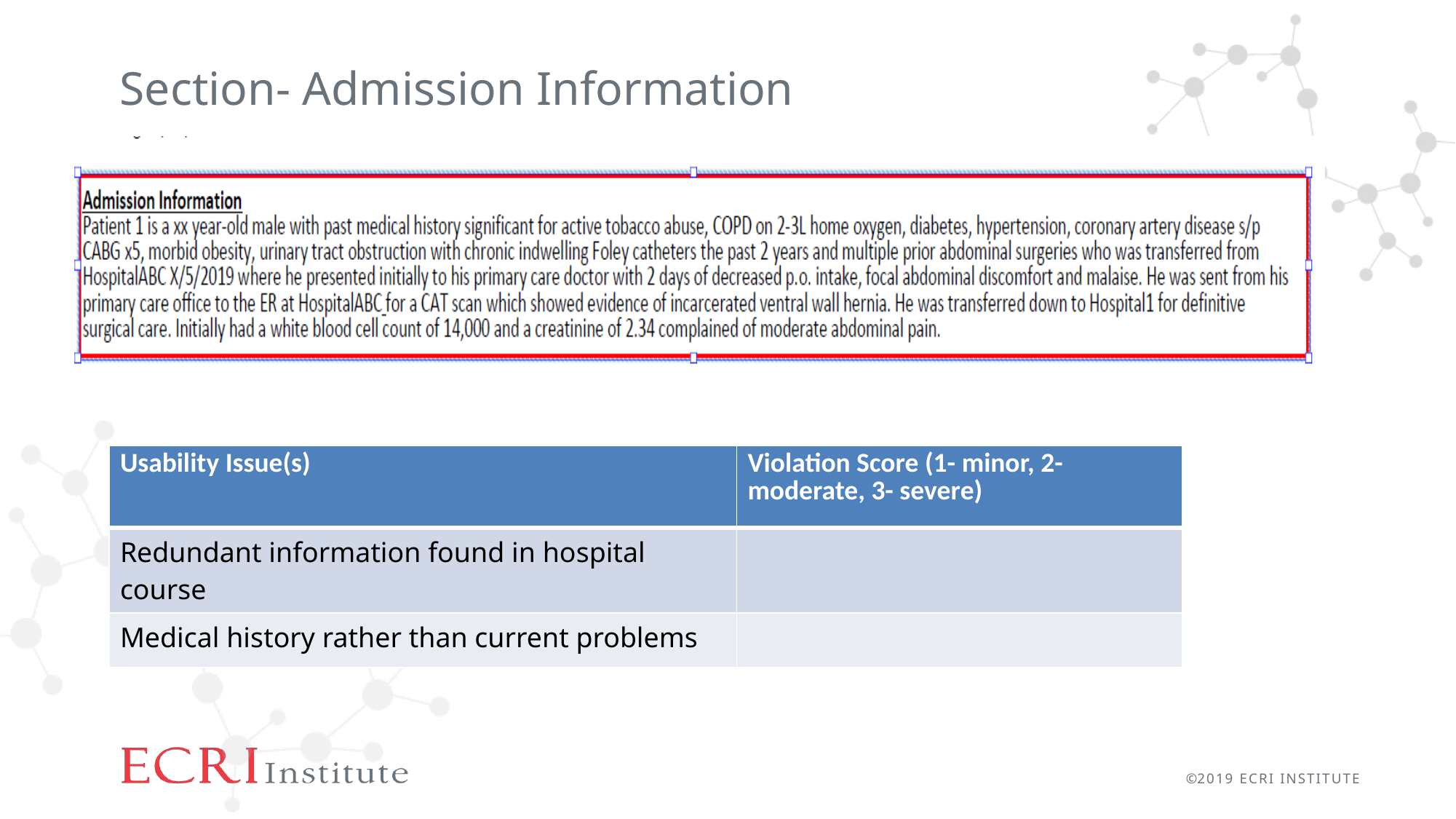

# Section- Admission Information
| Usability Issue(s) | Violation Score (1- minor, 2- moderate, 3- severe) |
| --- | --- |
| Redundant information found in hospital course | |
| Medical history rather than current problems | |

## Slide 7
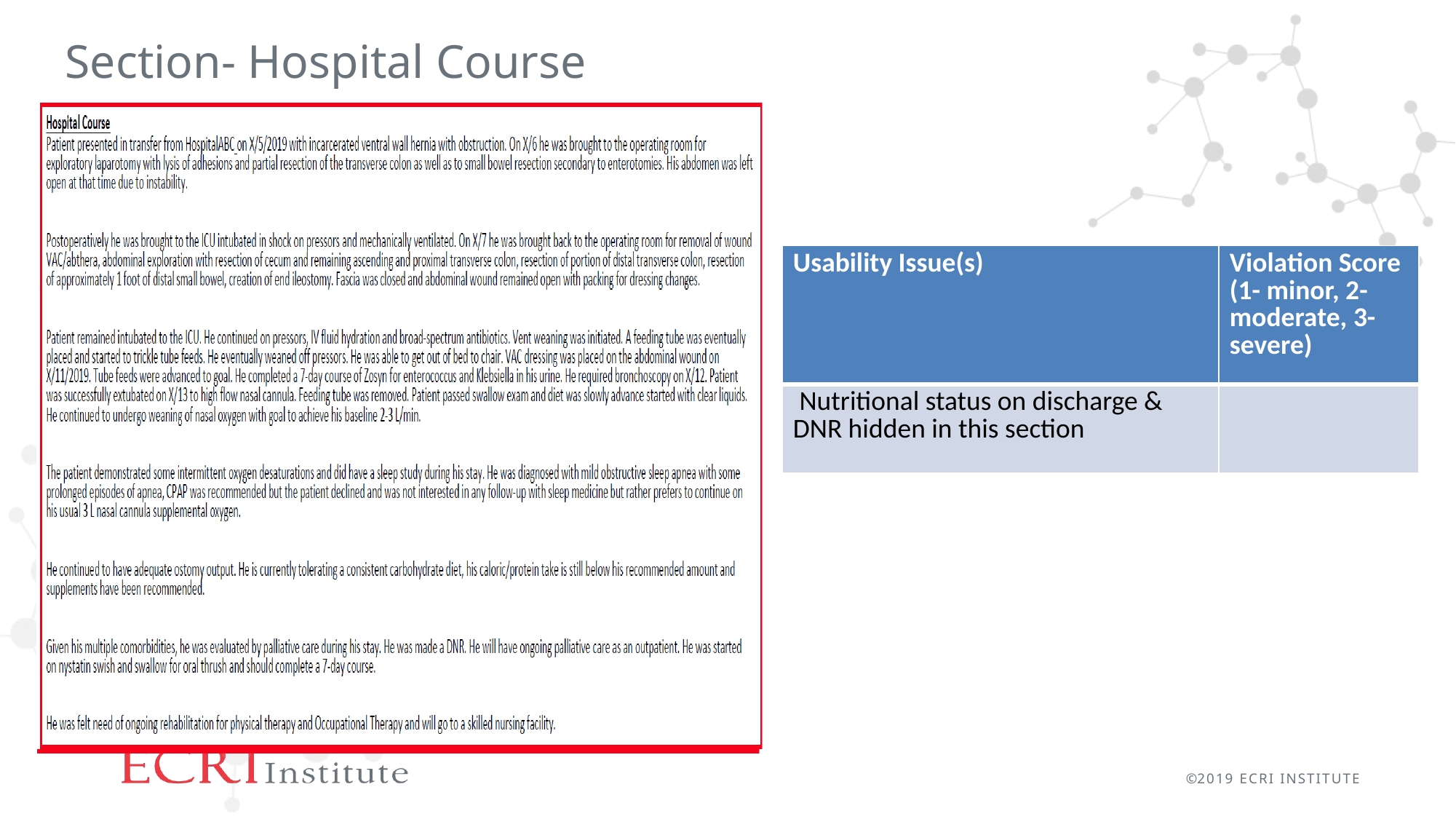

# Section- Hospital Course
| Usability Issue(s) | Violation Score (1- minor, 2- moderate, 3- severe) |
| --- | --- |
| Nutritional status on discharge & DNR hidden in this section | |

## Slide 8
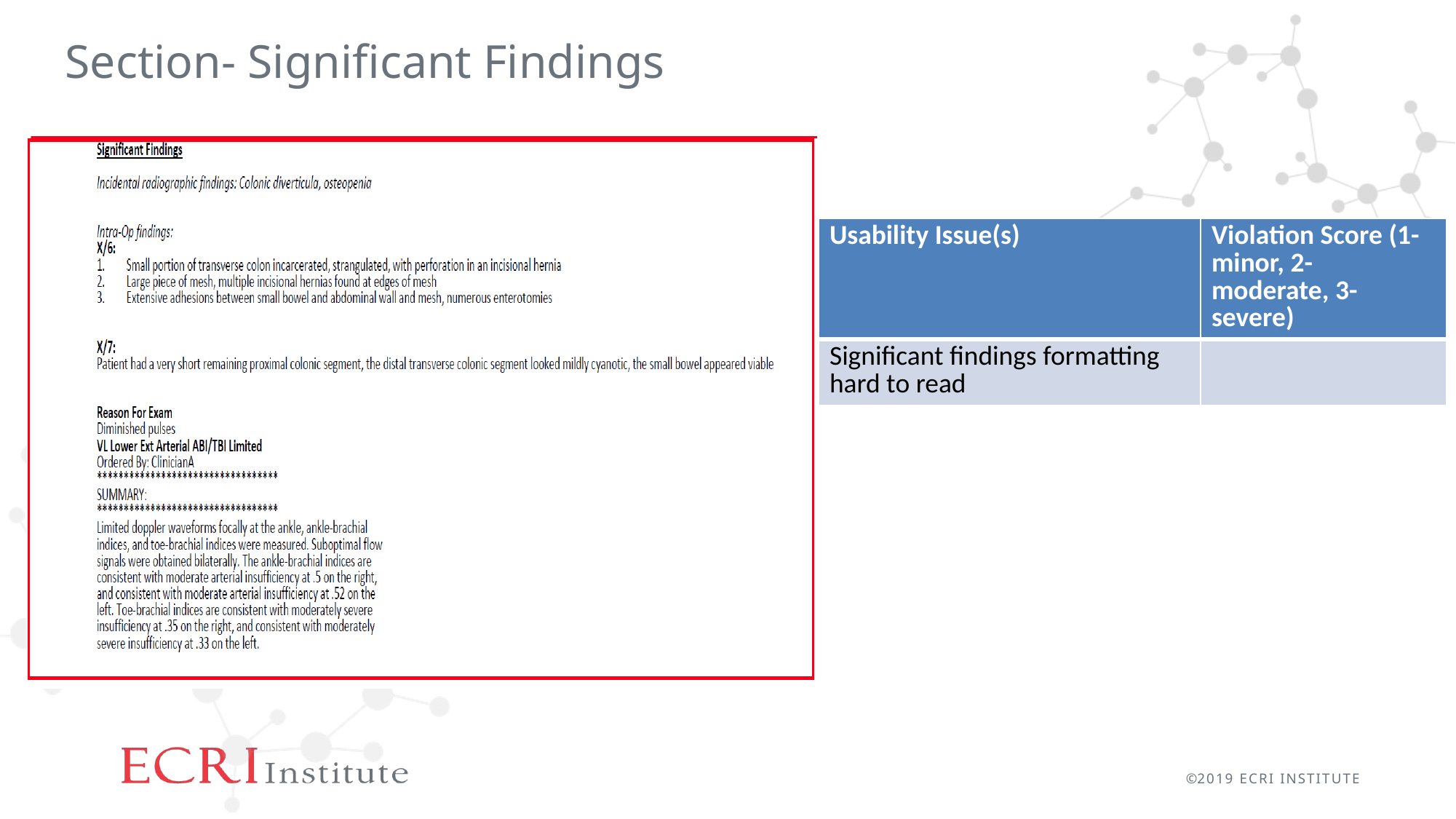

# Section- Significant Findings
| Usability Issue(s) | Violation Score (1- minor, 2- moderate, 3- severe) |
| --- | --- |
| Significant findings formatting hard to read | |

## Slide 9
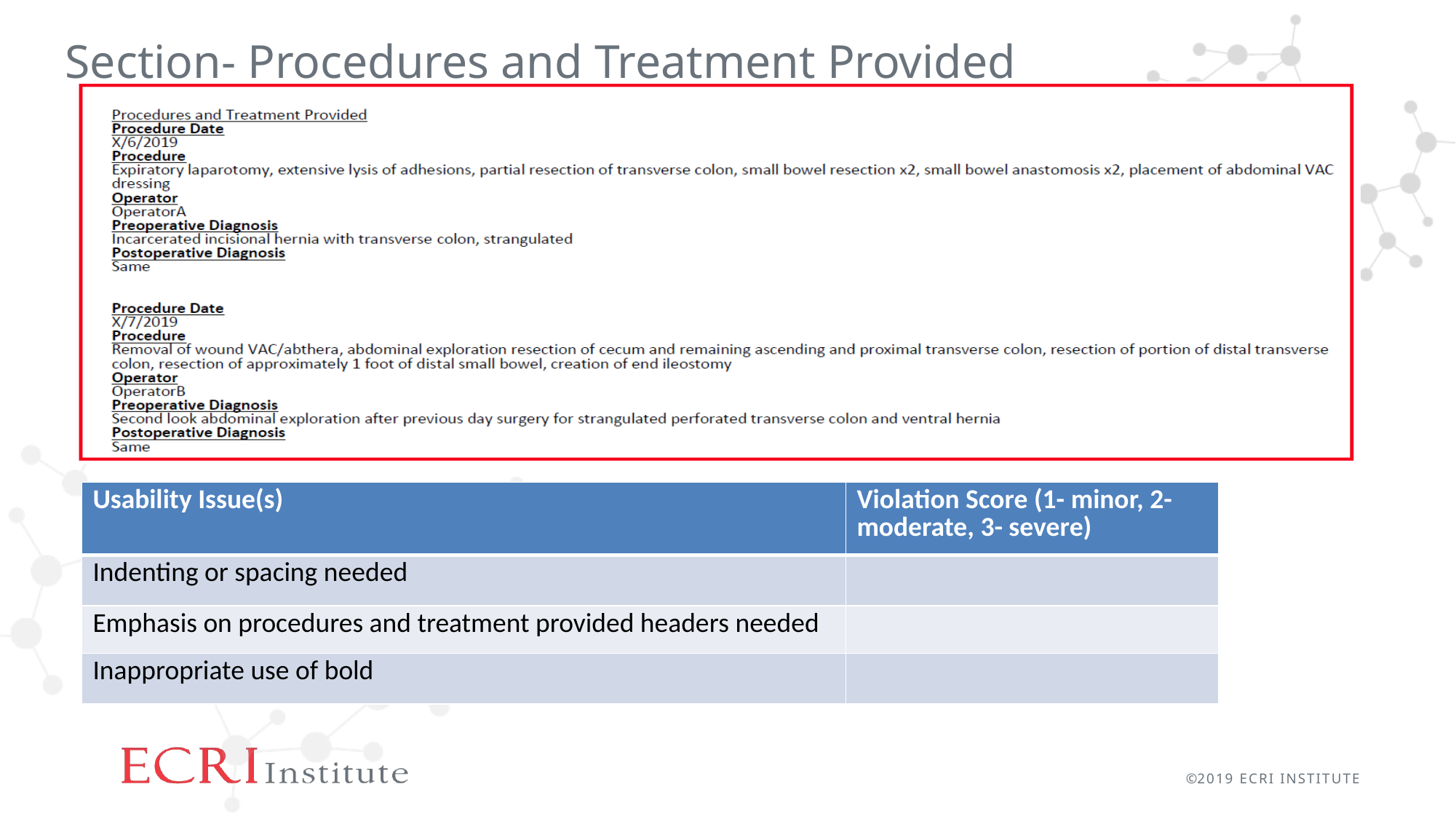

# Section- Procedures and Treatment Provided
| Usability Issue(s) | Violation Score (1- minor, 2- moderate, 3- severe) |
| --- | --- |
| Indenting or spacing needed | |
| Emphasis on procedures and treatment provided headers needed | |
| Inappropriate use of bold | |

## Slide 10
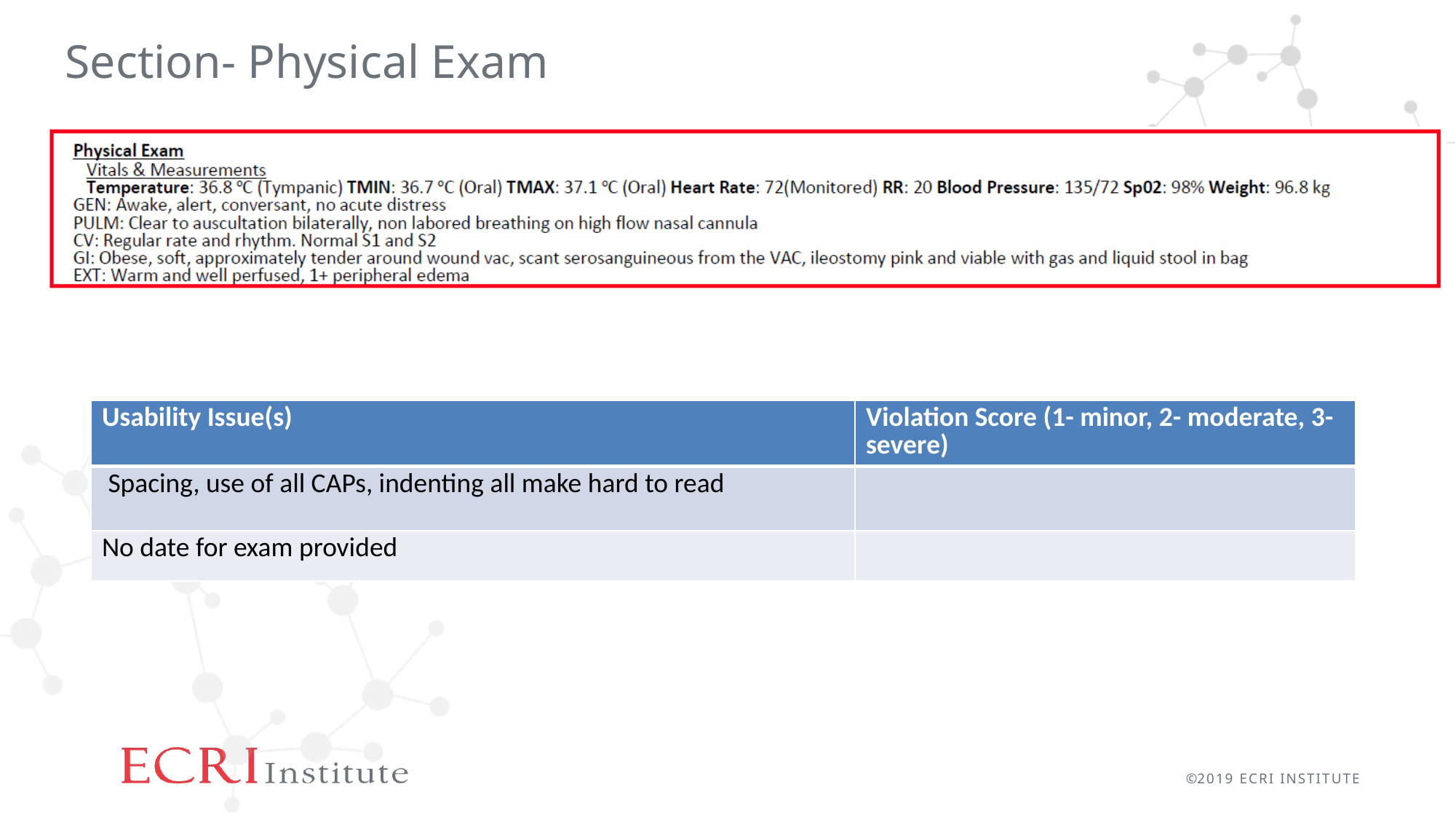

# Section- Physical Exam
| Usability Issue(s) | Violation Score (1- minor, 2- moderate, 3- severe) |
| --- | --- |
| Spacing, use of all CAPs, indenting all make hard to read | |
| No date for exam provided | |

## Slide 11
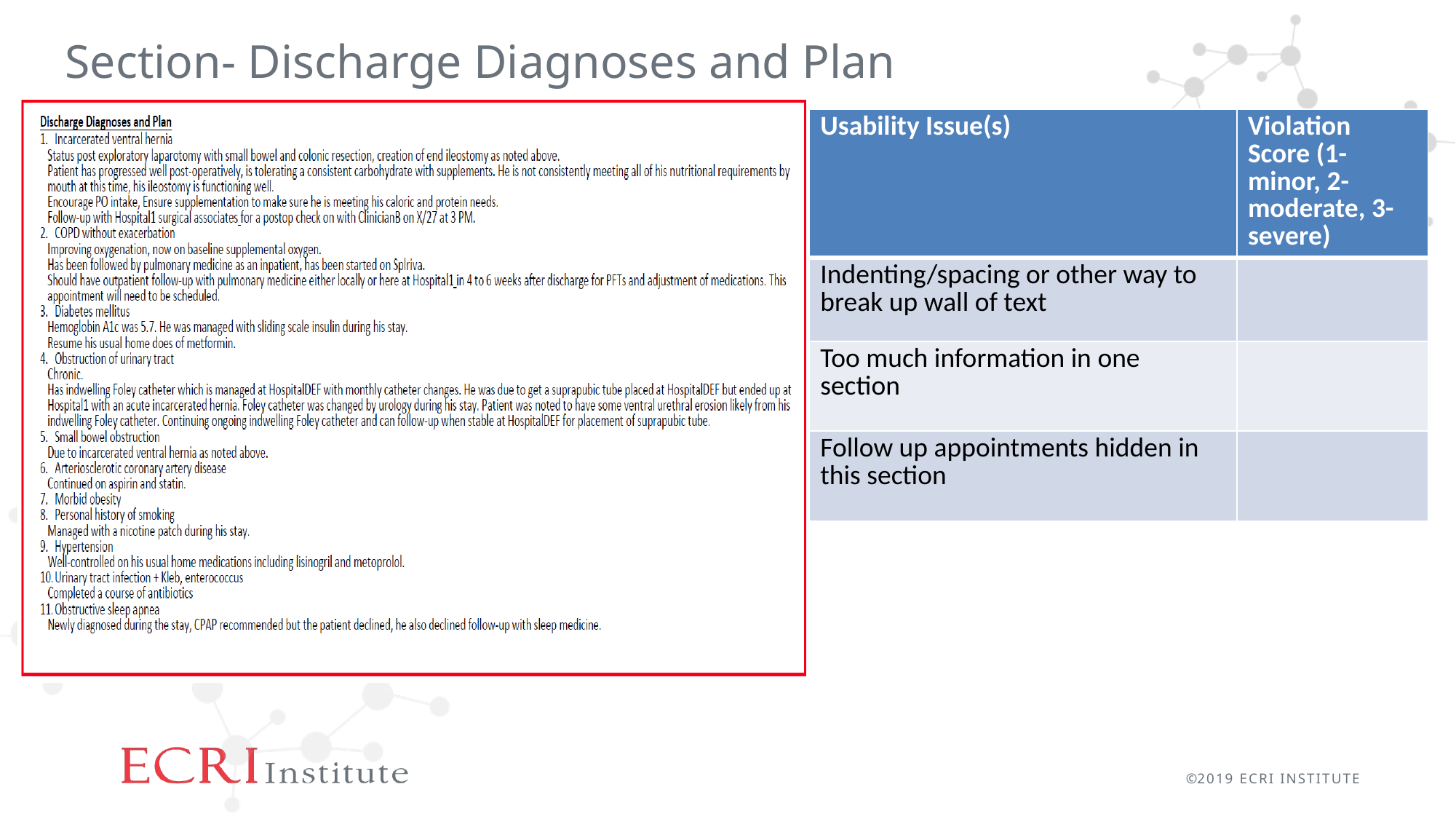

# Section- Discharge Diagnoses and Plan
| Usability Issue(s) | Violation Score (1- minor, 2- moderate, 3- severe) |
| --- | --- |
| Indenting/spacing or other way to break up wall of text | |
| Too much information in one section | |
| Follow up appointments hidden in this section | |

## Slide 12
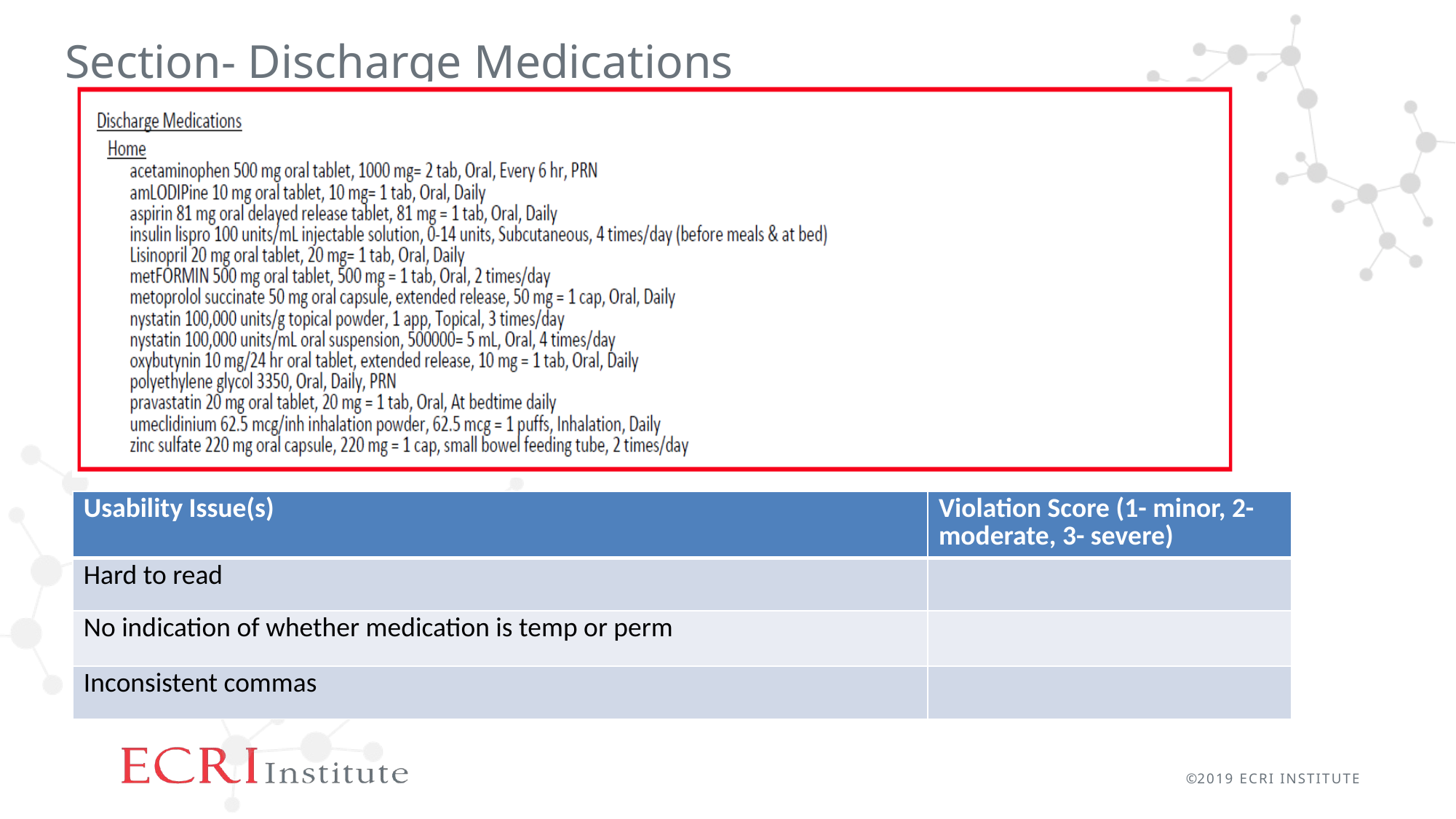

# Section- Discharge Medications
| Usability Issue(s) | Violation Score (1- minor, 2- moderate, 3- severe) |
| --- | --- |
| Hard to read | |
| No indication of whether medication is temp or perm | |
| Inconsistent commas | |

## Slide 13
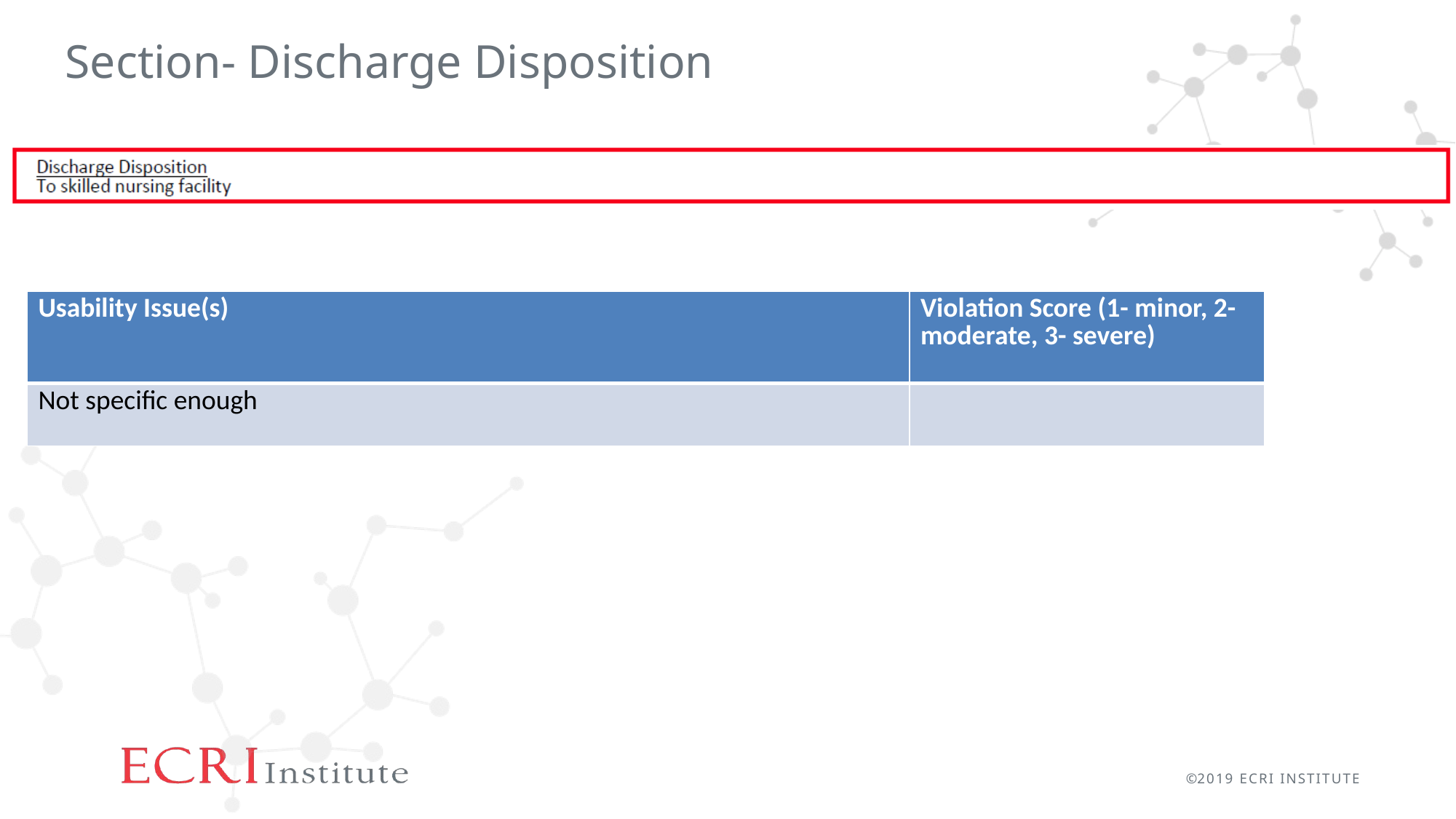

# Section- Discharge Disposition
| Usability Issue(s) | Violation Score (1- minor, 2- moderate, 3- severe) |
| --- | --- |
| Not specific enough | |

## Slide 14
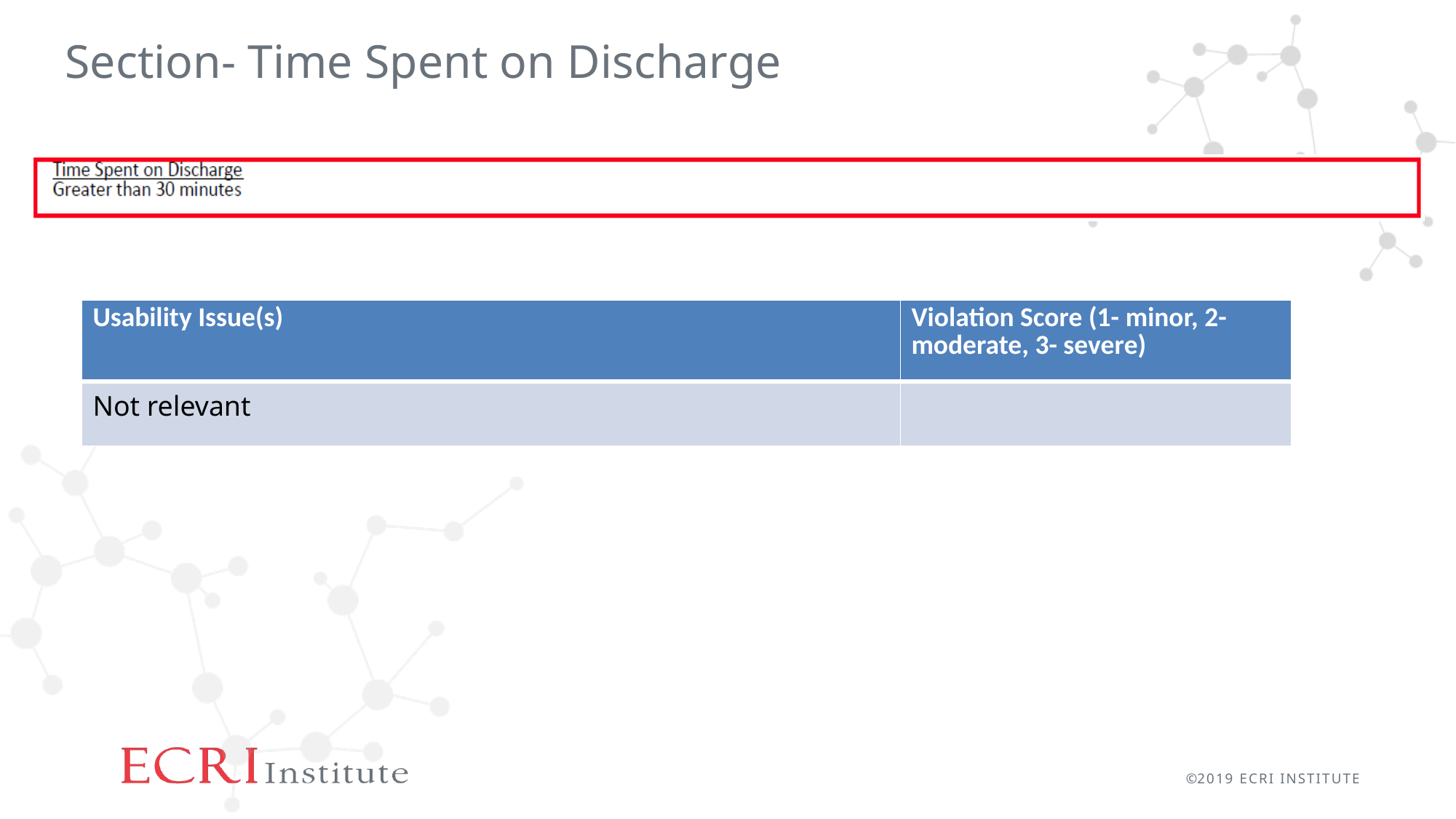

# Section- Time Spent on Discharge
| Usability Issue(s) | Violation Score (1- minor, 2- moderate, 3- severe) |
| --- | --- |
| Not relevant | |

## Slide 15
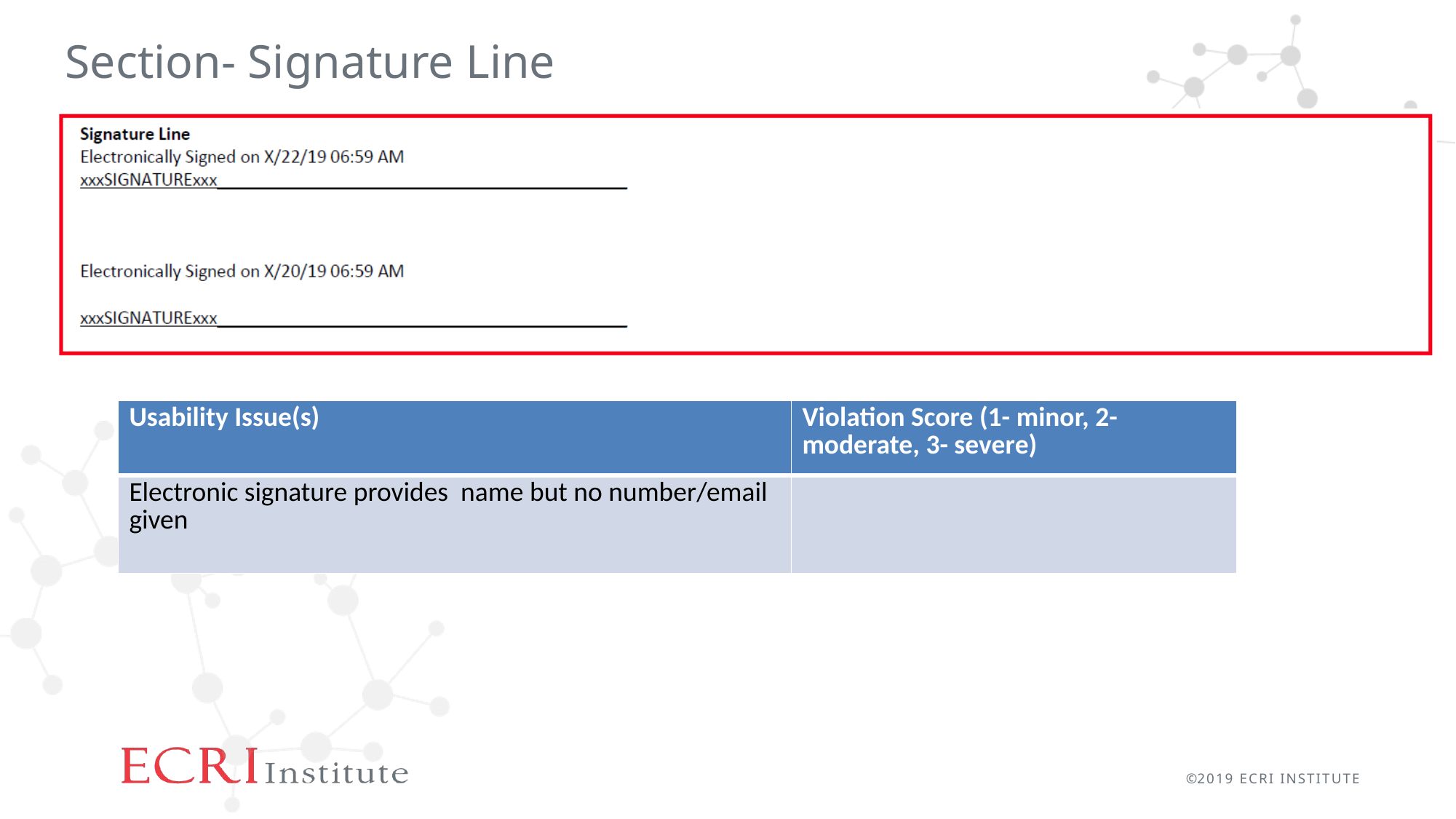

# Section- Signature Line
| Usability Issue(s) | Violation Score (1- minor, 2- moderate, 3- severe) |
| --- | --- |
| Electronic signature provides name but no number/email given | |

## Slide 16
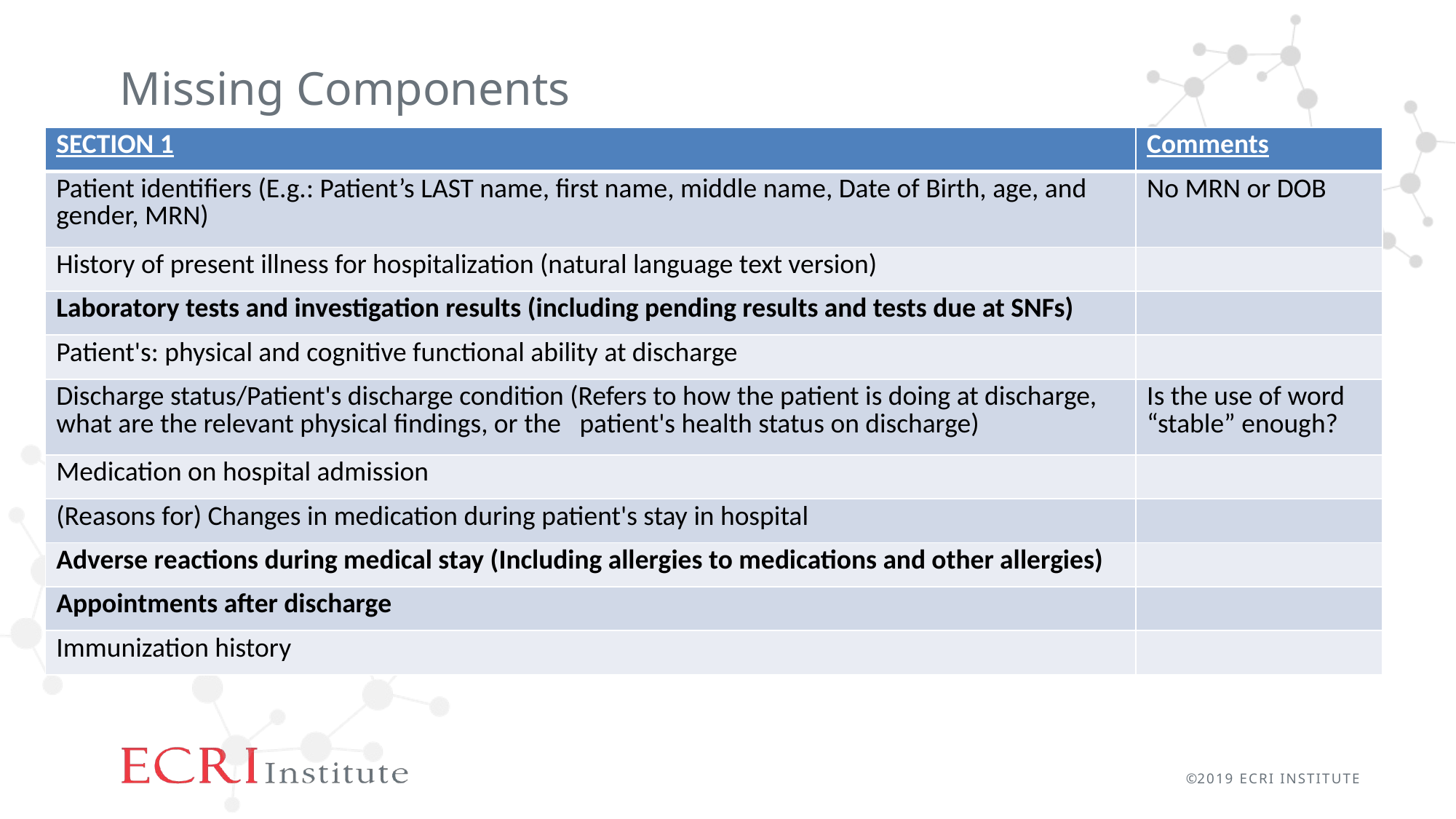

# Missing Components
| SECTION 1 | Comments |
| --- | --- |
| Patient identifiers (E.g.: Patient’s LAST name, first name, middle name, Date of Birth, age, and gender, MRN) | No MRN or DOB |
| History of present illness for hospitalization (natural language text version) | |
| Laboratory tests and investigation results (including pending results and tests due at SNFs) | |
| Patient's: physical and cognitive functional ability at discharge | |
| Discharge status/Patient's discharge condition (Refers to how the patient is doing at discharge, what are the relevant physical findings, or the patient's health status on discharge) | Is the use of word “stable” enough? |
| Medication on hospital admission | |
| (Reasons for) Changes in medication during patient's stay in hospital | |
| Adverse reactions during medical stay (Including allergies to medications and other allergies) | |
| Appointments after discharge | |
| Immunization history | |

## Slide 17
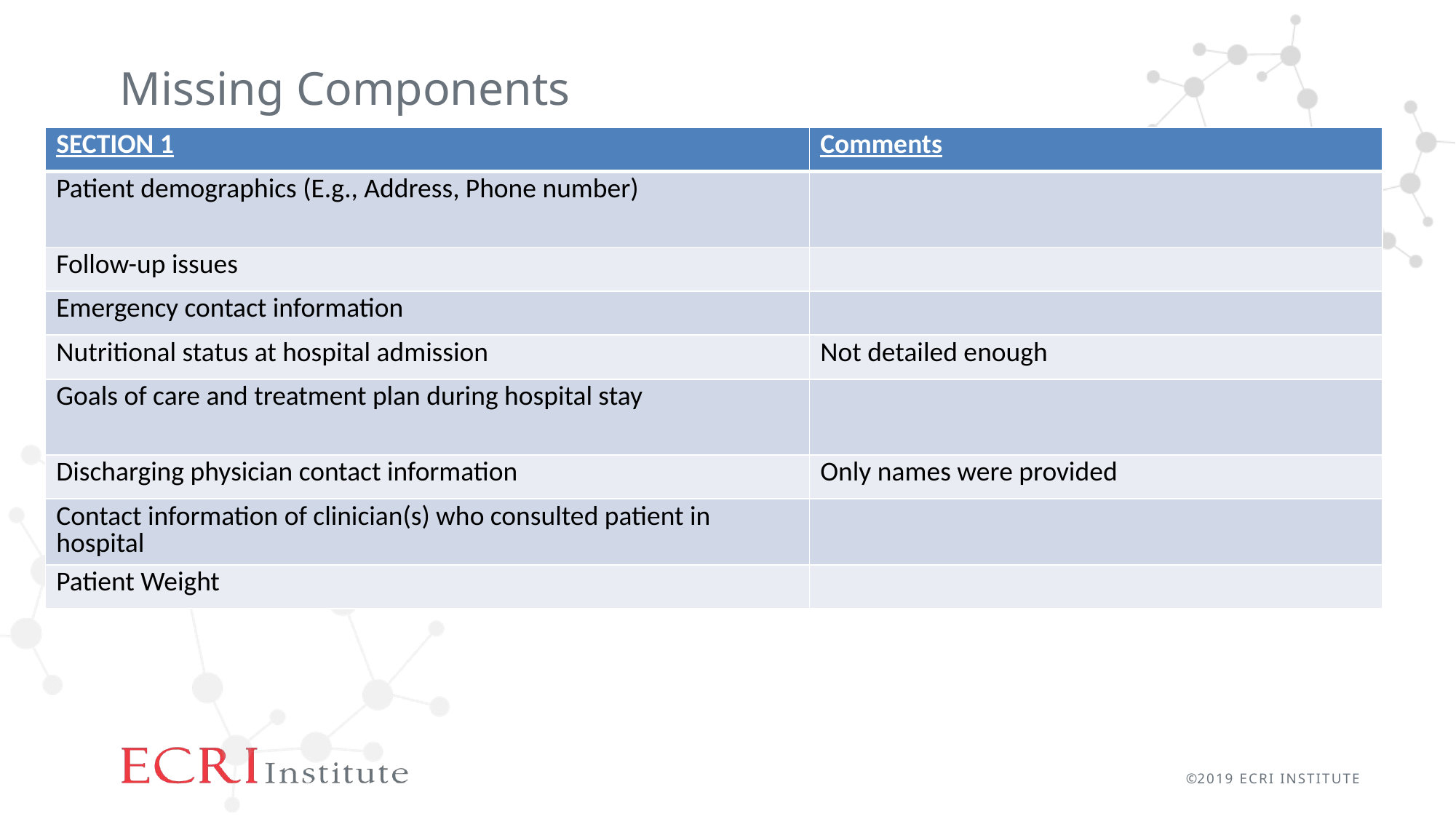

# Missing Components
| SECTION 1 | Comments |
| --- | --- |
| Patient demographics (E.g., Address, Phone number) | |
| Follow-up issues | |
| Emergency contact information | |
| Nutritional status at hospital admission | Not detailed enough |
| Goals of care and treatment plan during hospital stay | |
| Discharging physician contact information | Only names were provided |
| Contact information of clinician(s) who consulted patient in hospital | |
| Patient Weight | |

## Slide 18
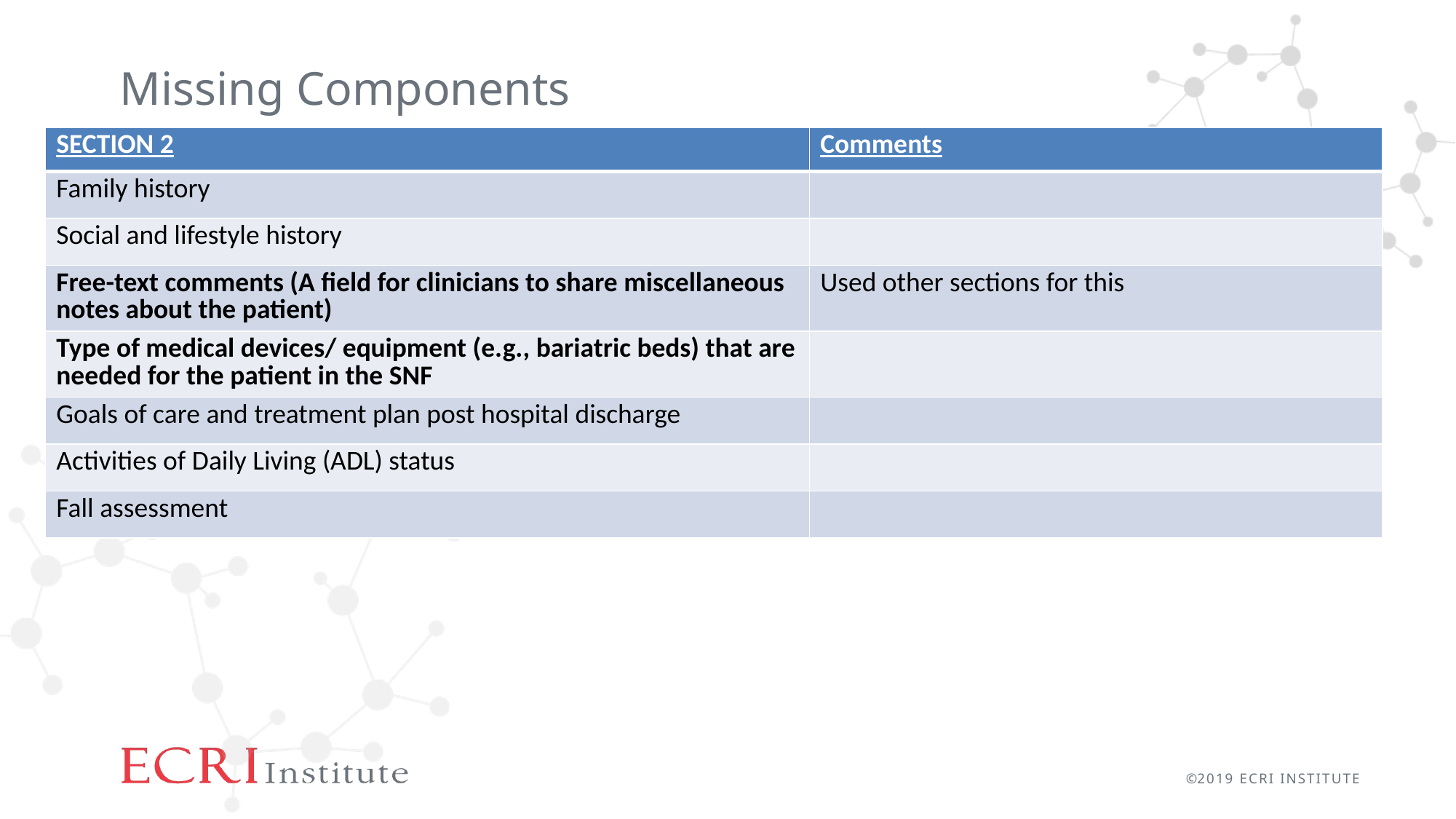

# Missing Components
| SECTION 2 | Comments |
| --- | --- |
| Family history | |
| Social and lifestyle history | |
| Free-text comments (A field for clinicians to share miscellaneous notes about the patient) | Used other sections for this |
| Type of medical devices/ equipment (e.g., bariatric beds) that are needed for the patient in the SNF | |
| Goals of care and treatment plan post hospital discharge | |
| Activities of Daily Living (ADL) status | |
| Fall assessment | |

## Slide 19
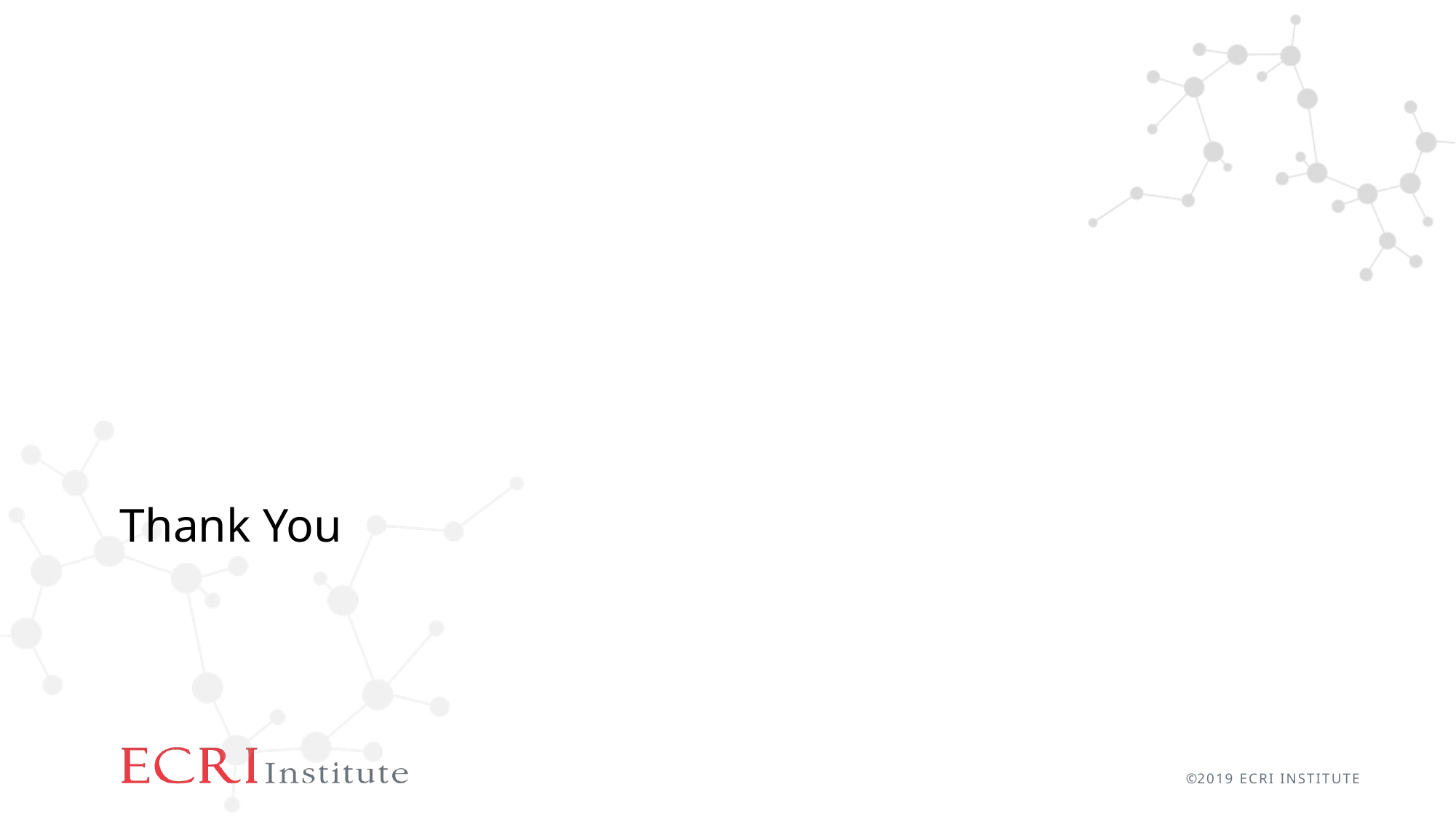

#
