# Supplementary material for: Usability of Electronic Health Record–Generated Discharge Summaries: Heuristic Evaluation
Source: J Med Internet Res. 2021 Apr 15;23(4):e25657. doi: 10.2196/25657 (PMC8085750; doi:10.2196/25657)
Supplement: Multimedia Appendix 10 [file jmir_v23i4e25657_app10.pptx]

## Slide 1
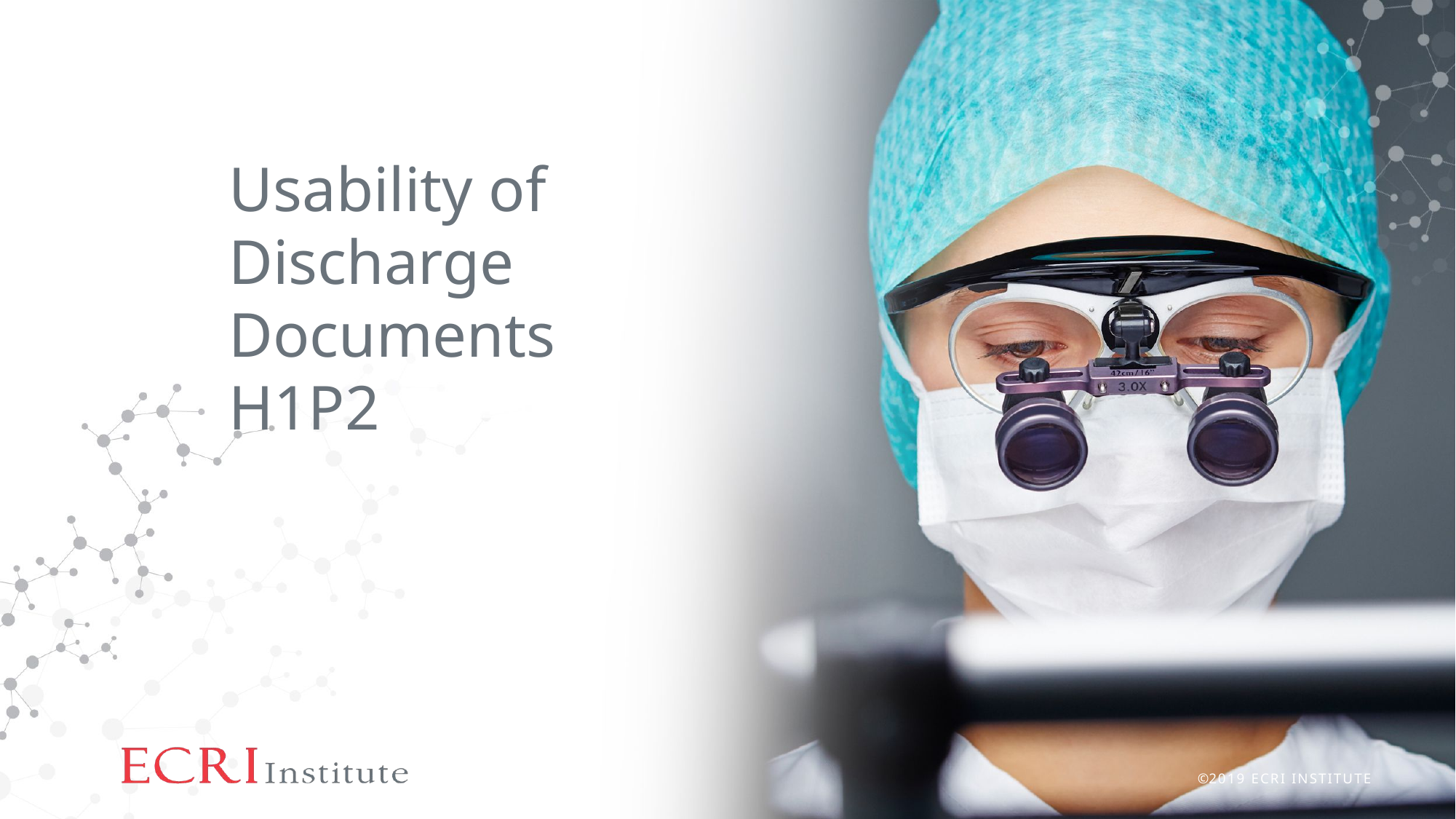

# Usability of Discharge Documents H1P2

## Slide 2
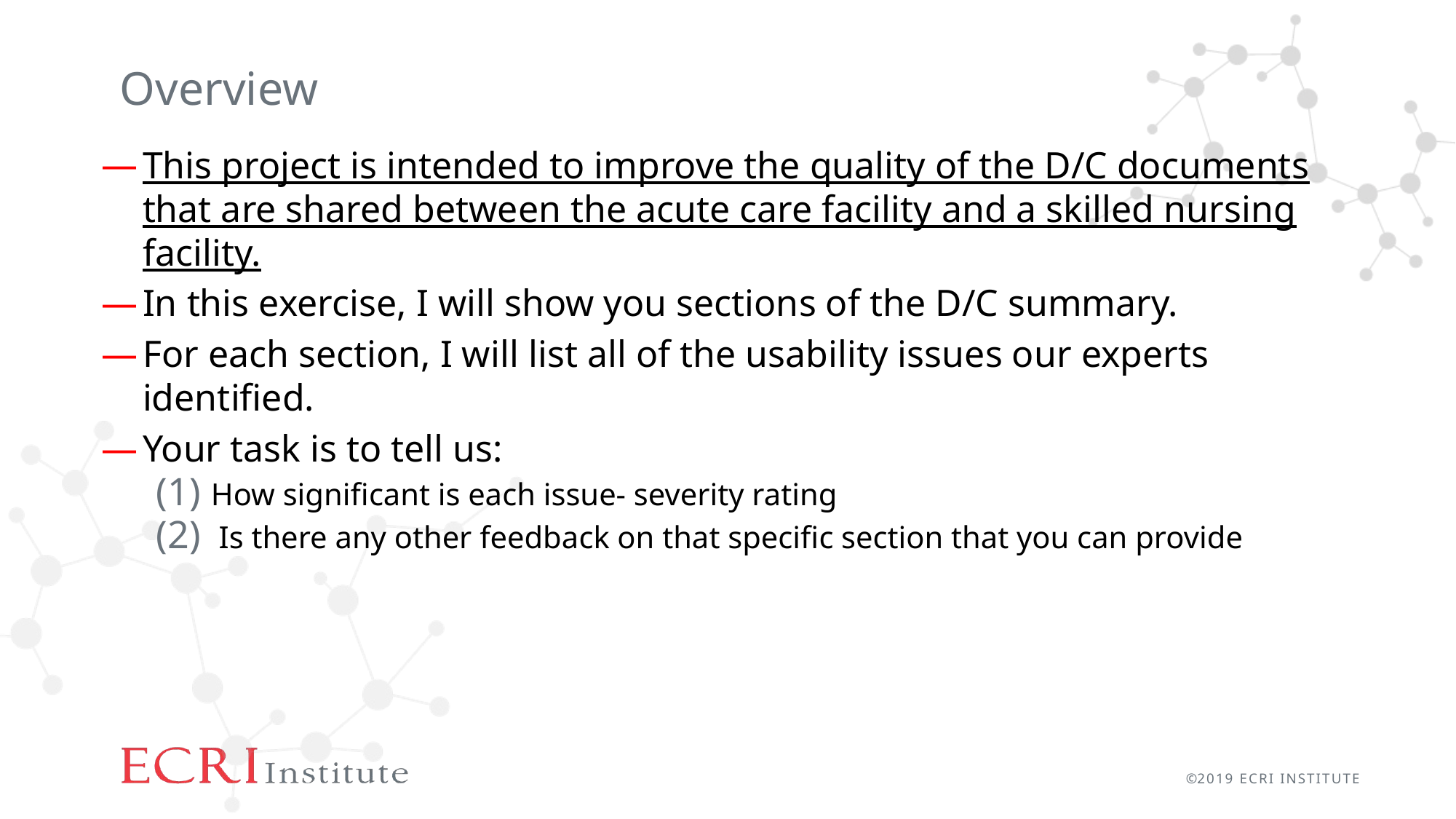

# Overview
This project is intended to improve the quality of the D/C documents that are shared between the acute care facility and a skilled nursing facility.
In this exercise, I will show you sections of the D/C summary.
For each section, I will list all of the usability issues our experts identified.
Your task is to tell us:
How significant is each issue- severity rating
 Is there any other feedback on that specific section that you can provide

## Slide 3
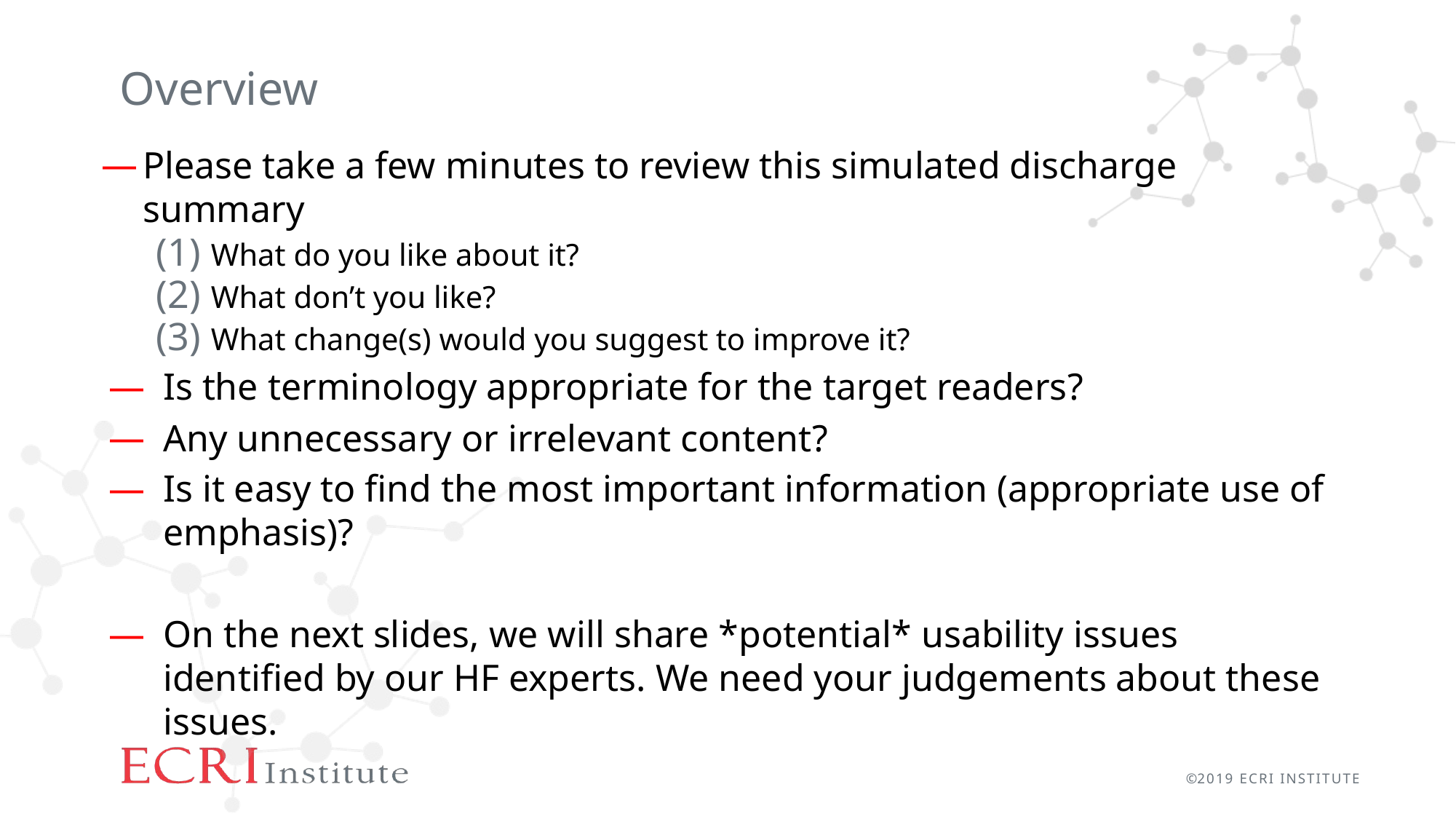

# Overview
Please take a few minutes to review this simulated discharge summary
What do you like about it?
What don’t you like?
What change(s) would you suggest to improve it?
Is the terminology appropriate for the target readers?
Any unnecessary or irrelevant content?
Is it easy to find the most important information (appropriate use of emphasis)?
On the next slides, we will share *potential* usability issues identified by our HF experts. We need your judgements about these issues.

## Slide 4
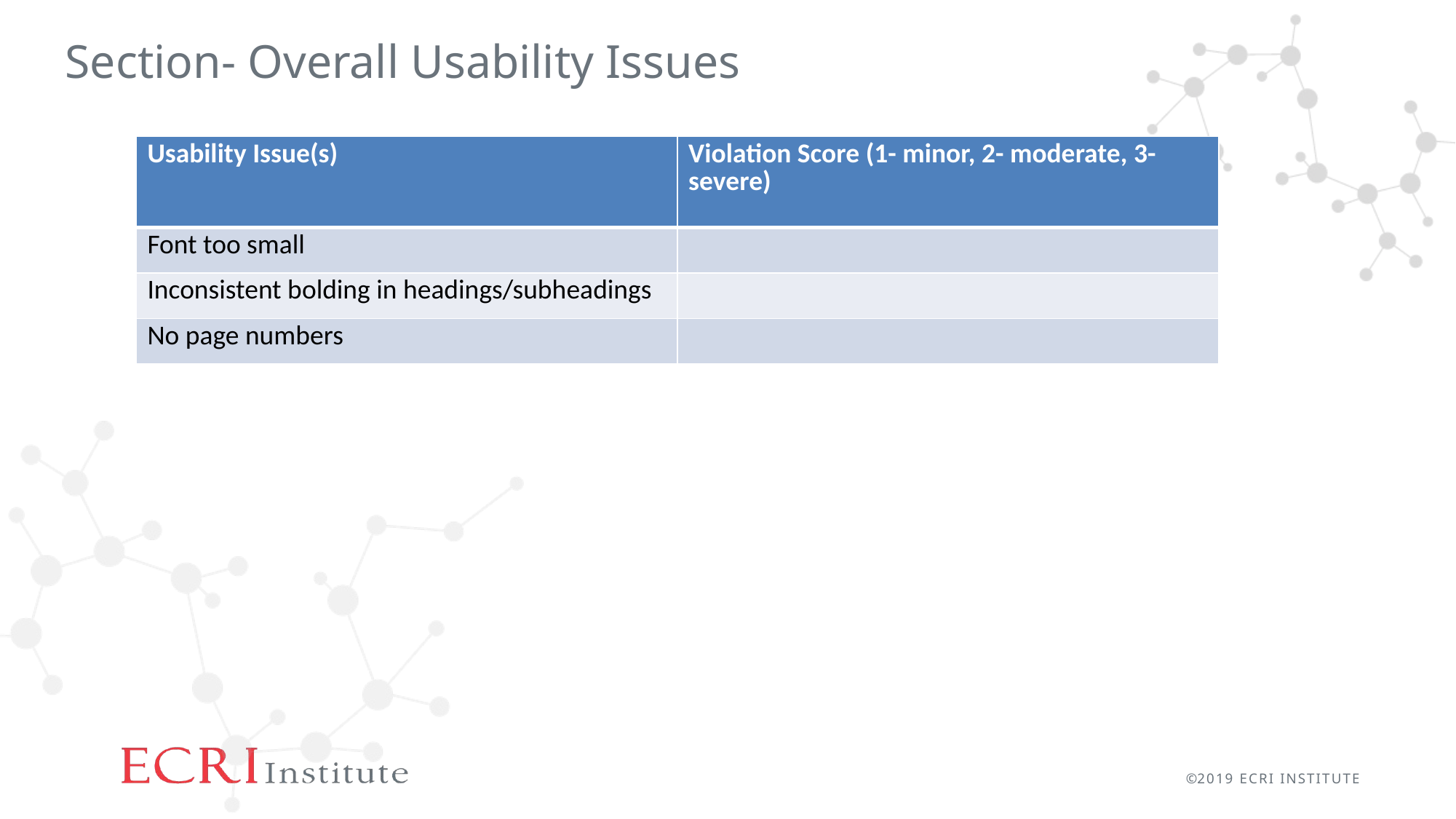

# Section- Overall Usability Issues
| Usability Issue(s) | Violation Score (1- minor, 2- moderate, 3- severe) |
| --- | --- |
| Font too small | |
| Inconsistent bolding in headings/subheadings | |
| No page numbers | |

## Slide 5
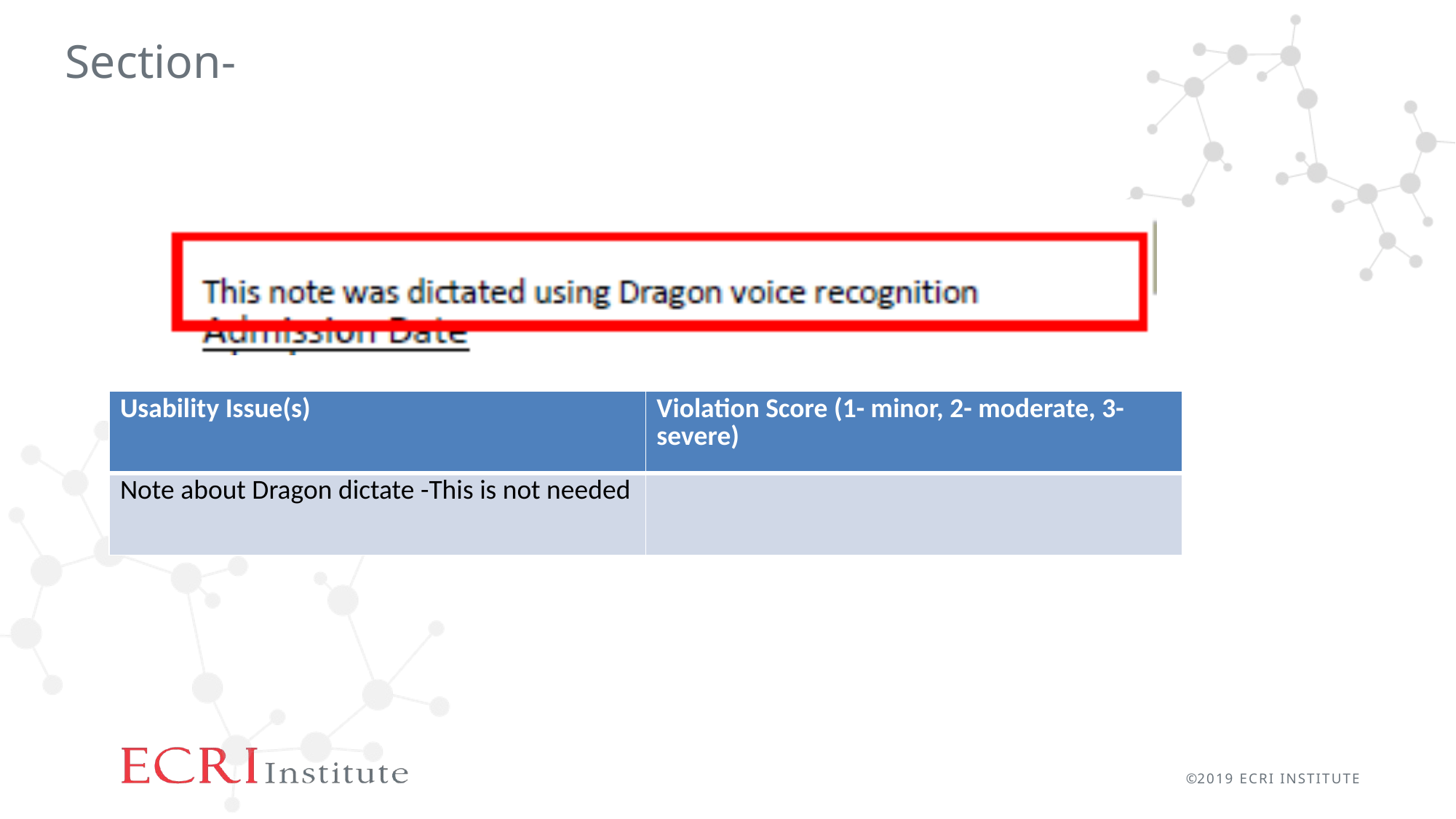

# Section-
| Usability Issue(s) | Violation Score (1- minor, 2- moderate, 3- severe) |
| --- | --- |
| Note about Dragon dictate -This is not needed | |

## Slide 6
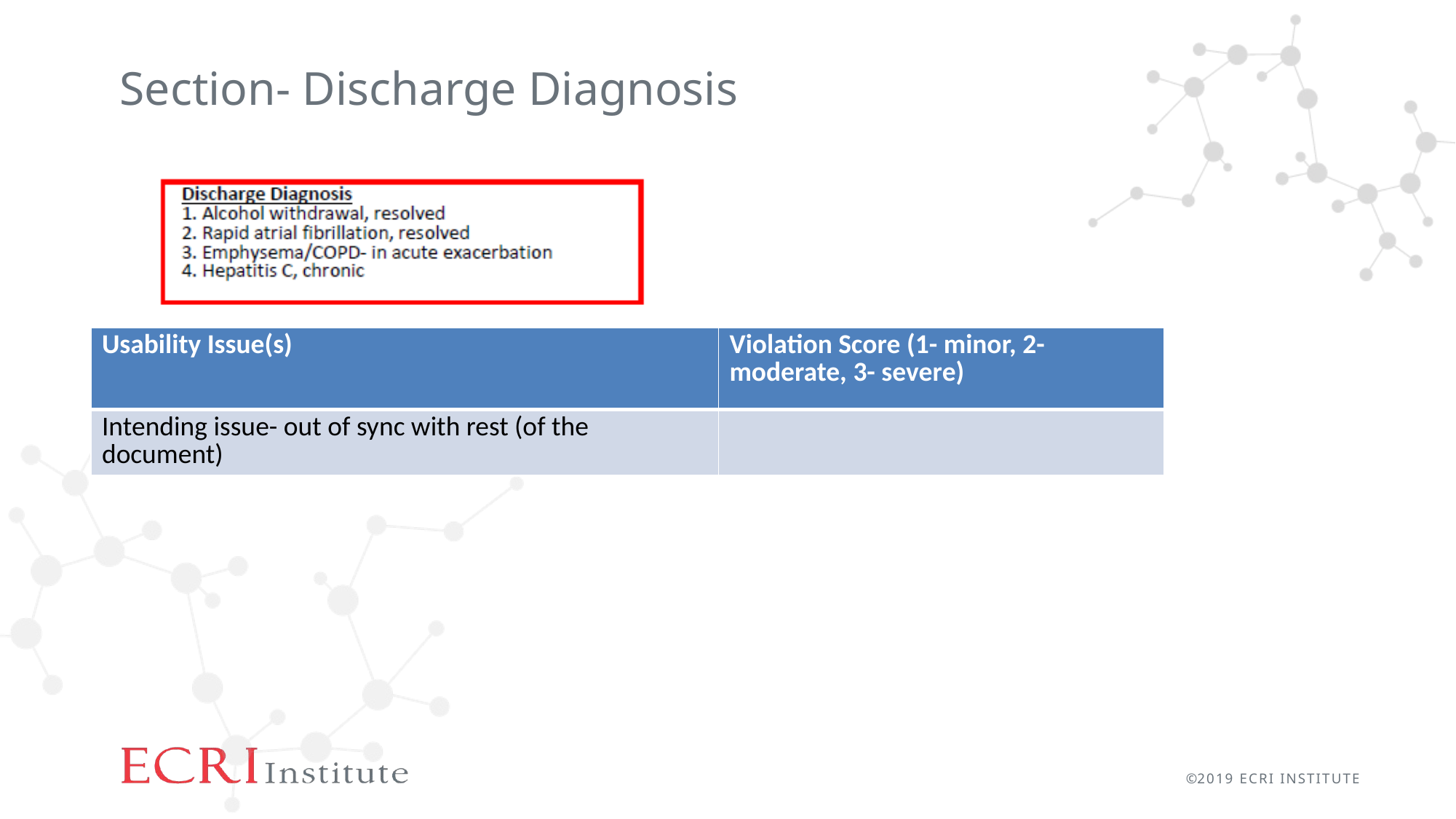

# Section- Discharge Diagnosis
| Usability Issue(s) | Violation Score (1- minor, 2- moderate, 3- severe) |
| --- | --- |
| Intending issue- out of sync with rest (of the document) | |

## Slide 7
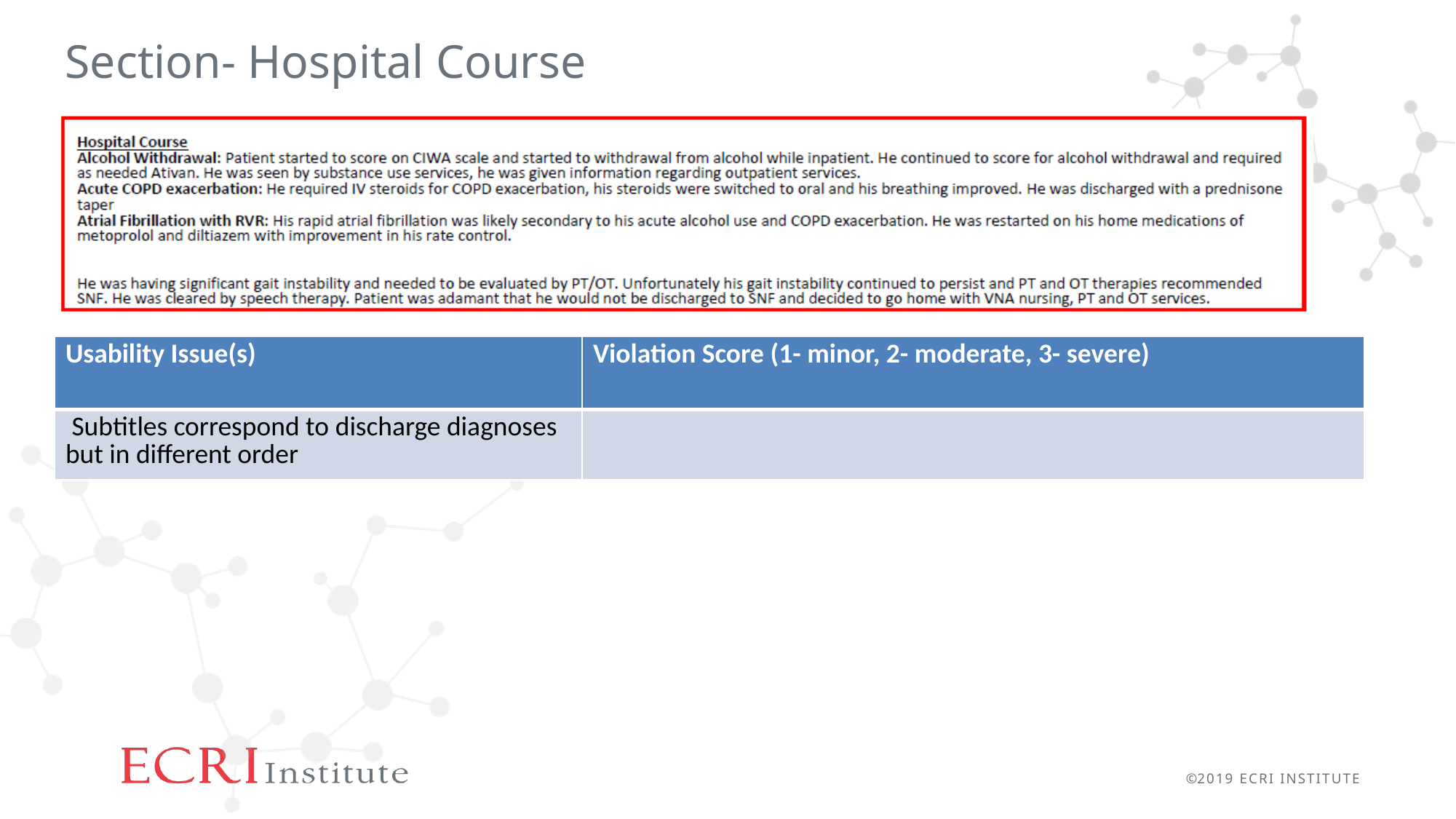

# Section- Hospital Course
| Usability Issue(s) | Violation Score (1- minor, 2- moderate, 3- severe) |
| --- | --- |
| Subtitles correspond to discharge diagnoses but in different order | |

## Slide 8
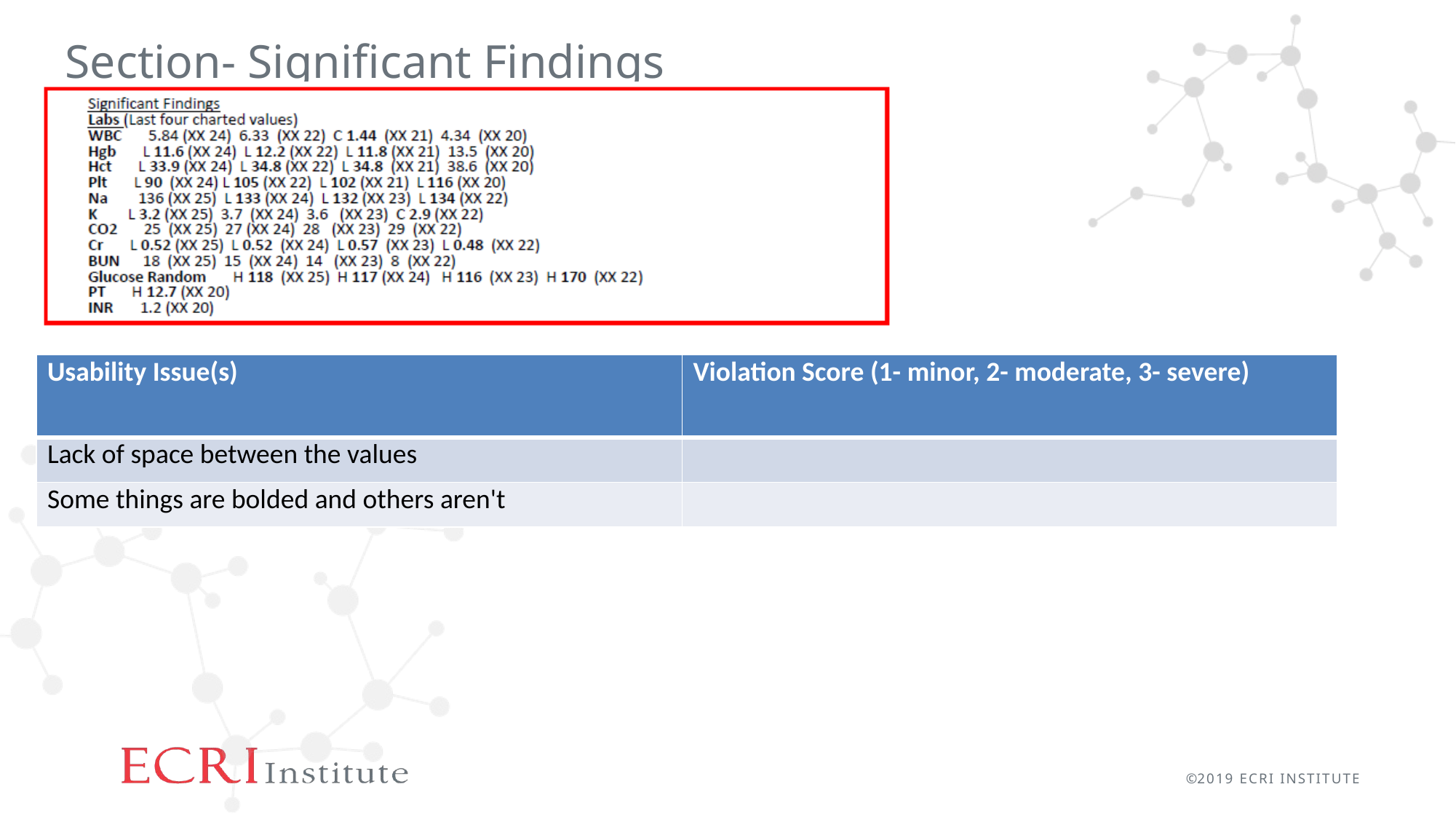

# Section- Significant Findings
| Usability Issue(s) | Violation Score (1- minor, 2- moderate, 3- severe) |
| --- | --- |
| Lack of space between the values | |
| Some things are bolded and others aren't | |

## Slide 9
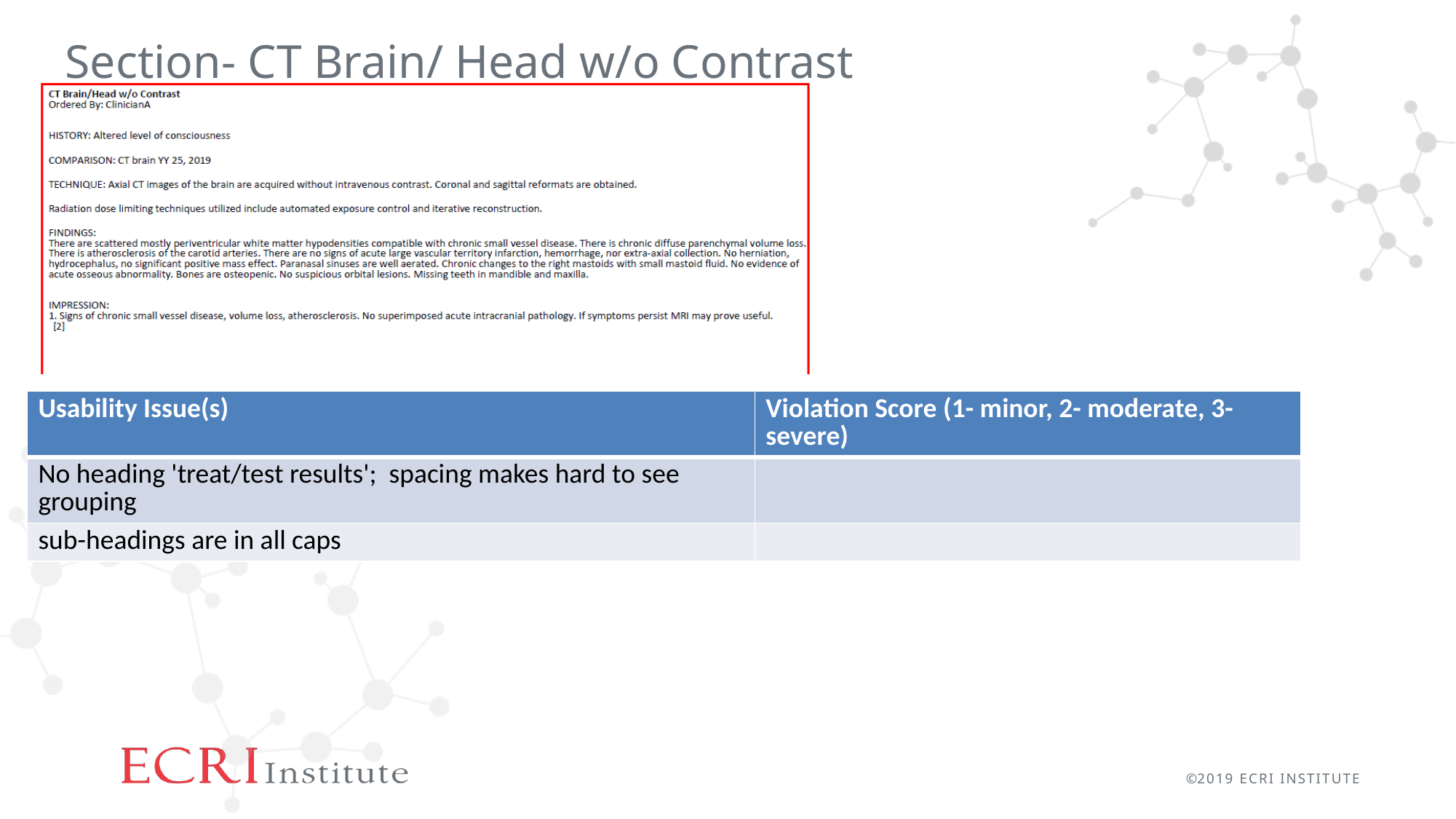

# Section- CT Brain/ Head w/o Contrast
| Usability Issue(s) | Violation Score (1- minor, 2- moderate, 3- severe) |
| --- | --- |
| No heading 'treat/test results'; spacing makes hard to see grouping | |
| sub-headings are in all caps | |

## Slide 10
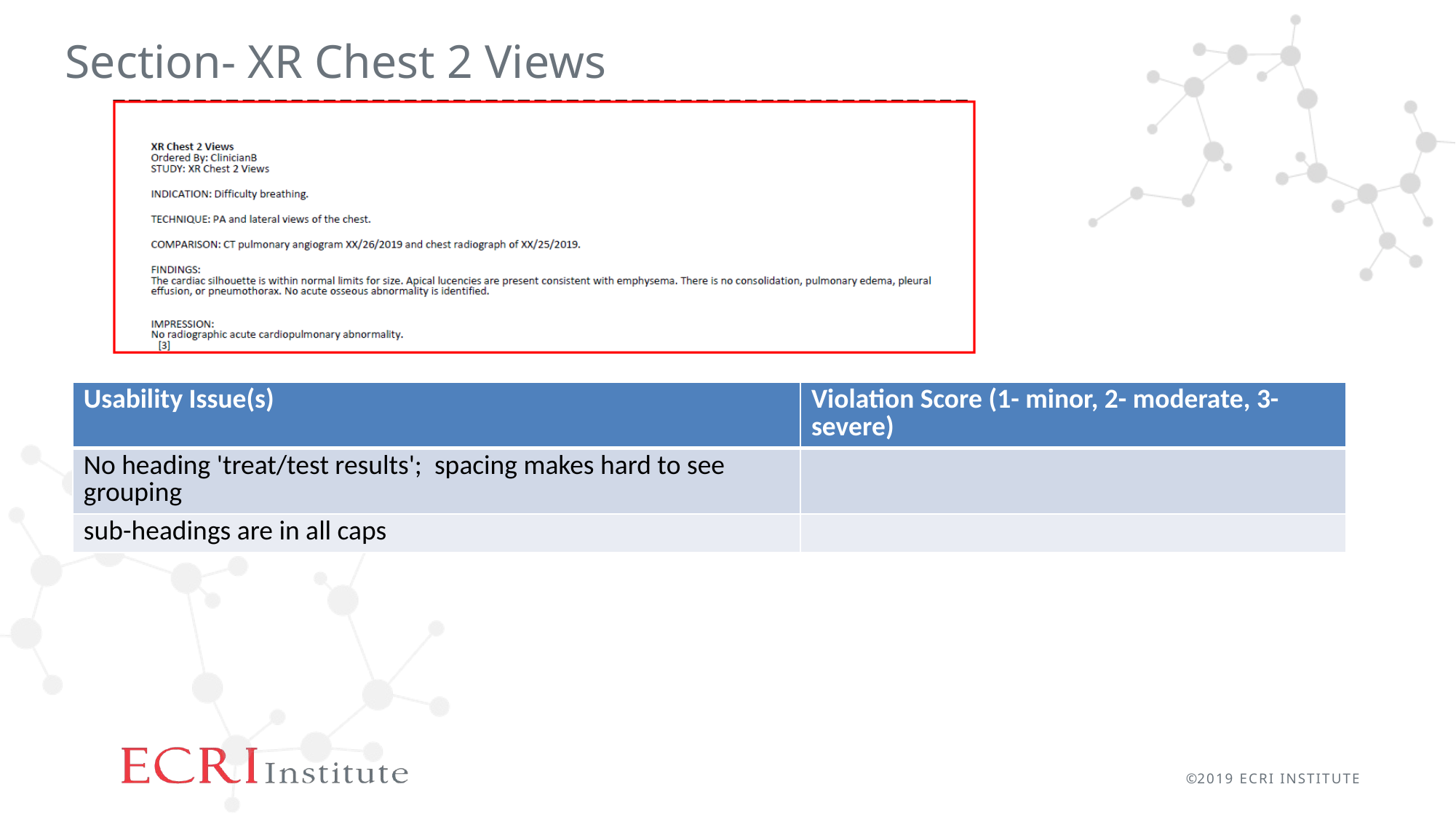

# Section- XR Chest 2 Views
| Usability Issue(s) | Violation Score (1- minor, 2- moderate, 3- severe) |
| --- | --- |
| No heading 'treat/test results'; spacing makes hard to see grouping | |
| sub-headings are in all caps | |

## Slide 11
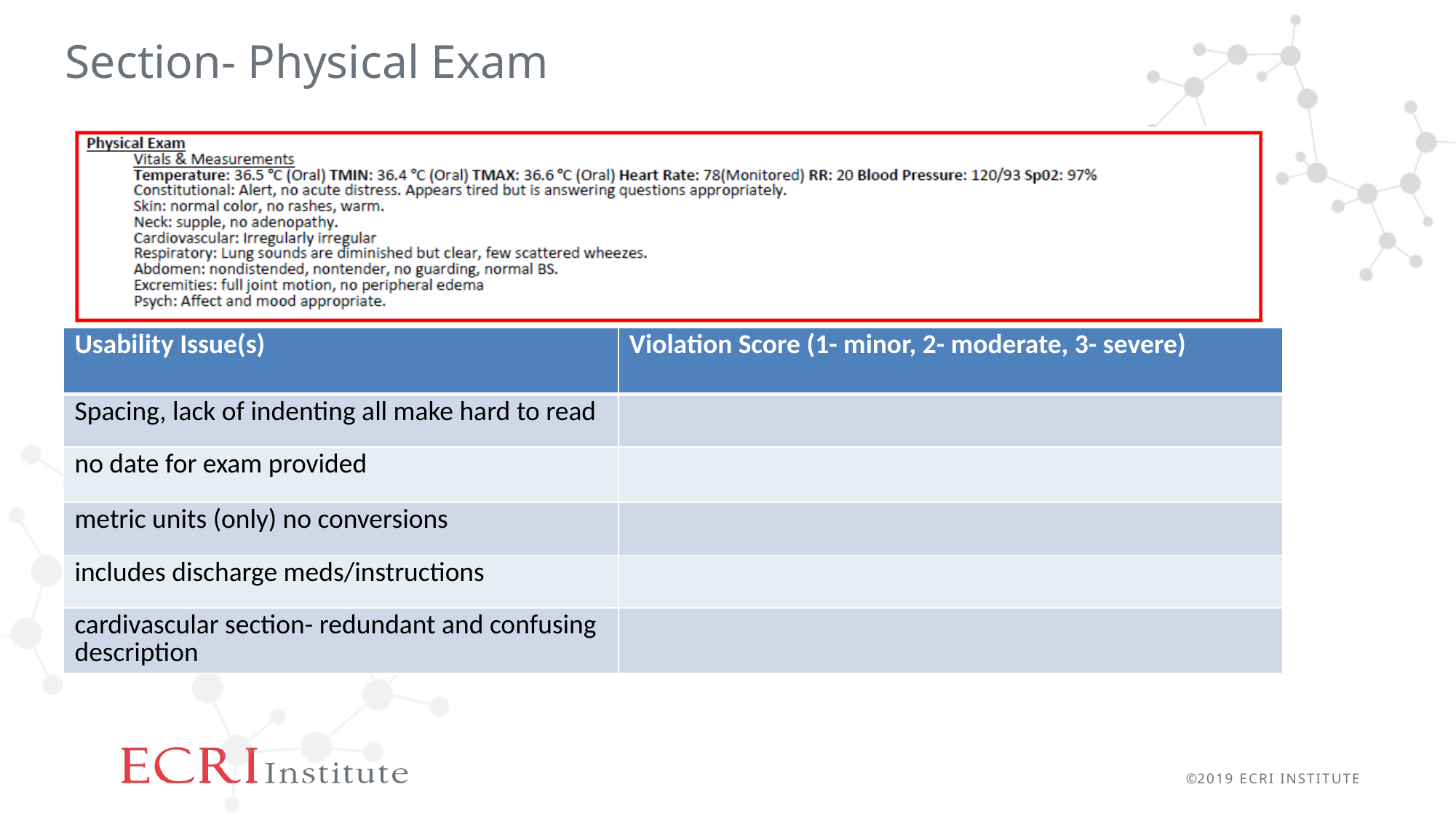

# Section- Physical Exam
| Usability Issue(s) | Violation Score (1- minor, 2- moderate, 3- severe) |
| --- | --- |
| Spacing, lack of indenting all make hard to read | |
| no date for exam provided | |
| metric units (only) no conversions | |
| includes discharge meds/instructions | |
| cardivascular section- redundant and confusing description | |

## Slide 12
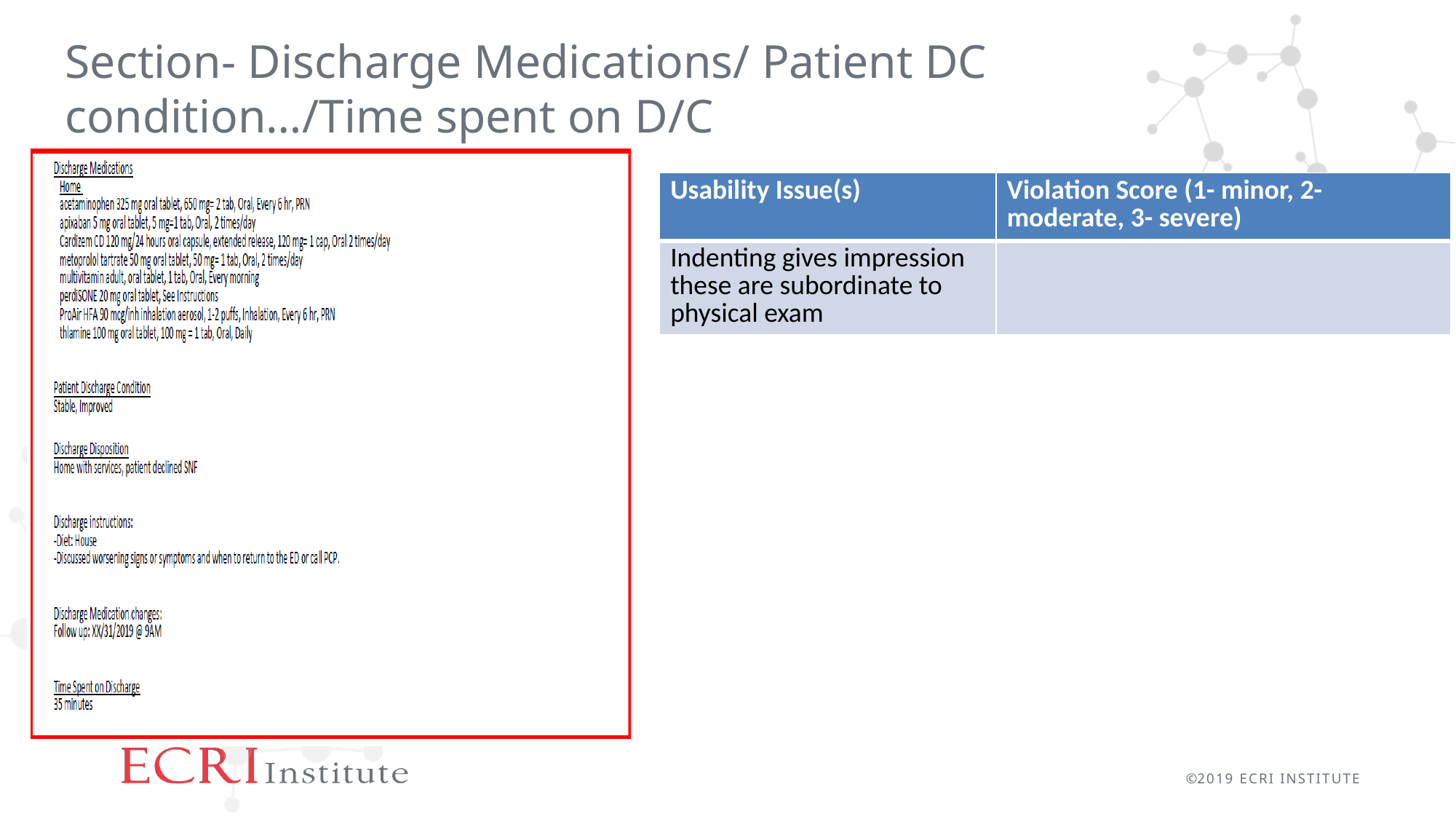

# Section- Discharge Medications/ Patient DC condition…/Time spent on D/C
| Usability Issue(s) | Violation Score (1- minor, 2- moderate, 3- severe) |
| --- | --- |
| Indenting gives impression these are subordinate to physical exam | |

## Slide 13
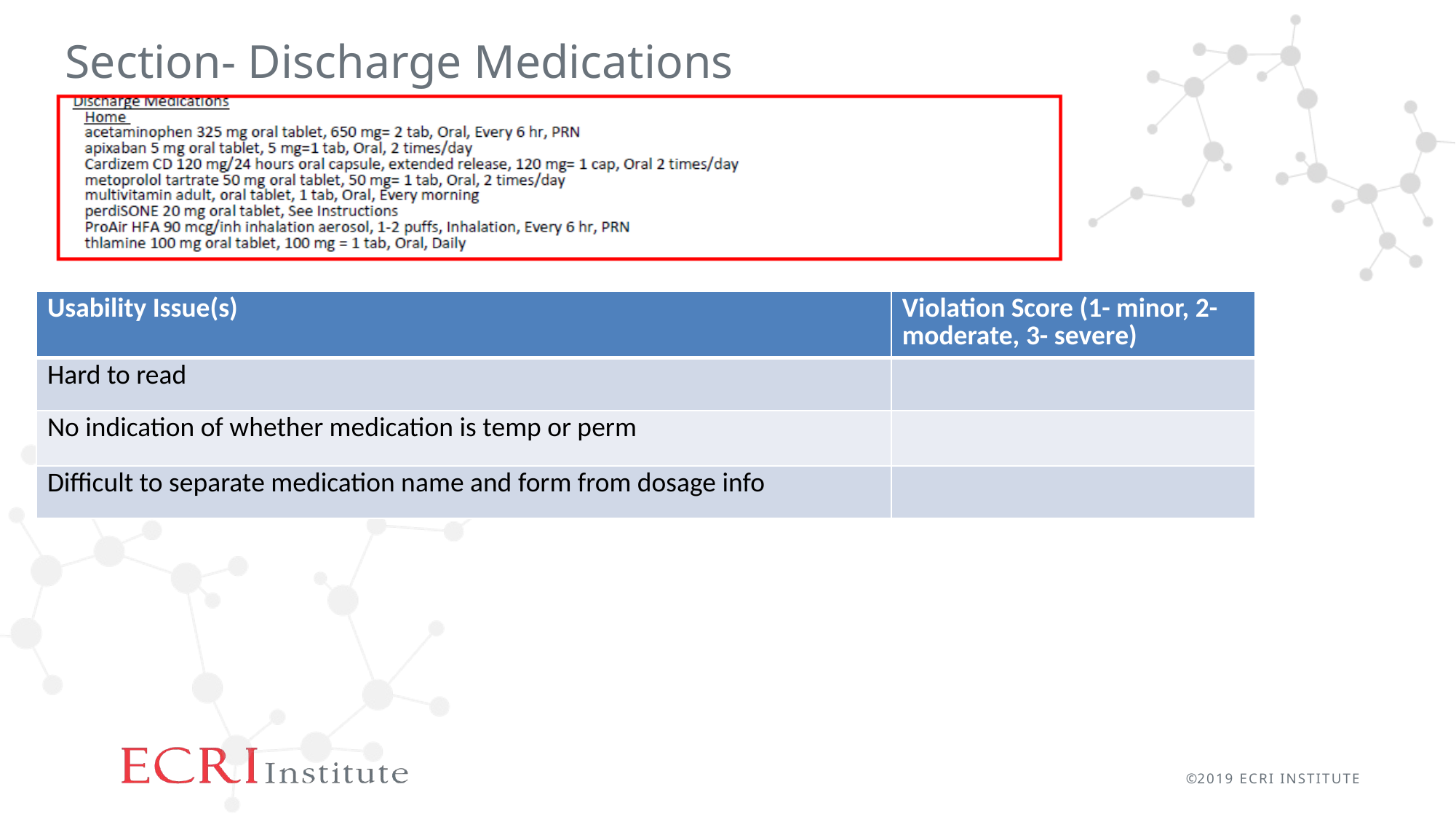

# Section- Discharge Medications
| Usability Issue(s) | Violation Score (1- minor, 2- moderate, 3- severe) |
| --- | --- |
| Hard to read | |
| No indication of whether medication is temp or perm | |
| Difficult to separate medication name and form from dosage info | |

## Slide 14
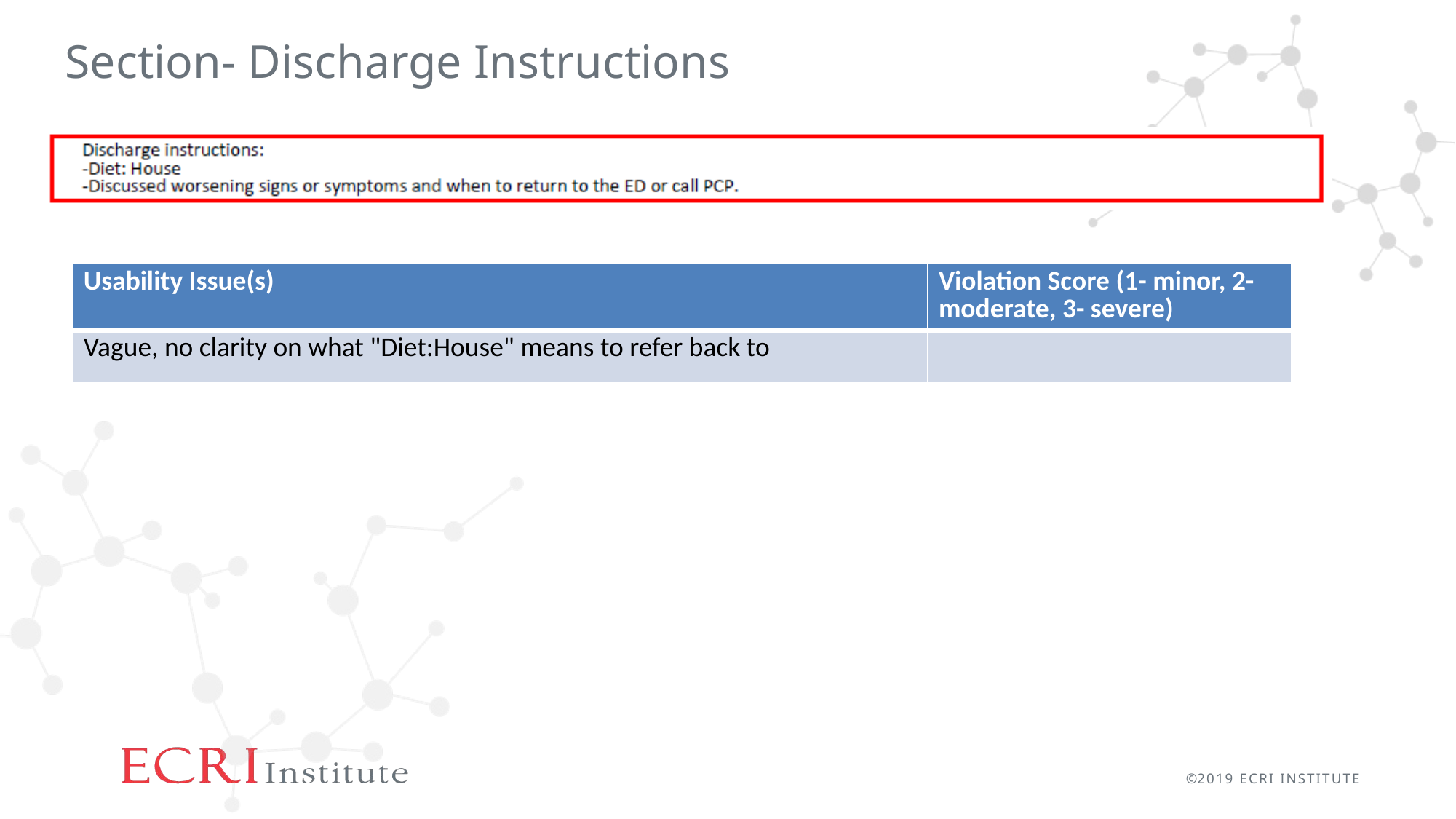

# Section- Discharge Instructions
| Usability Issue(s) | Violation Score (1- minor, 2- moderate, 3- severe) |
| --- | --- |
| Vague, no clarity on what "Diet:House" means to refer back to | |

## Slide 15
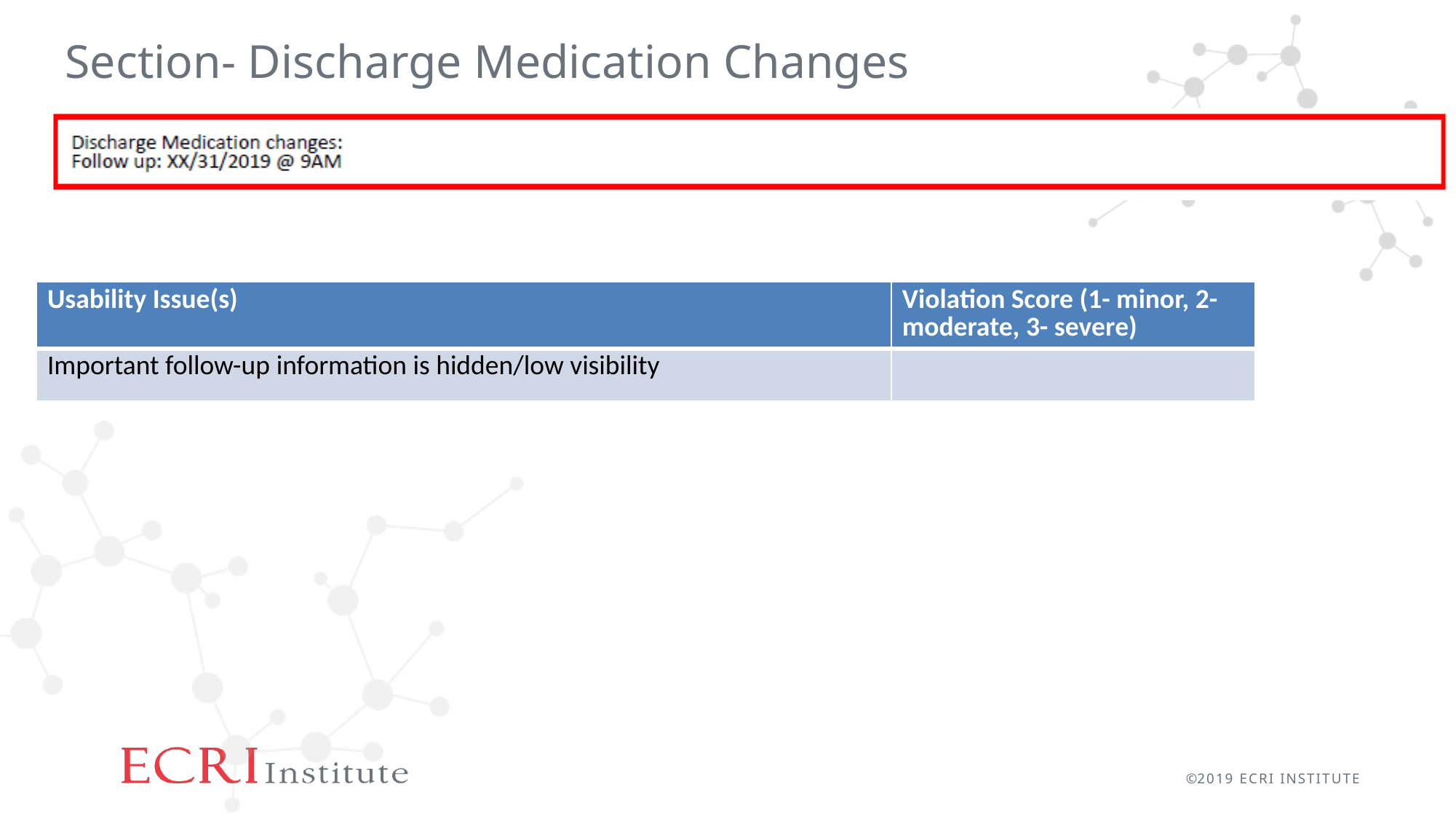

# Section- Discharge Medication Changes
| Usability Issue(s) | Violation Score (1- minor, 2- moderate, 3- severe) |
| --- | --- |
| Important follow-up information is hidden/low visibility | |

## Slide 16
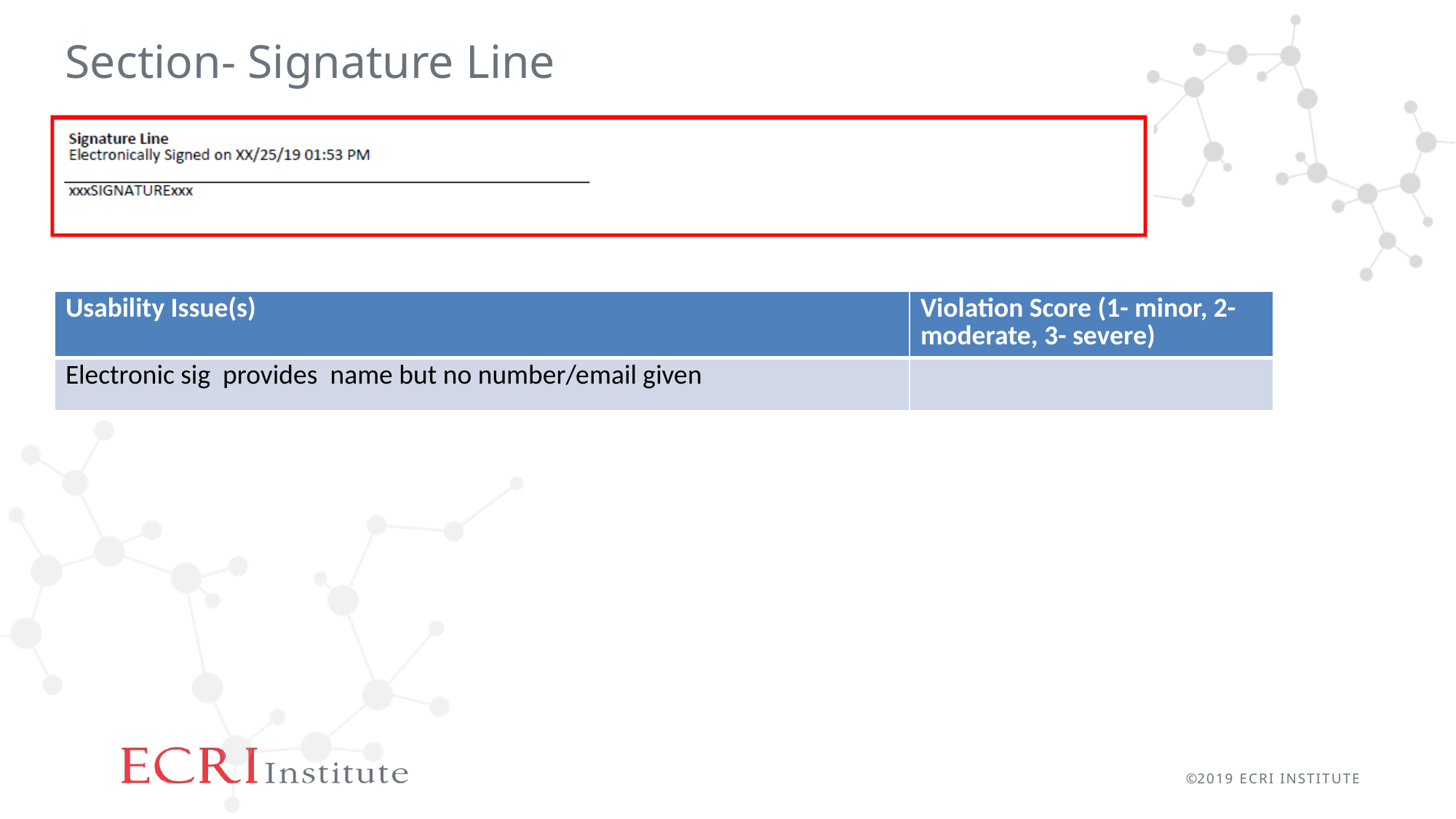

# Section- Signature Line
| Usability Issue(s) | Violation Score (1- minor, 2- moderate, 3- severe) |
| --- | --- |
| Electronic sig provides name but no number/email given | |

## Slide 17
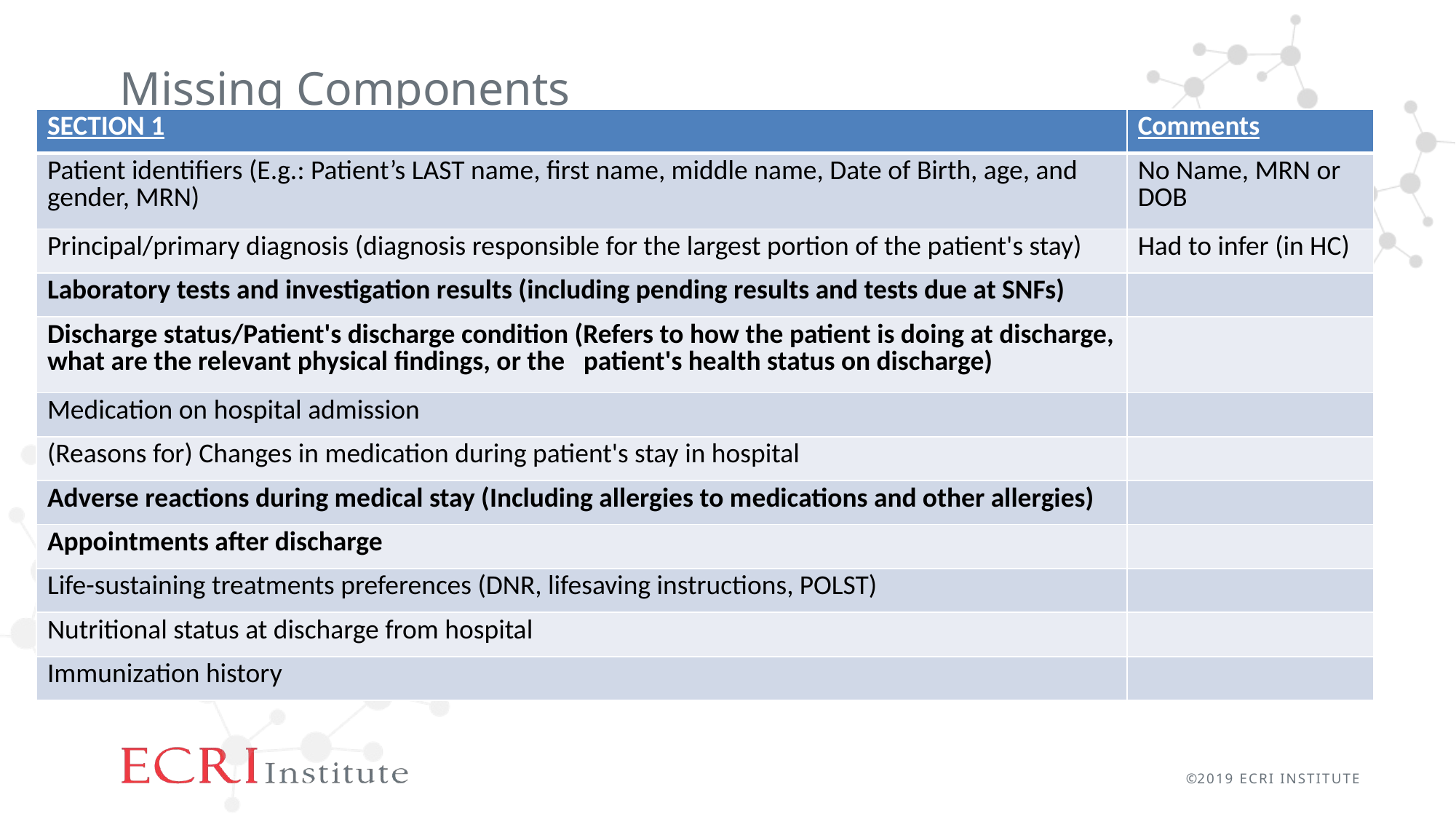

# Missing Components
| SECTION 1 | Comments |
| --- | --- |
| Patient identifiers (E.g.: Patient’s LAST name, first name, middle name, Date of Birth, age, and gender, MRN) | No Name, MRN or DOB |
| Principal/primary diagnosis (diagnosis responsible for the largest portion of the patient's stay) | Had to infer (in HC) |
| Laboratory tests and investigation results (including pending results and tests due at SNFs) | |
| Discharge status/Patient's discharge condition (Refers to how the patient is doing at discharge, what are the relevant physical findings, or the patient's health status on discharge) | |
| Medication on hospital admission | |
| (Reasons for) Changes in medication during patient's stay in hospital | |
| Adverse reactions during medical stay (Including allergies to medications and other allergies) | |
| Appointments after discharge | |
| Life-sustaining treatments preferences (DNR, lifesaving instructions, POLST) | |
| Nutritional status at discharge from hospital | |
| Immunization history | |

## Slide 18
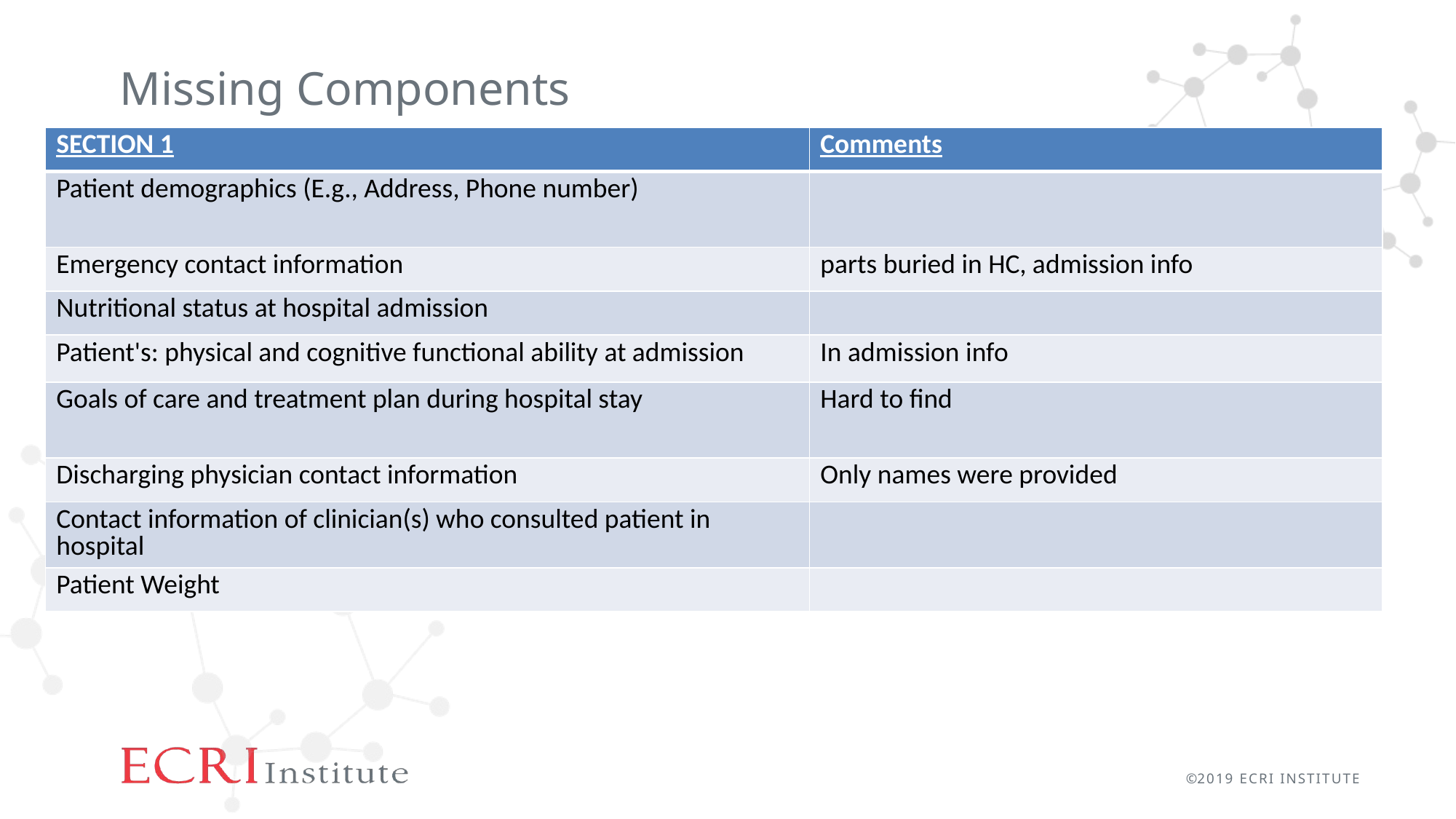

# Missing Components
| SECTION 1 | Comments |
| --- | --- |
| Patient demographics (E.g., Address, Phone number) | |
| Emergency contact information | parts buried in HC, admission info |
| Nutritional status at hospital admission | |
| Patient's: physical and cognitive functional ability at admission | In admission info |
| Goals of care and treatment plan during hospital stay | Hard to find |
| Discharging physician contact information | Only names were provided |
| Contact information of clinician(s) who consulted patient in hospital | |
| Patient Weight | |

## Slide 19
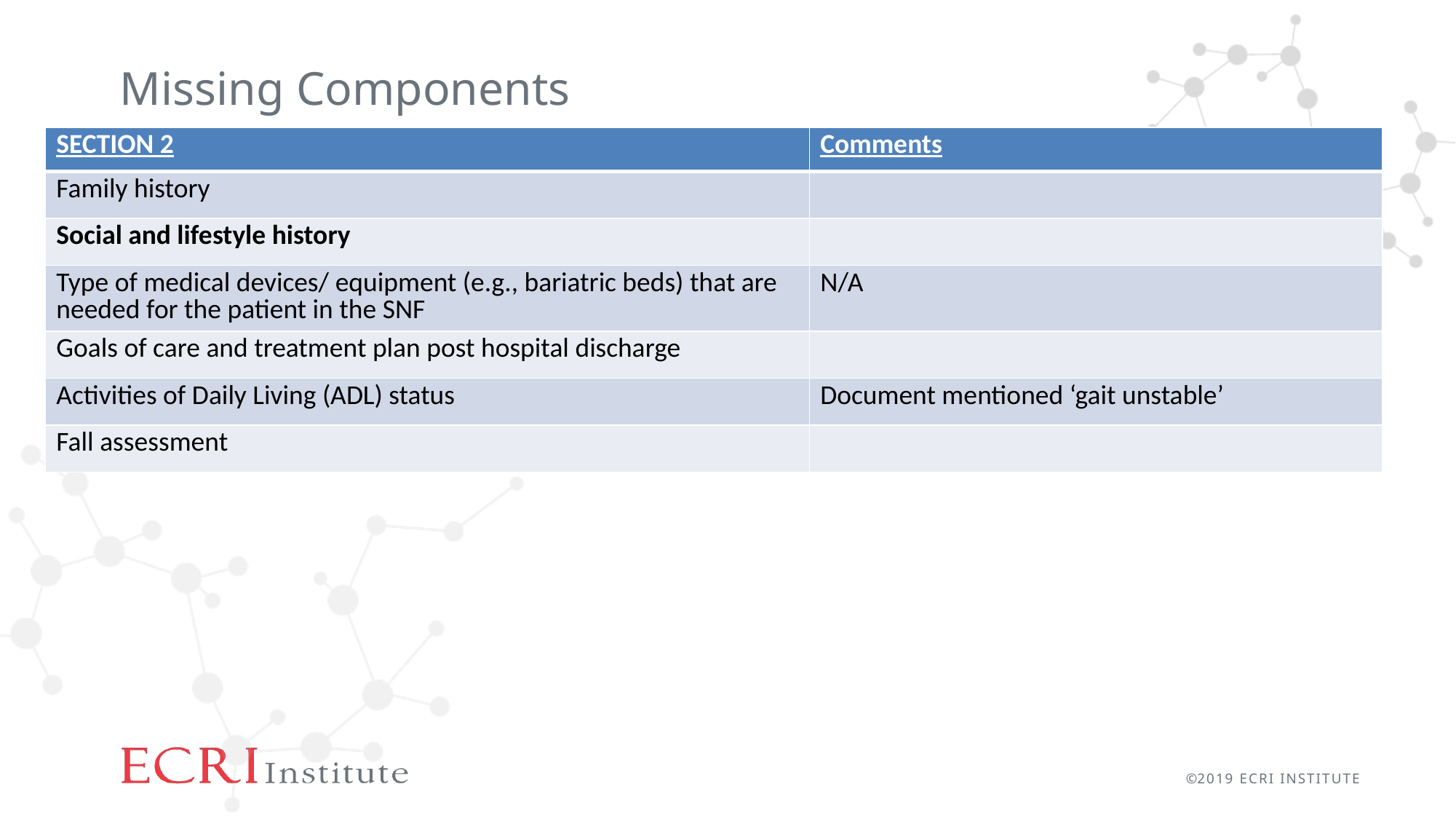

# Missing Components
| SECTION 2 | Comments |
| --- | --- |
| Family history | |
| Social and lifestyle history | |
| Type of medical devices/ equipment (e.g., bariatric beds) that are needed for the patient in the SNF | N/A |
| Goals of care and treatment plan post hospital discharge | |
| Activities of Daily Living (ADL) status | Document mentioned ‘gait unstable’ |
| Fall assessment | |

## Slide 20
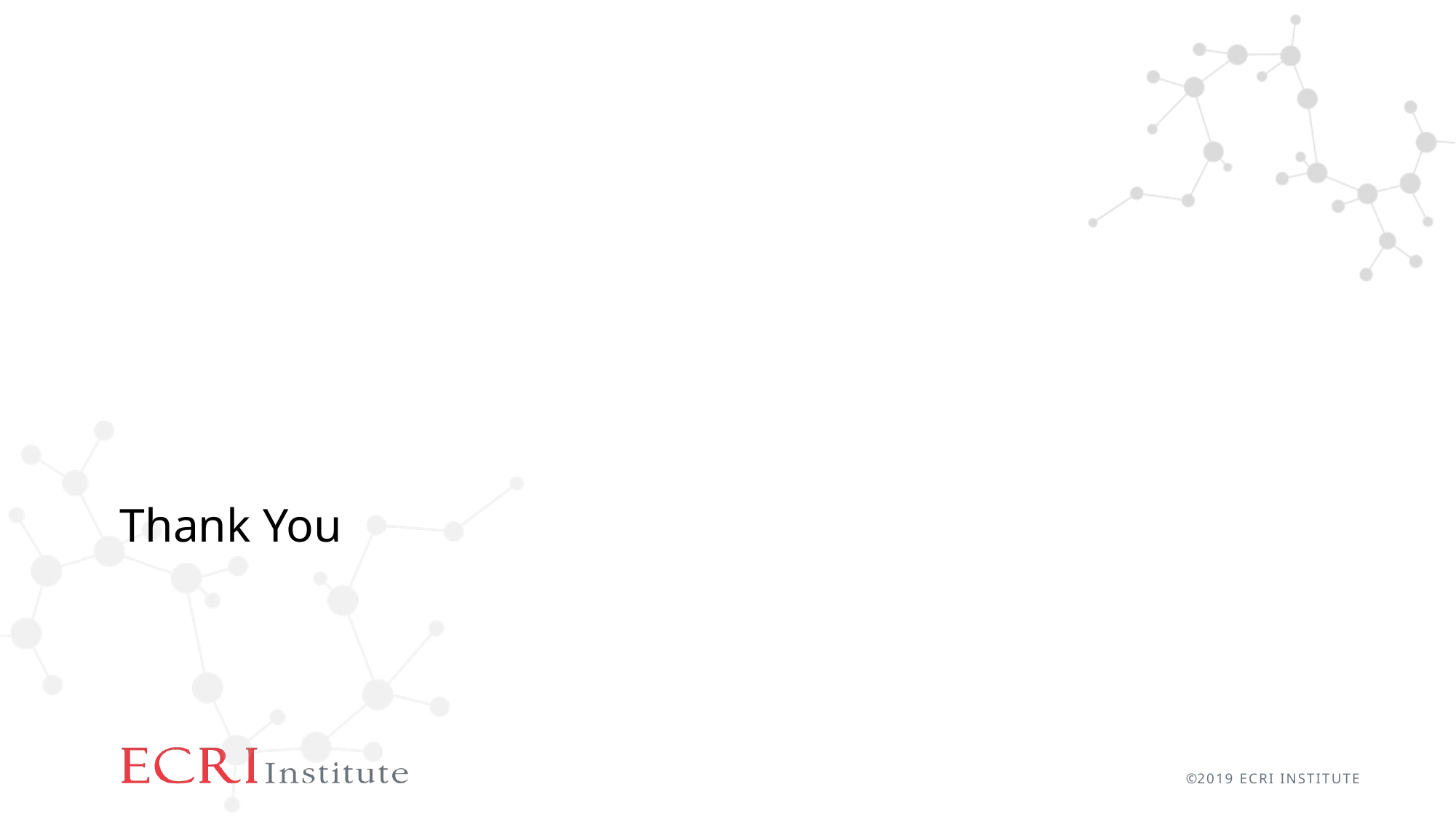

#
