# Supplementary material for: Usability of Electronic Health Record–Generated Discharge Summaries: Heuristic Evaluation
Source: J Med Internet Res. 2021 Apr 15;23(4):e25657. doi: 10.2196/25657 (PMC8085750; doi:10.2196/25657)
Supplement: Multimedia Appendix 11 [file jmir_v23i4e25657_app11.pptx]

## Slide 1
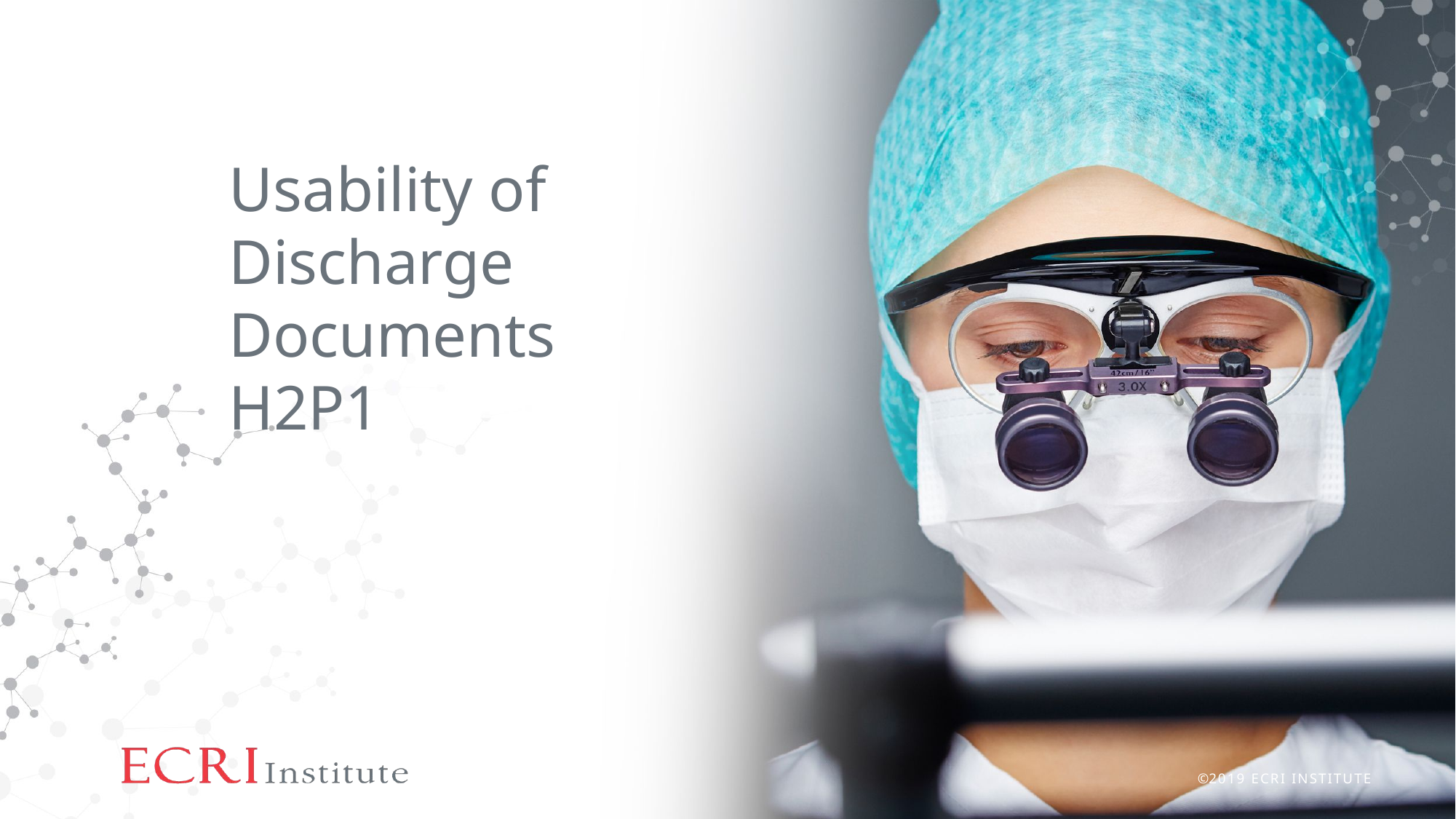

# Usability of Discharge Documents H2P1

## Slide 2
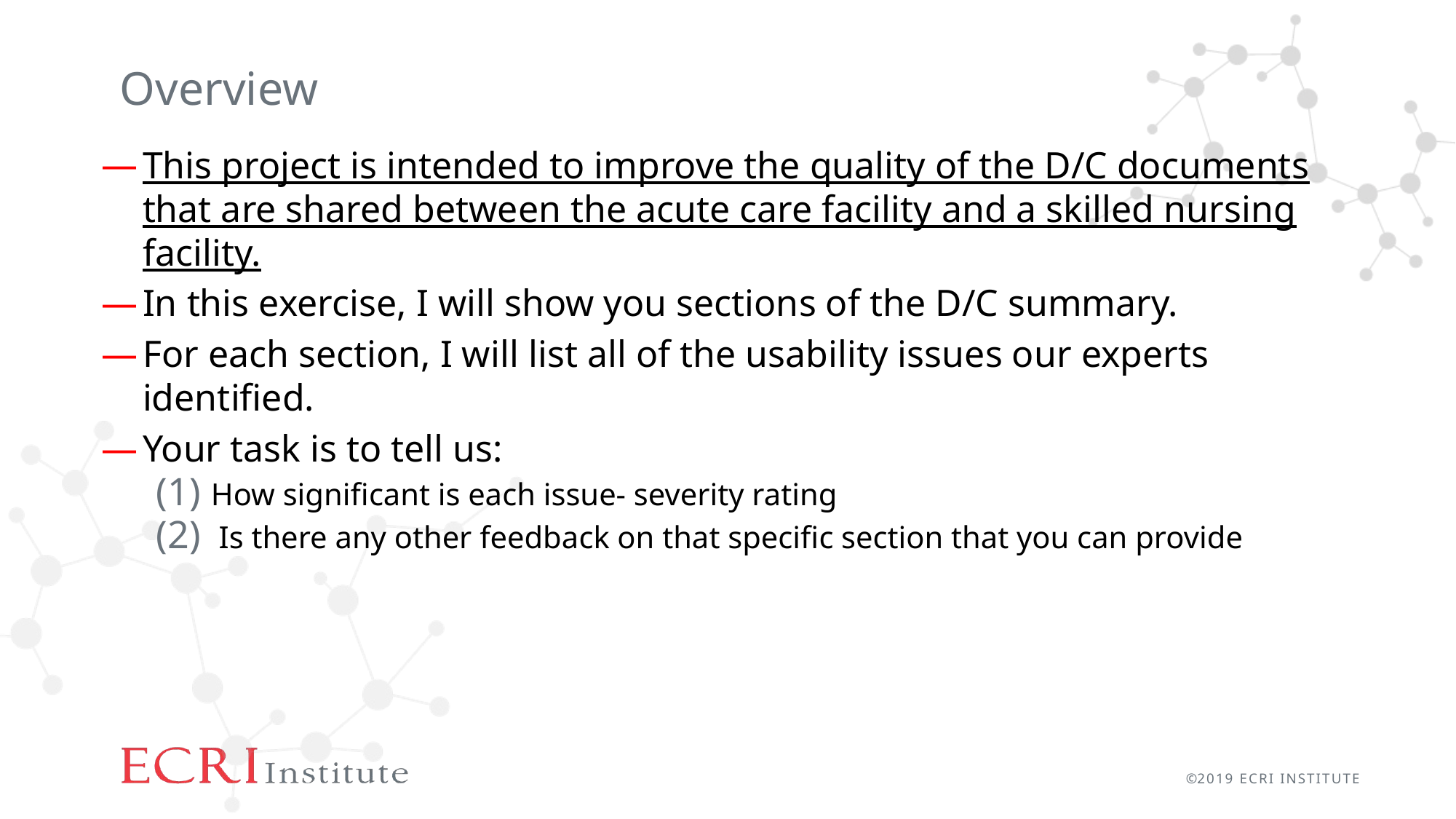

# Overview
This project is intended to improve the quality of the D/C documents that are shared between the acute care facility and a skilled nursing facility.
In this exercise, I will show you sections of the D/C summary.
For each section, I will list all of the usability issues our experts identified.
Your task is to tell us:
How significant is each issue- severity rating
 Is there any other feedback on that specific section that you can provide

## Slide 3
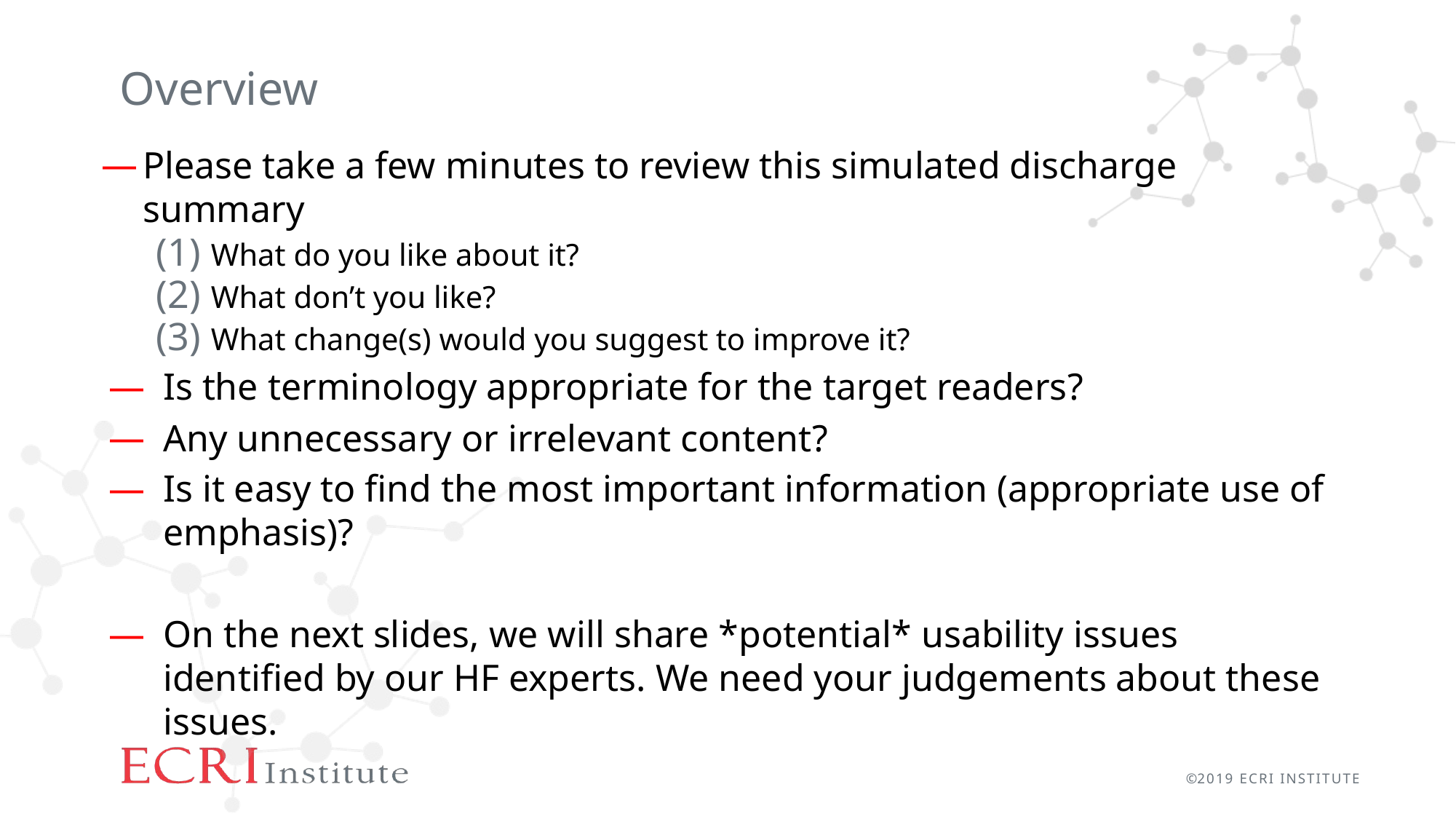

# Overview
Please take a few minutes to review this simulated discharge summary
What do you like about it?
What don’t you like?
What change(s) would you suggest to improve it?
Is the terminology appropriate for the target readers?
Any unnecessary or irrelevant content?
Is it easy to find the most important information (appropriate use of emphasis)?
On the next slides, we will share *potential* usability issues identified by our HF experts. We need your judgements about these issues.

## Slide 4
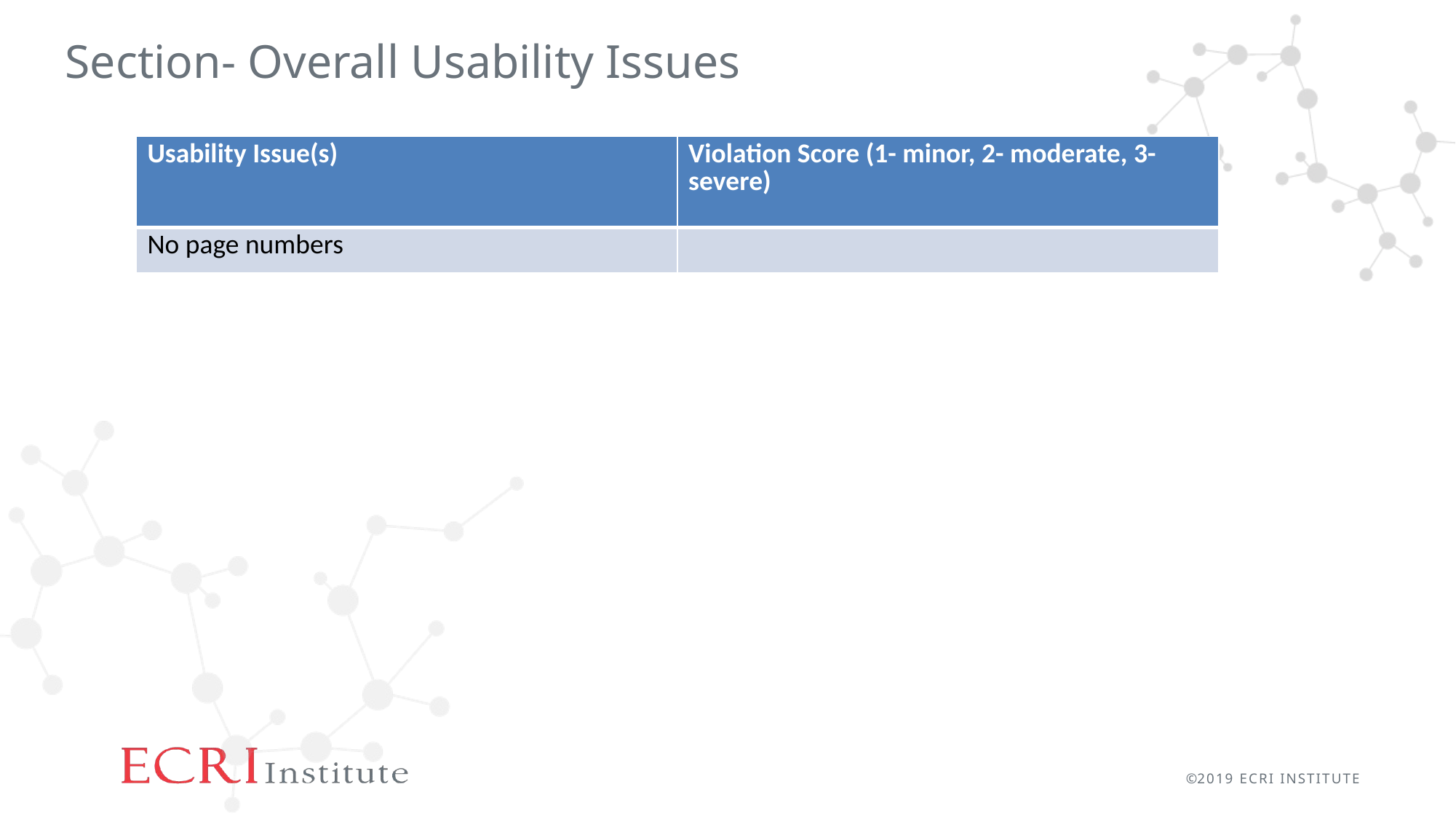

# Section- Overall Usability Issues
| Usability Issue(s) | Violation Score (1- minor, 2- moderate, 3- severe) |
| --- | --- |
| No page numbers | |

## Slide 5
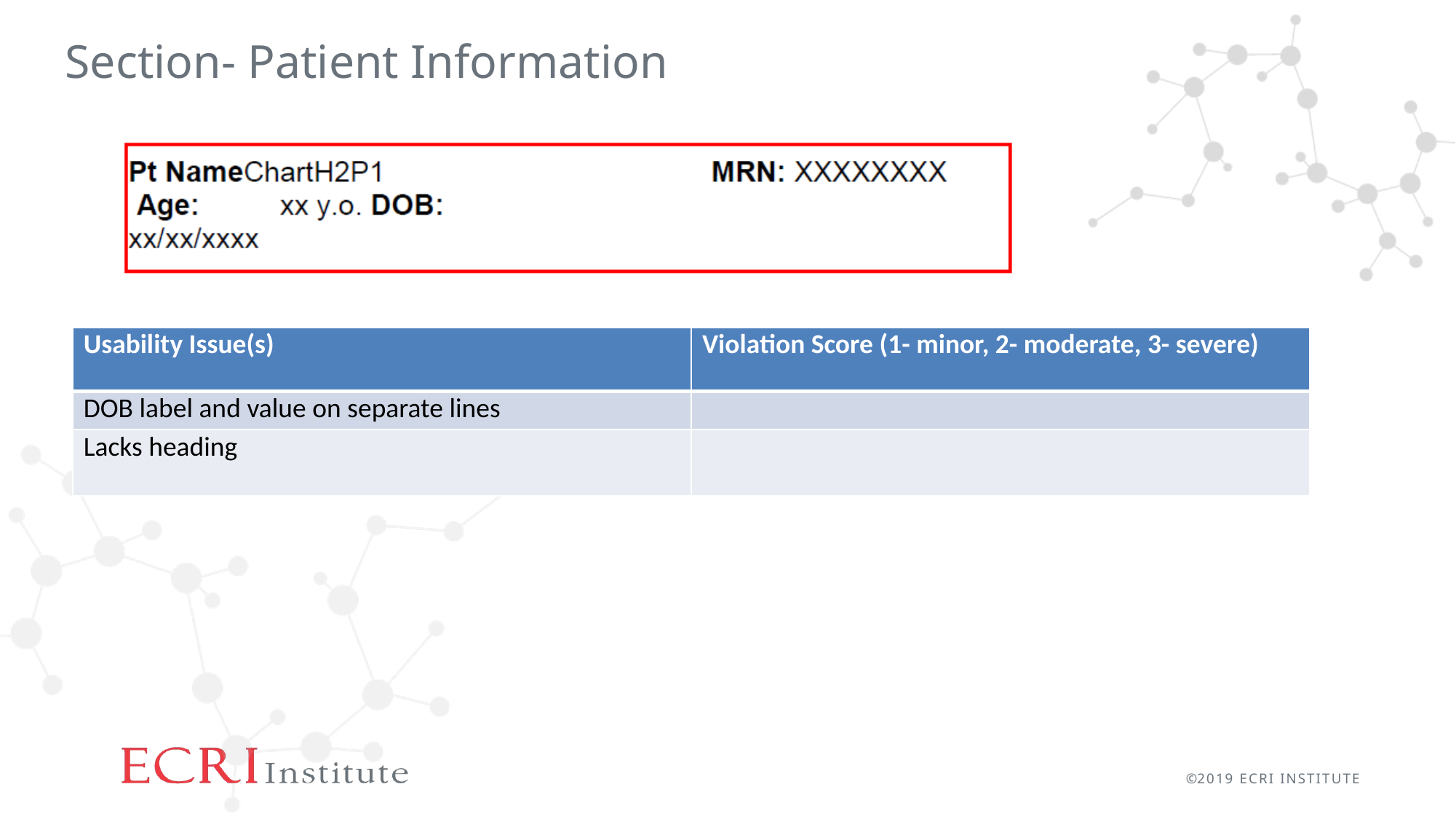

# Section- Patient Information
| Usability Issue(s) | Violation Score (1- minor, 2- moderate, 3- severe) |
| --- | --- |
| DOB label and value on separate lines | |
| Lacks heading | |

## Slide 6
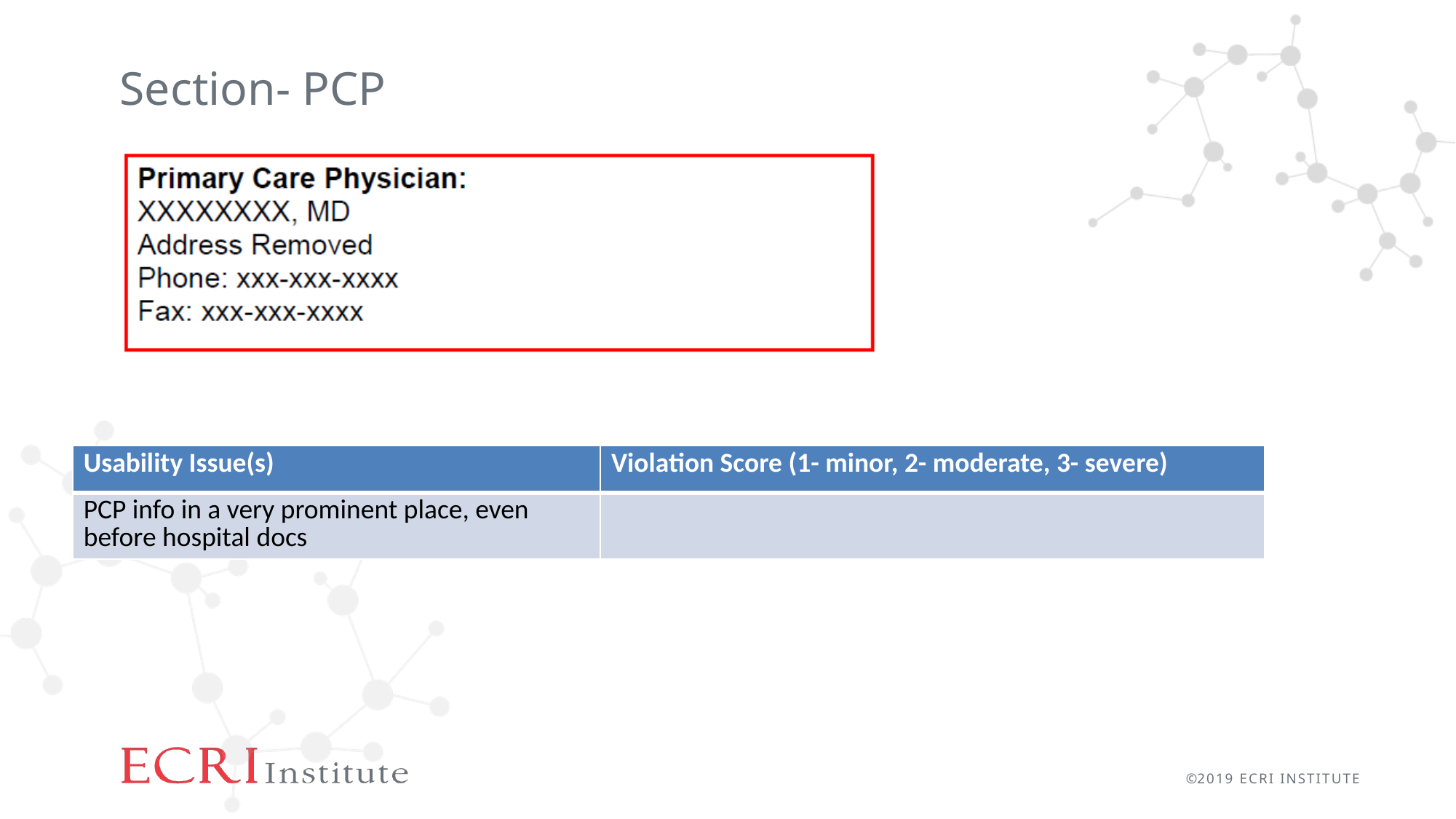

# Section- PCP
| Usability Issue(s) | Violation Score (1- minor, 2- moderate, 3- severe) |
| --- | --- |
| PCP info in a very prominent place, even before hospital docs | |

## Slide 7
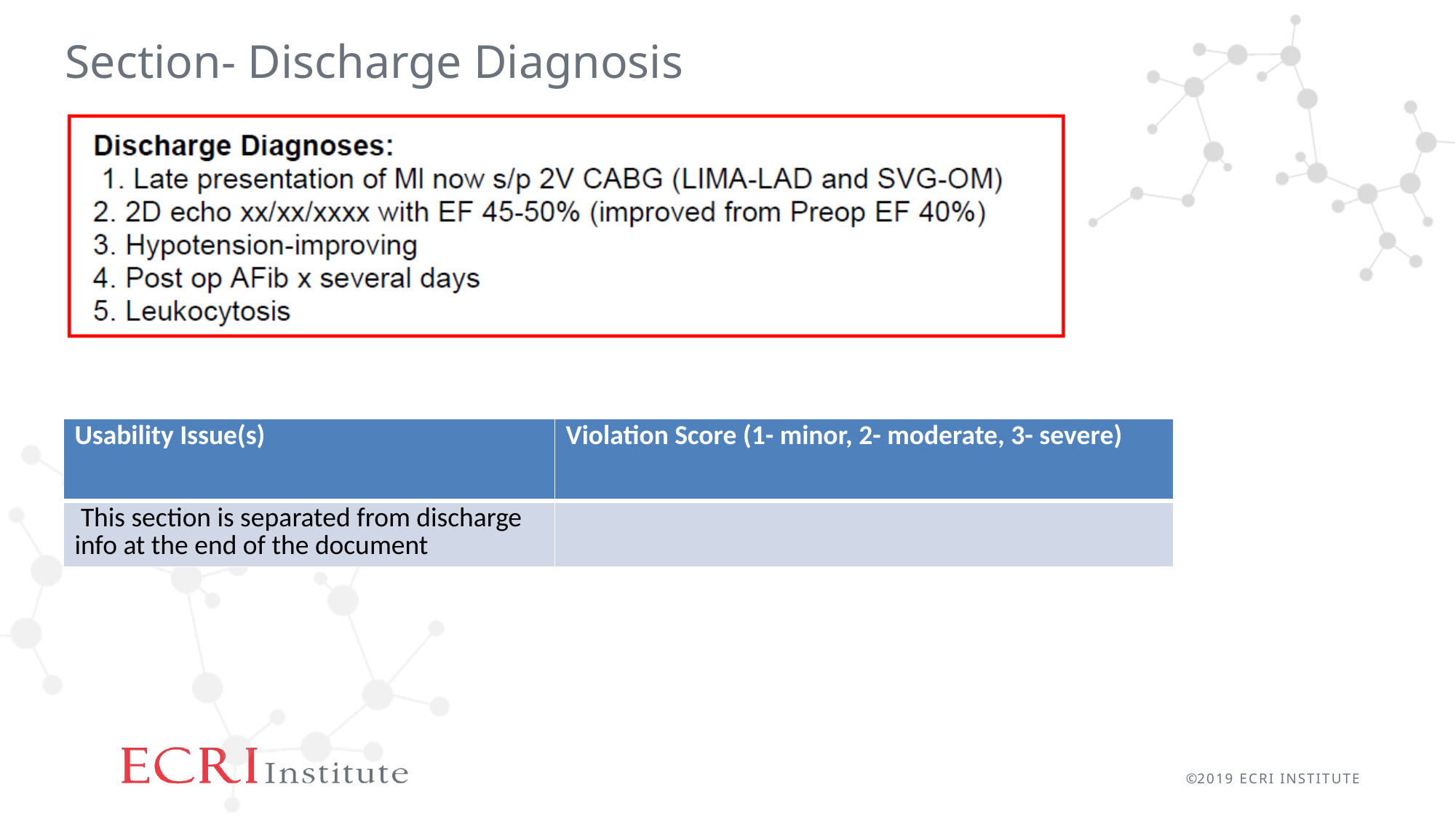

# Section- Discharge Diagnosis
| Usability Issue(s) | Violation Score (1- minor, 2- moderate, 3- severe) |
| --- | --- |
| This section is separated from discharge info at the end of the document | |

## Slide 8
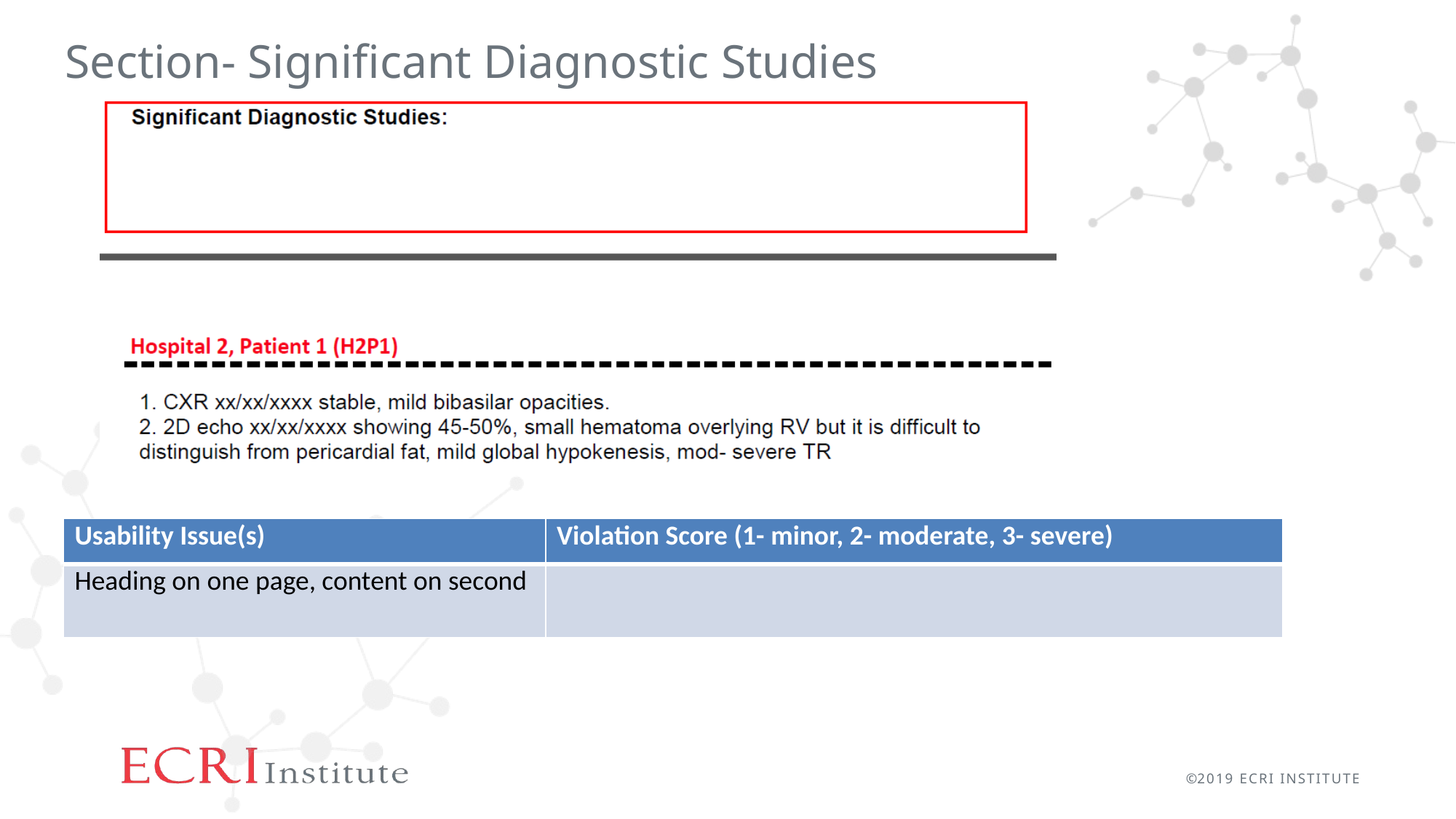

# Section- Significant Diagnostic Studies
| Usability Issue(s) | Violation Score (1- minor, 2- moderate, 3- severe) |
| --- | --- |
| Heading on one page, content on second | |

## Slide 9
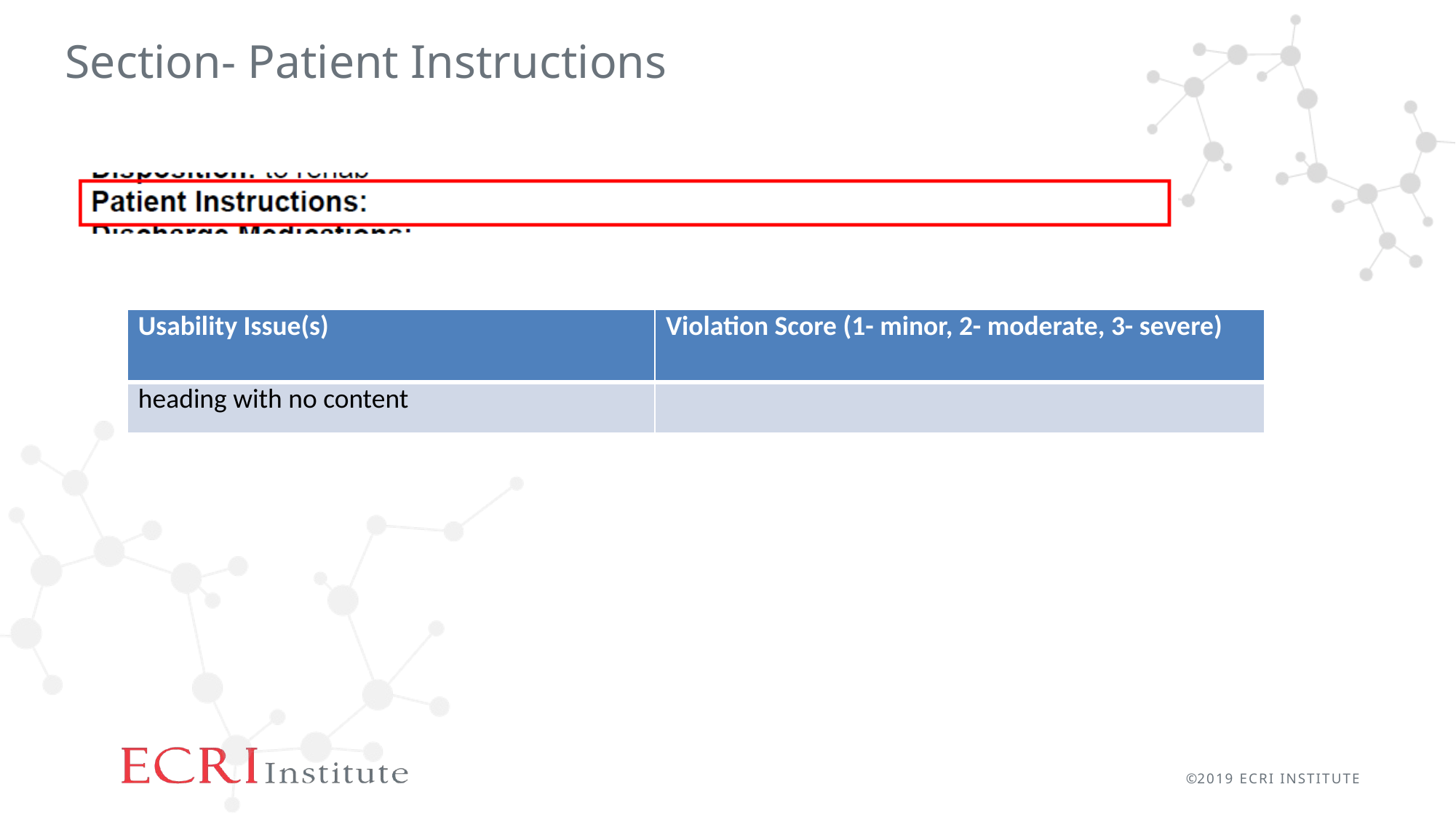

# Section- Patient Instructions
| Usability Issue(s) | Violation Score (1- minor, 2- moderate, 3- severe) |
| --- | --- |
| heading with no content | |

## Slide 10
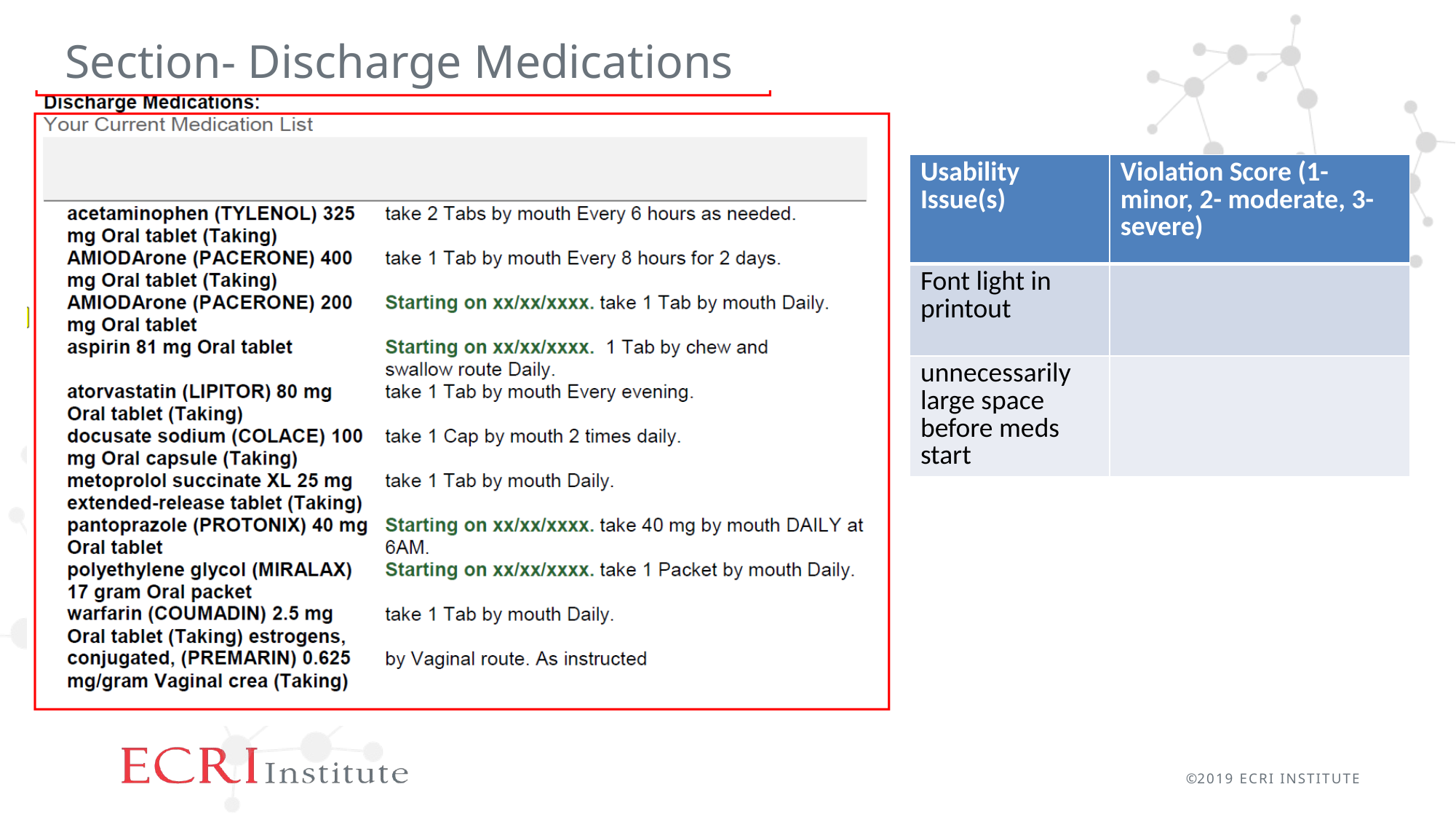

# Section- Discharge Medications
| Usability Issue(s) | Violation Score (1- minor, 2- moderate, 3- severe) |
| --- | --- |
| Font light in printout | |
| unnecessarily large space before meds start | |

## Slide 11
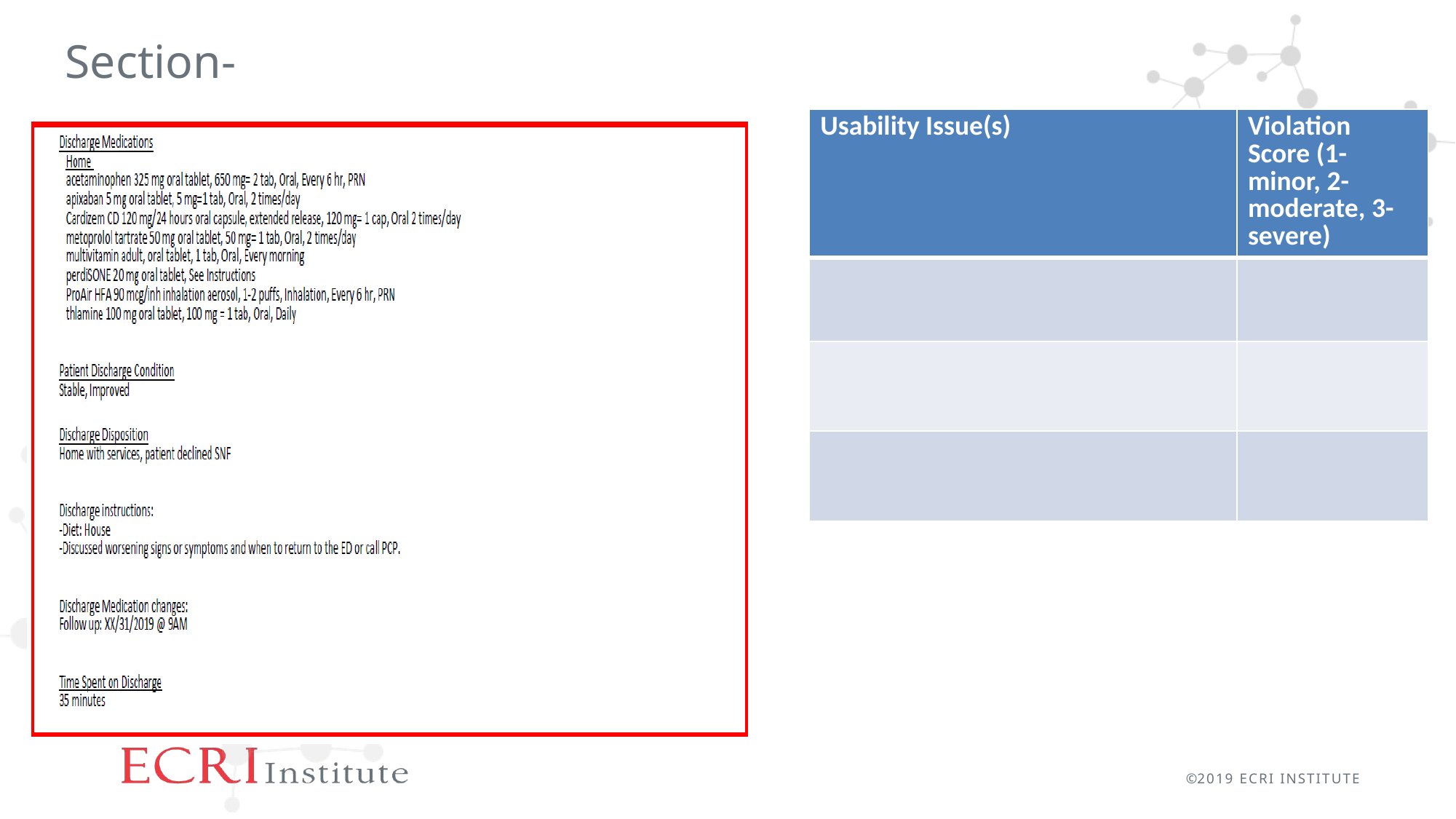

# Section-
| Usability Issue(s) | Violation Score (1- minor, 2- moderate, 3- severe) |
| --- | --- |
| | |
| | |
| | |

## Slide 12
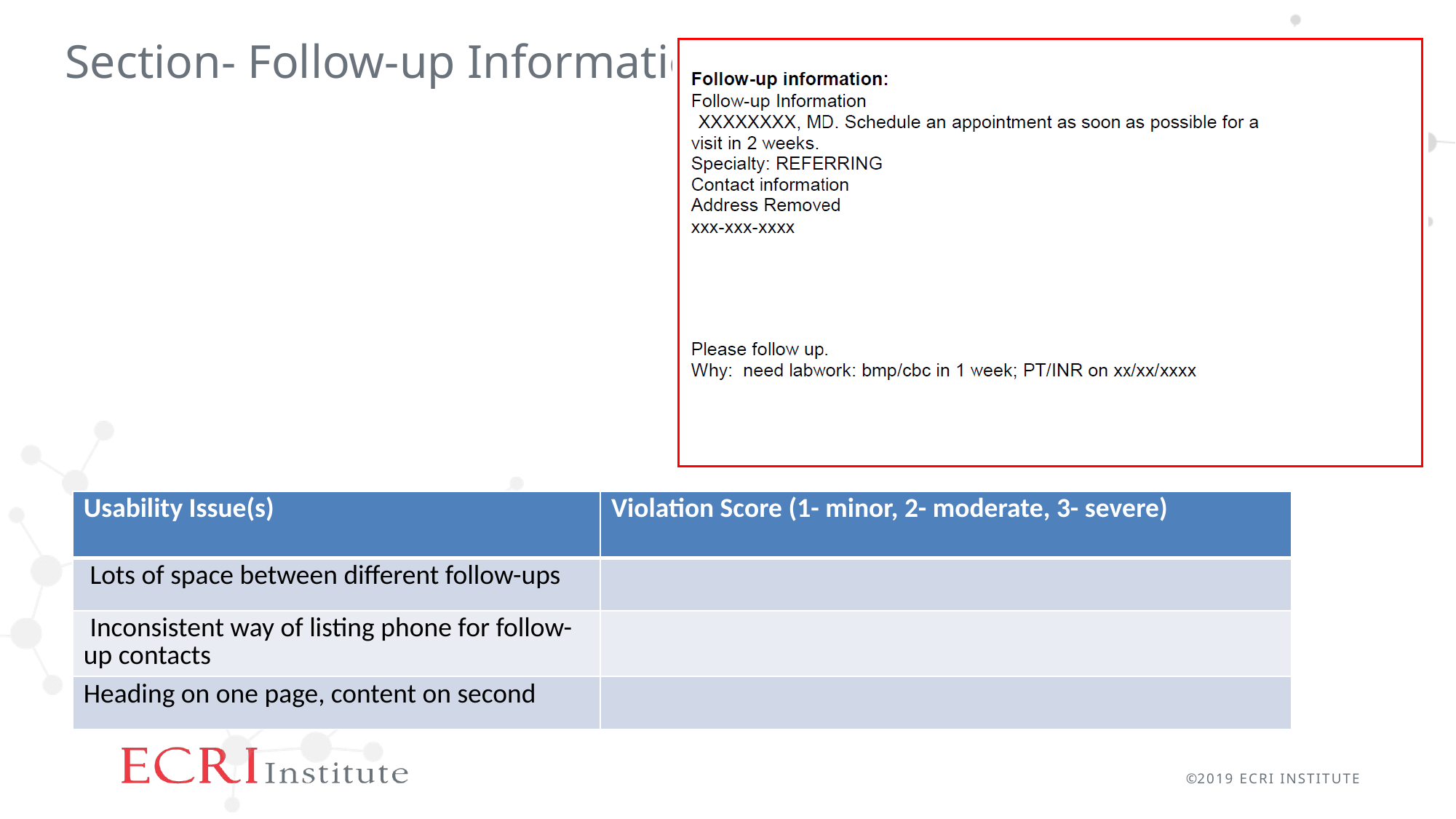

# Section- Follow-up Information
| Usability Issue(s) | Violation Score (1- minor, 2- moderate, 3- severe) |
| --- | --- |
| Lots of space between different follow-ups | |
| Inconsistent way of listing phone for follow-up contacts | |
| Heading on one page, content on second | |

## Slide 13
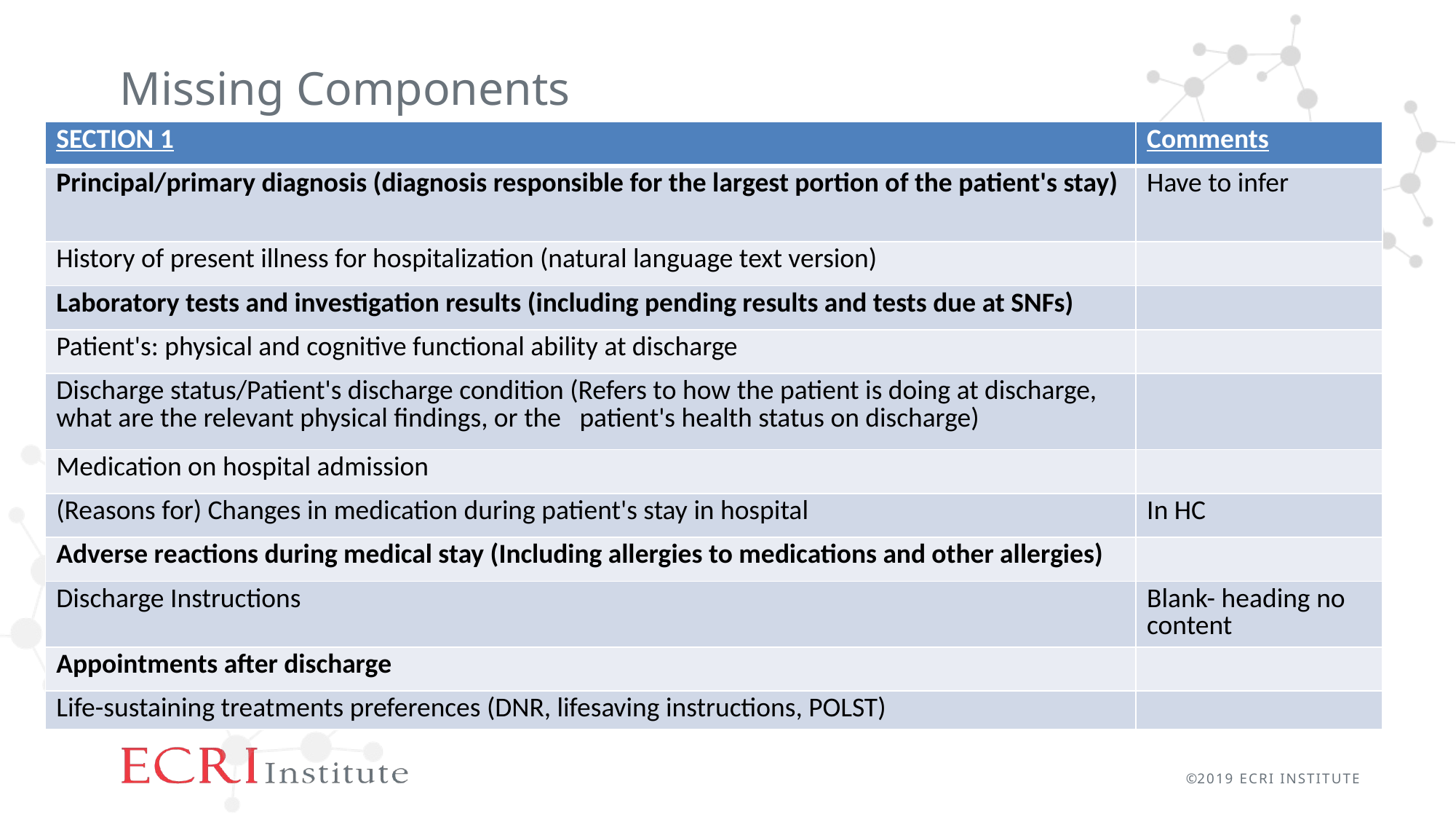

# Missing Components
| SECTION 1 | Comments |
| --- | --- |
| Principal/primary diagnosis (diagnosis responsible for the largest portion of the patient's stay) | Have to infer |
| History of present illness for hospitalization (natural language text version) | |
| Laboratory tests and investigation results (including pending results and tests due at SNFs) | |
| Patient's: physical and cognitive functional ability at discharge | |
| Discharge status/Patient's discharge condition (Refers to how the patient is doing at discharge, what are the relevant physical findings, or the patient's health status on discharge) | |
| Medication on hospital admission | |
| (Reasons for) Changes in medication during patient's stay in hospital | In HC |
| Adverse reactions during medical stay (Including allergies to medications and other allergies) | |
| Discharge Instructions | Blank- heading no content |
| Appointments after discharge | |
| Life-sustaining treatments preferences (DNR, lifesaving instructions, POLST) | |

## Slide 14
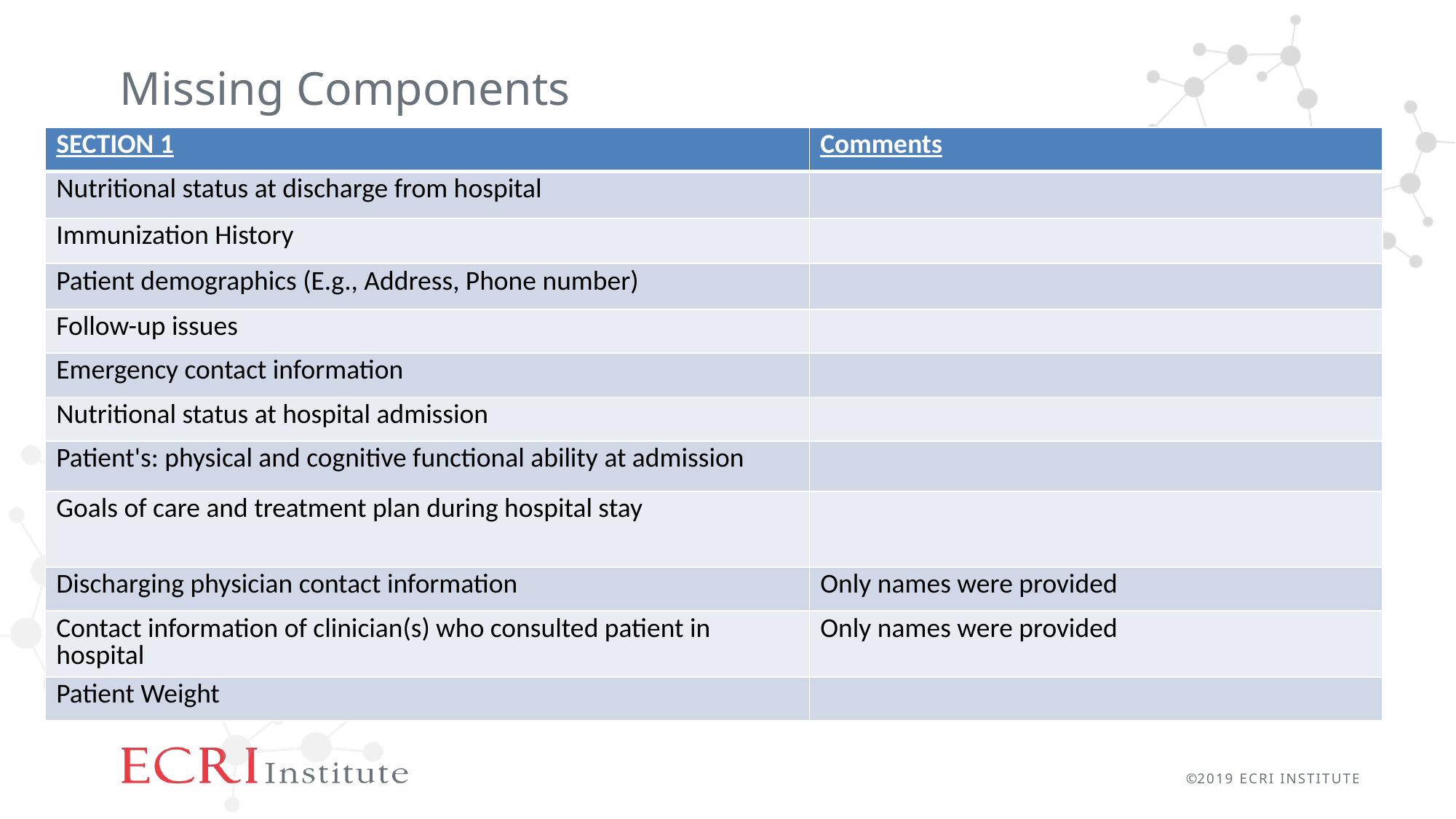

# Missing Components
| SECTION 1 | Comments |
| --- | --- |
| Nutritional status at discharge from hospital | |
| Immunization History | |
| Patient demographics (E.g., Address, Phone number) | |
| Follow-up issues | |
| Emergency contact information | |
| Nutritional status at hospital admission | |
| Patient's: physical and cognitive functional ability at admission | |
| Goals of care and treatment plan during hospital stay | |
| Discharging physician contact information | Only names were provided |
| Contact information of clinician(s) who consulted patient in hospital | Only names were provided |
| Patient Weight | |

## Slide 15
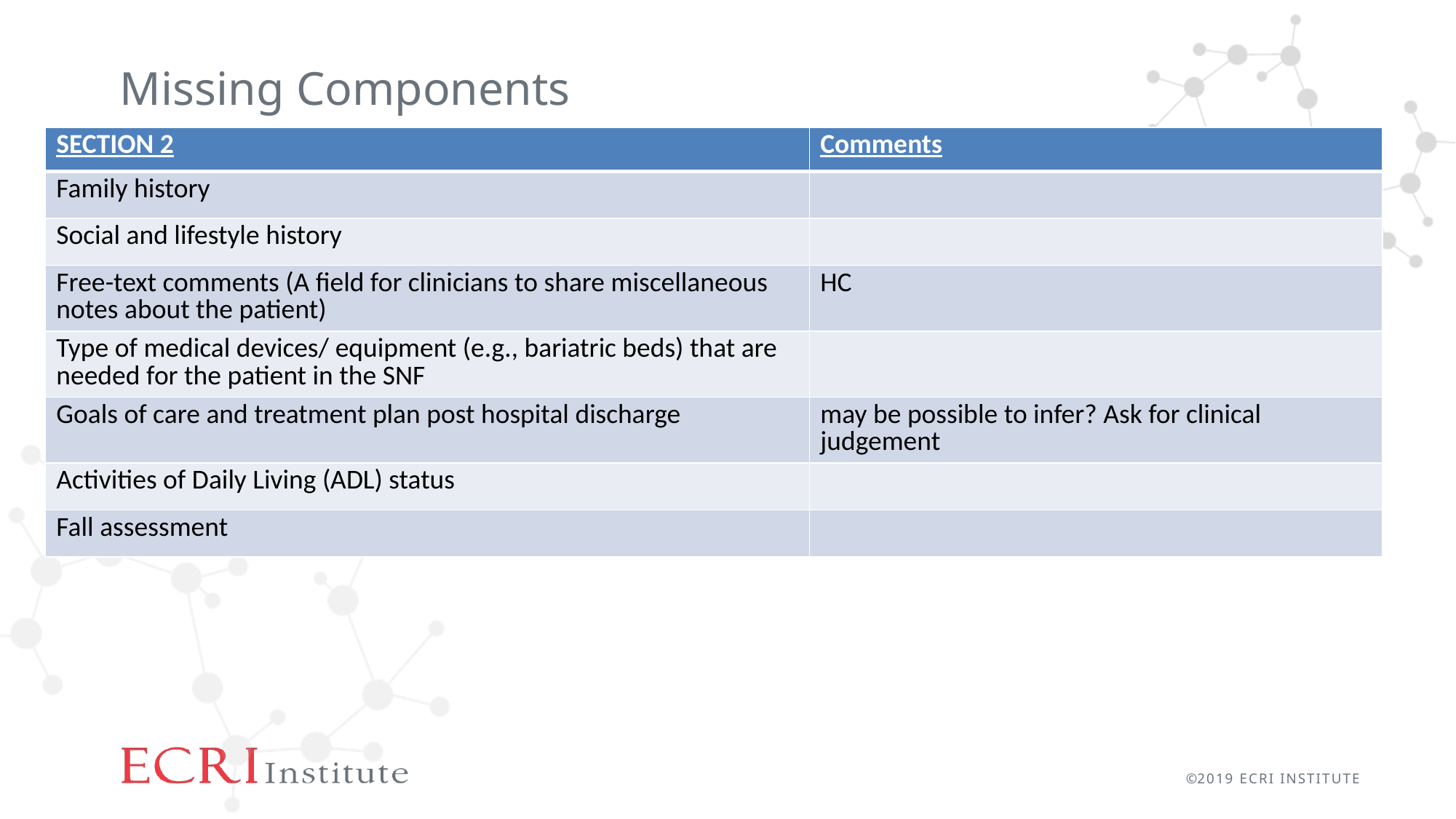

# Missing Components
| SECTION 2 | Comments |
| --- | --- |
| Family history | |
| Social and lifestyle history | |
| Free-text comments (A field for clinicians to share miscellaneous notes about the patient) | HC |
| Type of medical devices/ equipment (e.g., bariatric beds) that are needed for the patient in the SNF | |
| Goals of care and treatment plan post hospital discharge | may be possible to infer? Ask for clinical judgement |
| Activities of Daily Living (ADL) status | |
| Fall assessment | |

## Slide 16
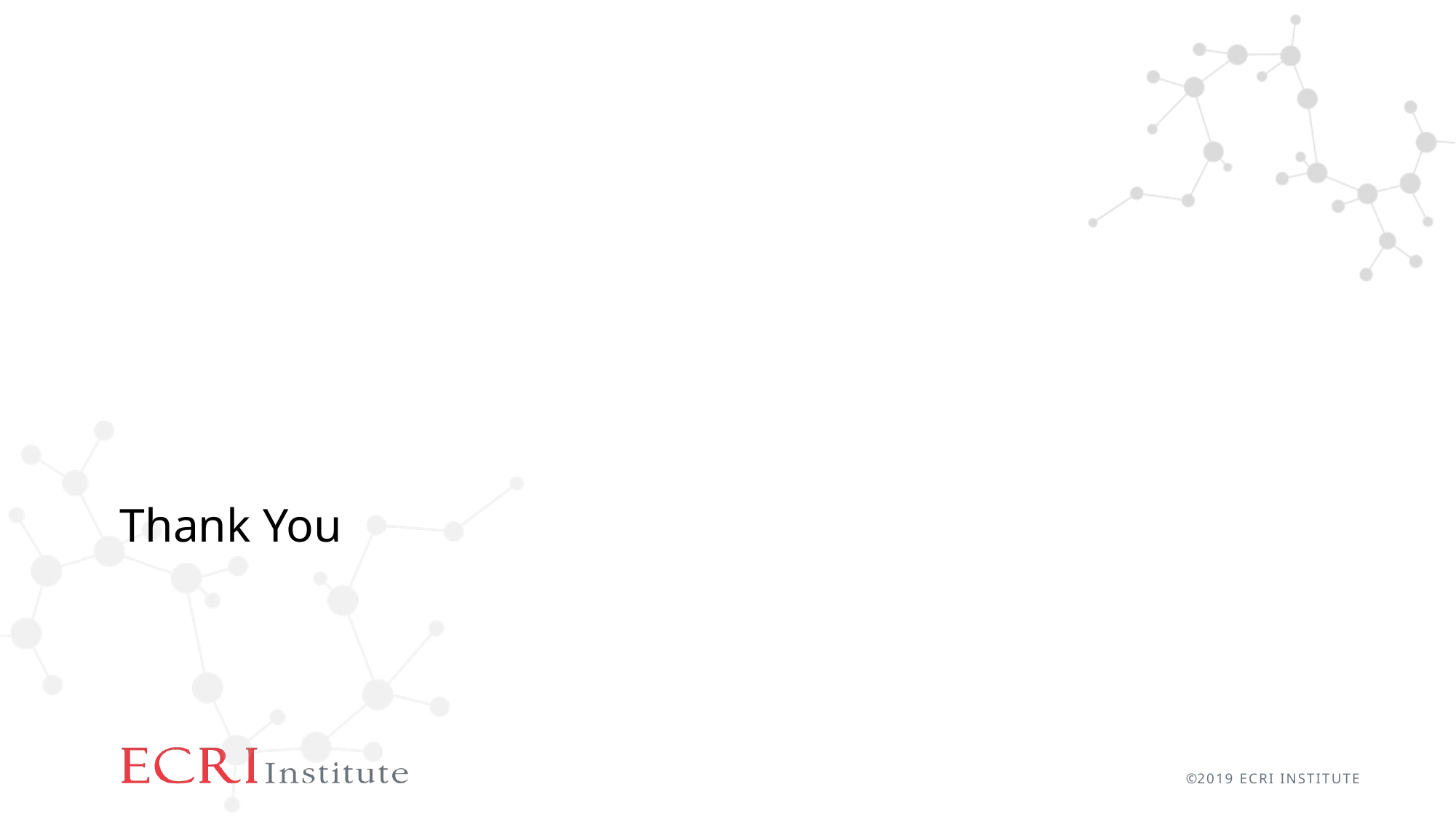

#
