# Supplementary material for: Usability of Electronic Health Record–Generated Discharge Summaries: Heuristic Evaluation
Source: J Med Internet Res. 2021 Apr 15;23(4):e25657. doi: 10.2196/25657 (PMC8085750; doi:10.2196/25657)
Supplement: Multimedia Appendix 12 [file jmir_v23i4e25657_app12.pptx]

## Slide 1
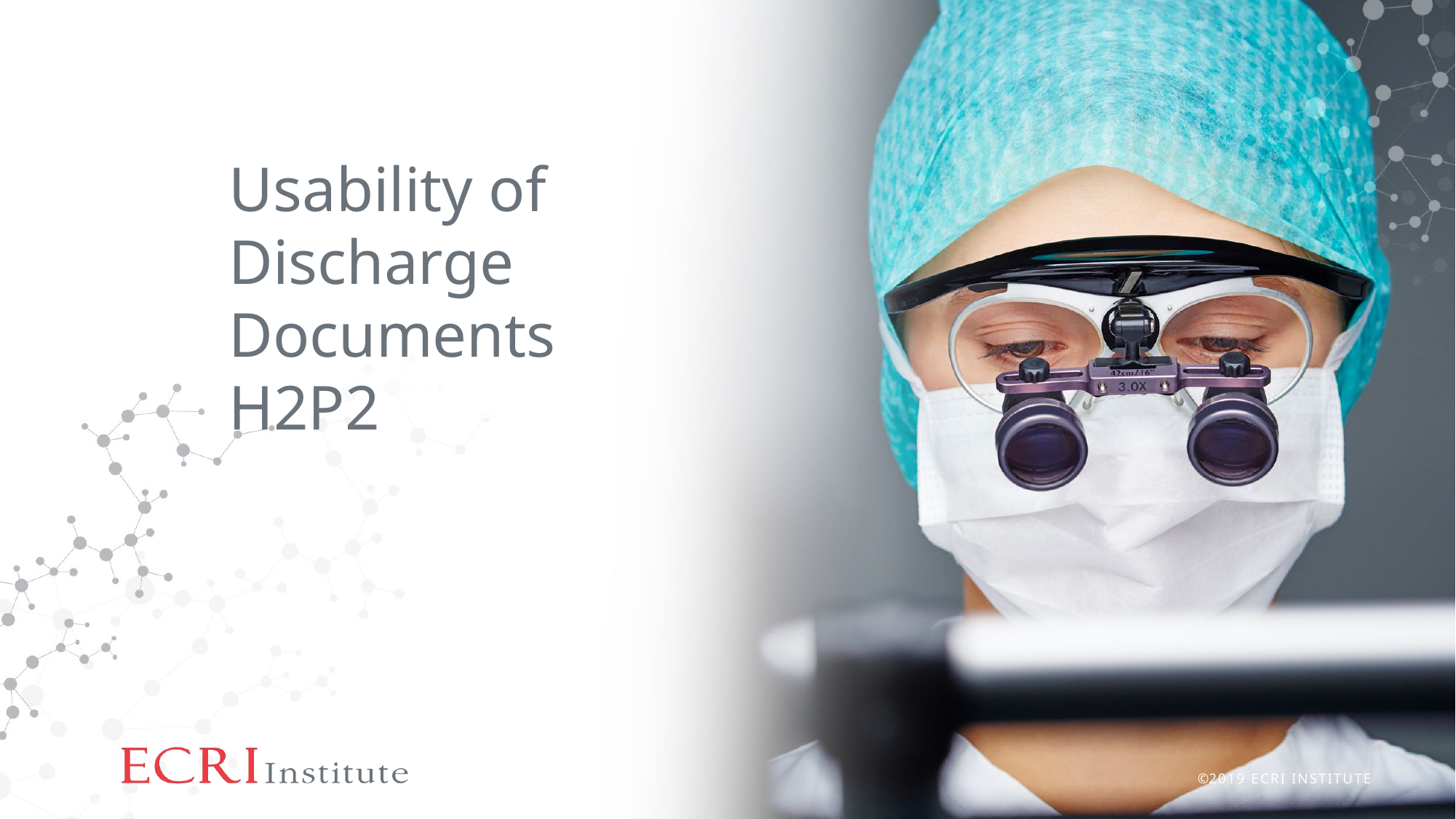

# Usability of Discharge Documents H2P2

## Slide 2
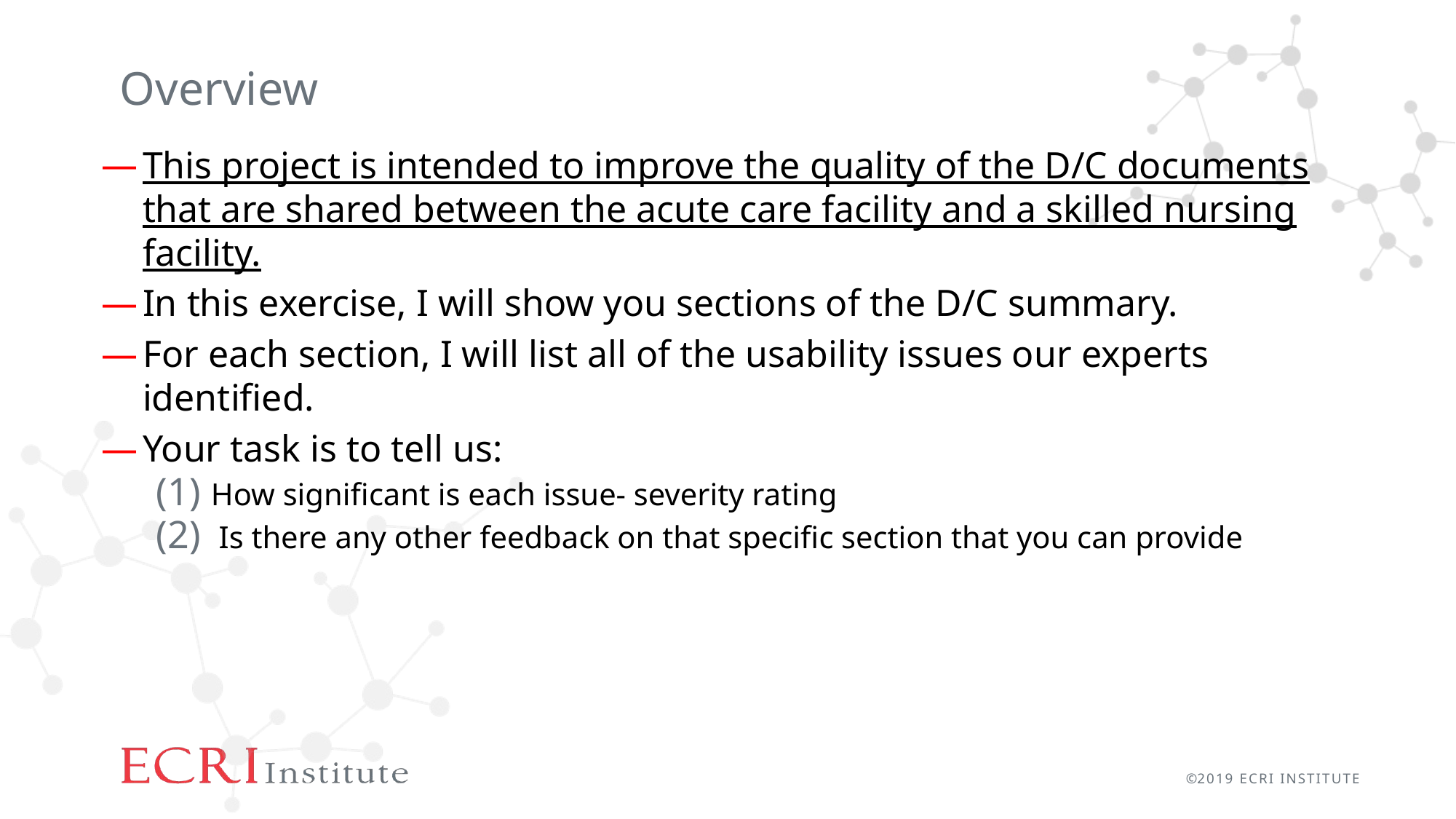

# Overview
This project is intended to improve the quality of the D/C documents that are shared between the acute care facility and a skilled nursing facility.
In this exercise, I will show you sections of the D/C summary.
For each section, I will list all of the usability issues our experts identified.
Your task is to tell us:
How significant is each issue- severity rating
 Is there any other feedback on that specific section that you can provide

## Slide 3
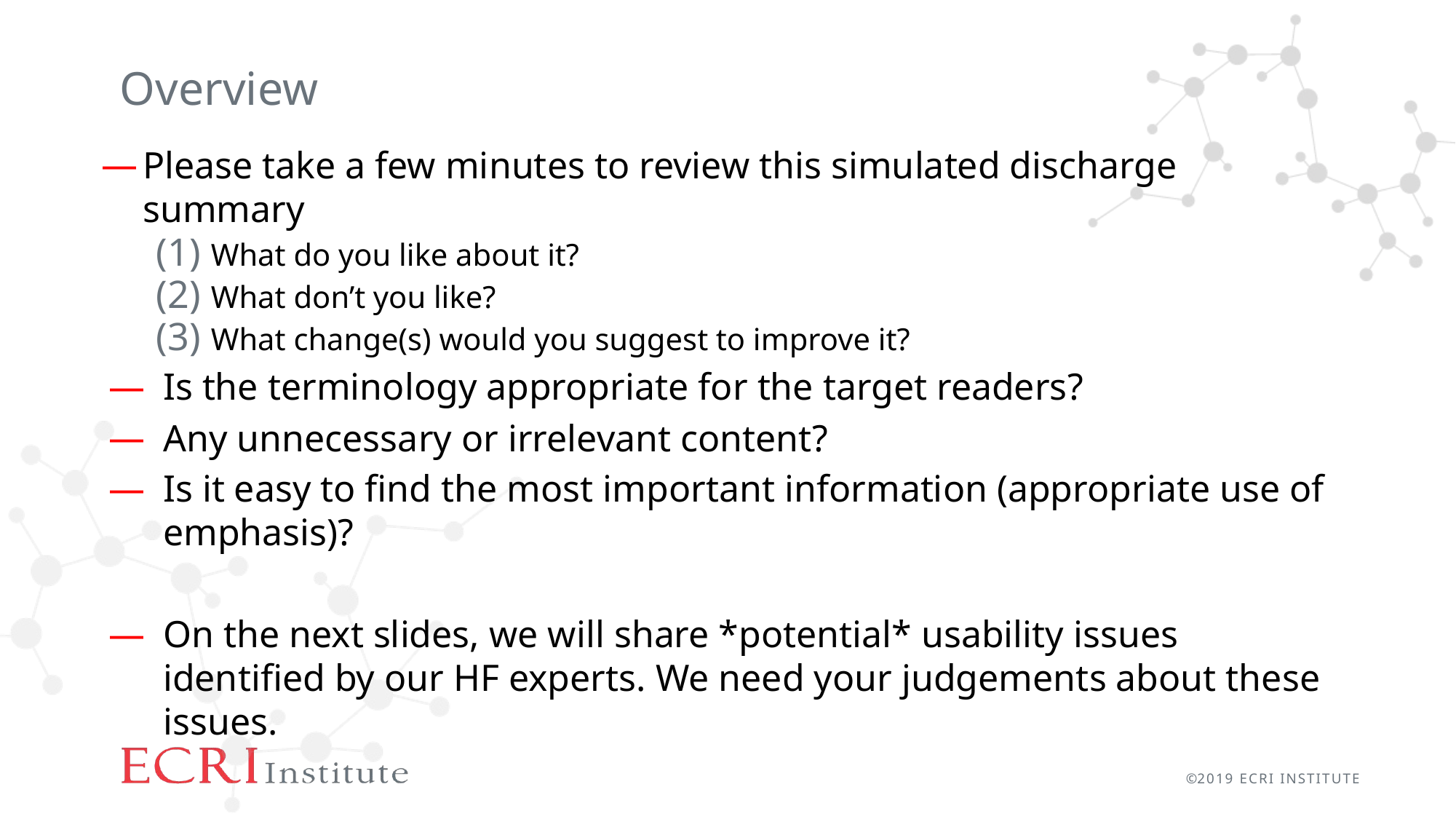

# Overview
Please take a few minutes to review this simulated discharge summary
What do you like about it?
What don’t you like?
What change(s) would you suggest to improve it?
Is the terminology appropriate for the target readers?
Any unnecessary or irrelevant content?
Is it easy to find the most important information (appropriate use of emphasis)?
On the next slides, we will share *potential* usability issues identified by our HF experts. We need your judgements about these issues.

## Slide 4
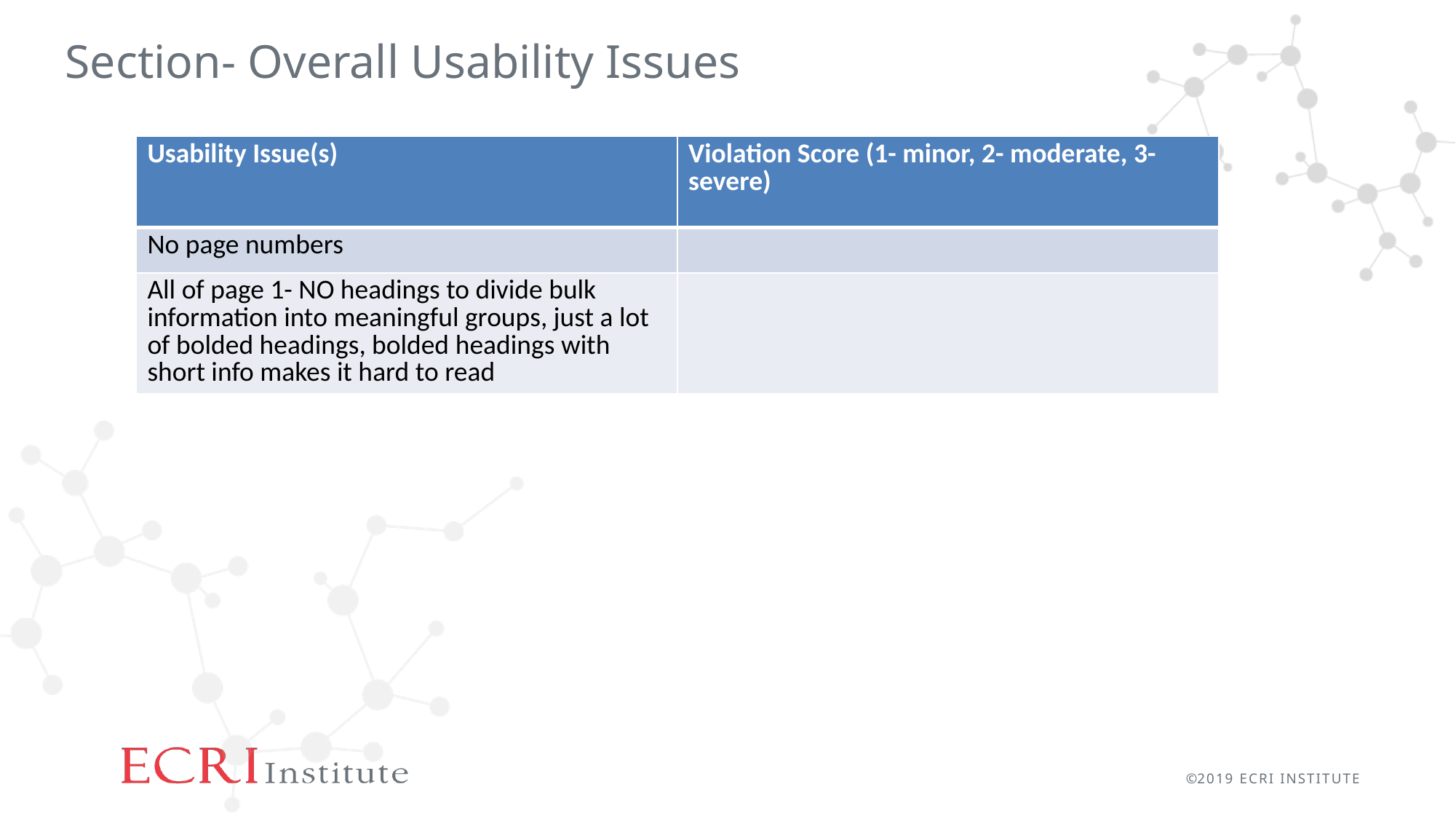

# Section- Overall Usability Issues
| Usability Issue(s) | Violation Score (1- minor, 2- moderate, 3- severe) |
| --- | --- |
| No page numbers | |
| All of page 1- NO headings to divide bulk information into meaningful groups, just a lot of bolded headings, bolded headings with short info makes it hard to read | |

## Slide 5
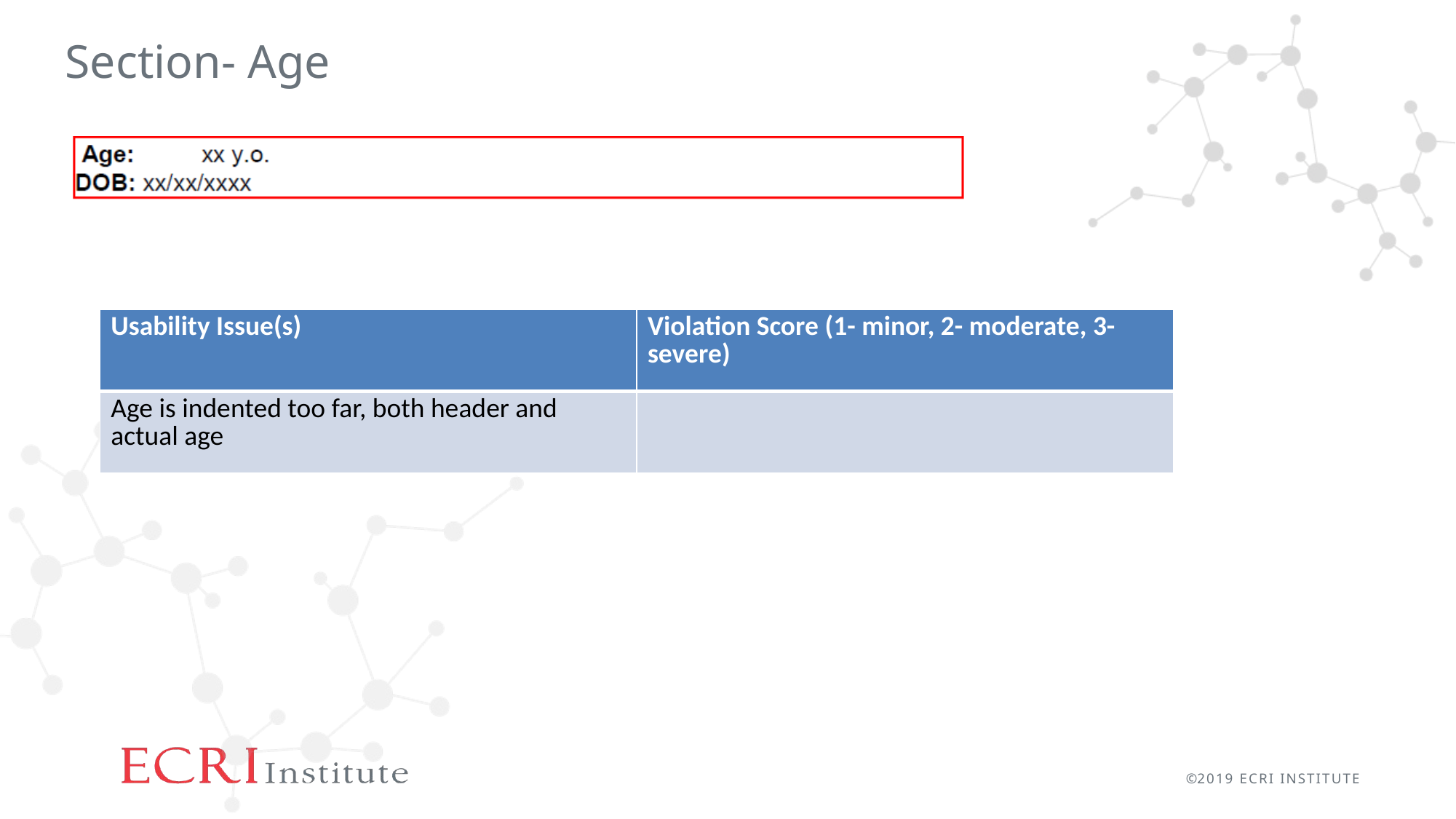

# Section- Age
| Usability Issue(s) | Violation Score (1- minor, 2- moderate, 3- severe) |
| --- | --- |
| Age is indented too far, both header and actual age | |

## Slide 6
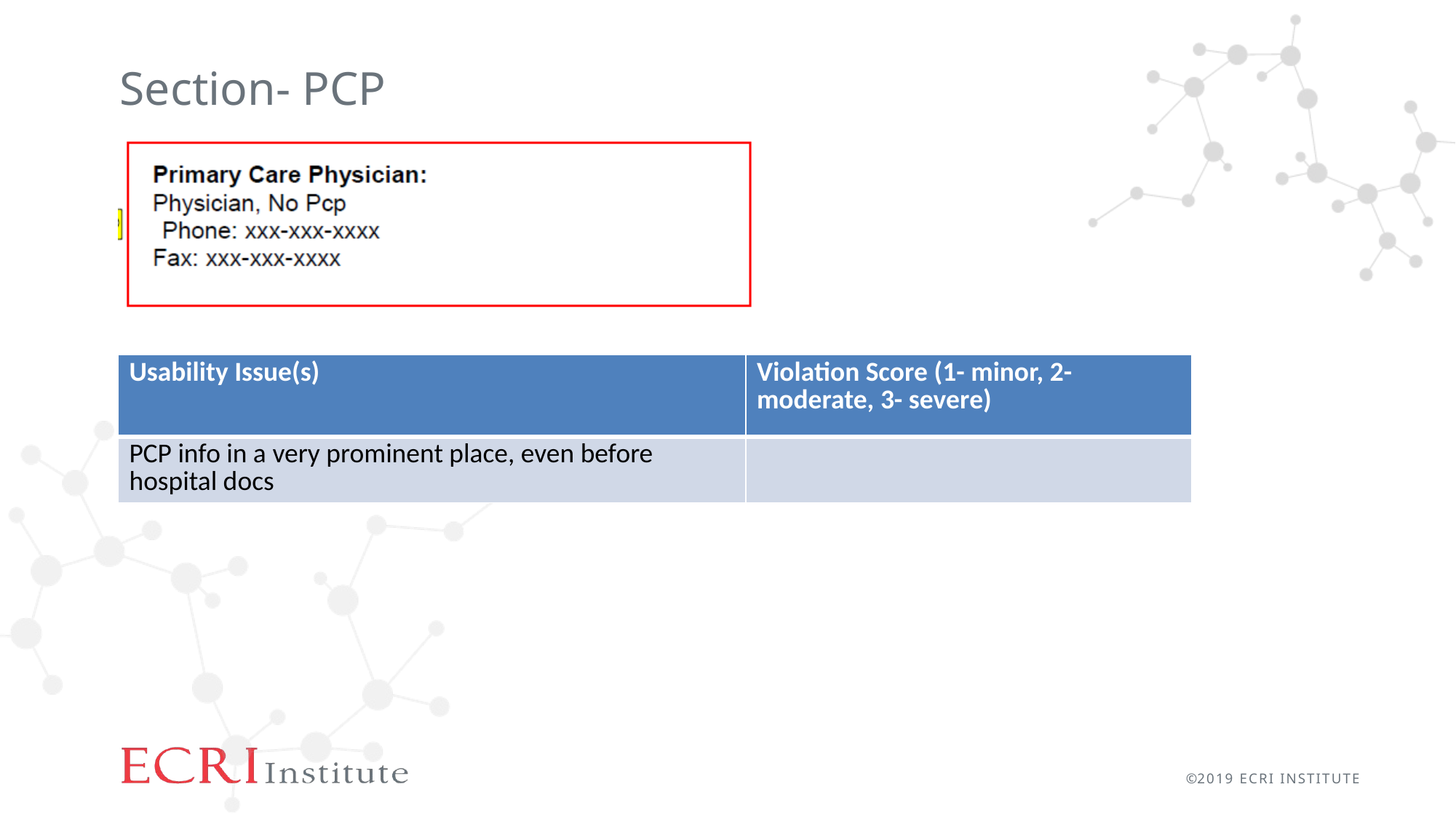

# Section- PCP
| Usability Issue(s) | Violation Score (1- minor, 2- moderate, 3- severe) |
| --- | --- |
| PCP info in a very prominent place, even before hospital docs | |

## Slide 7
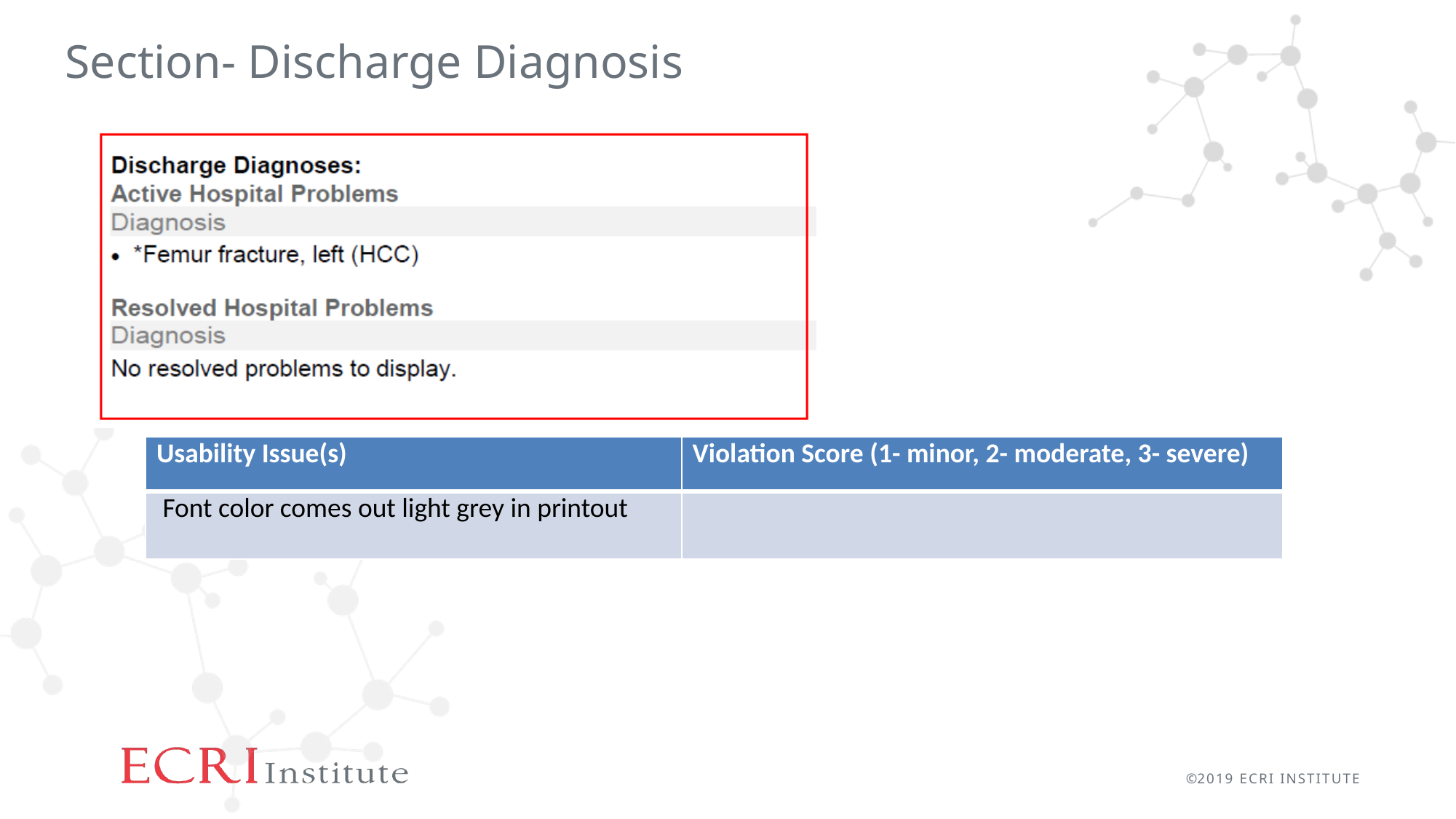

# Section- Discharge Diagnosis
| Usability Issue(s) | Violation Score (1- minor, 2- moderate, 3- severe) |
| --- | --- |
| Font color comes out light grey in printout | |

## Slide 8
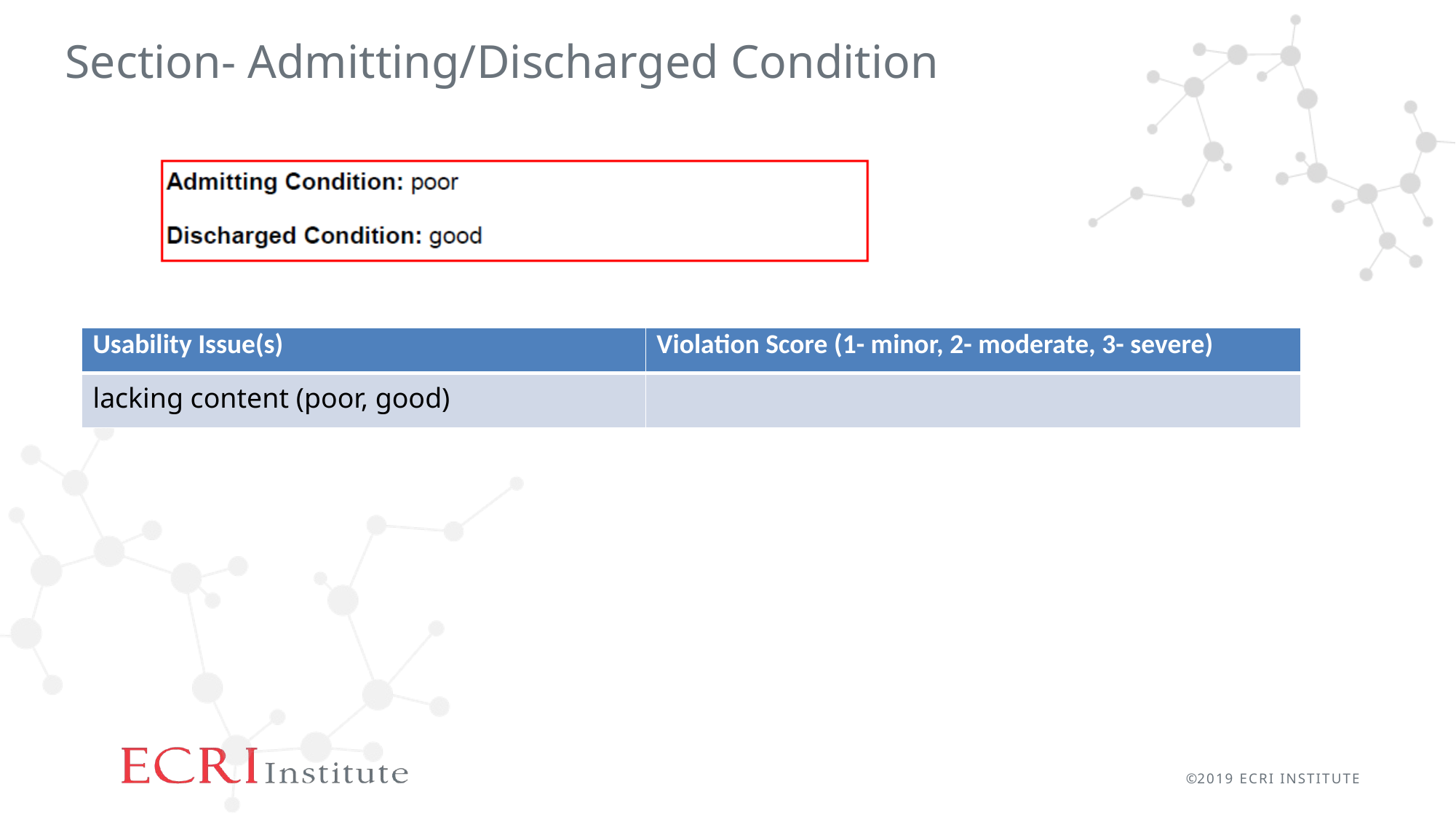

# Section- Admitting/Discharged Condition
| Usability Issue(s) | Violation Score (1- minor, 2- moderate, 3- severe) |
| --- | --- |
| lacking content (poor, good) | |

## Slide 9
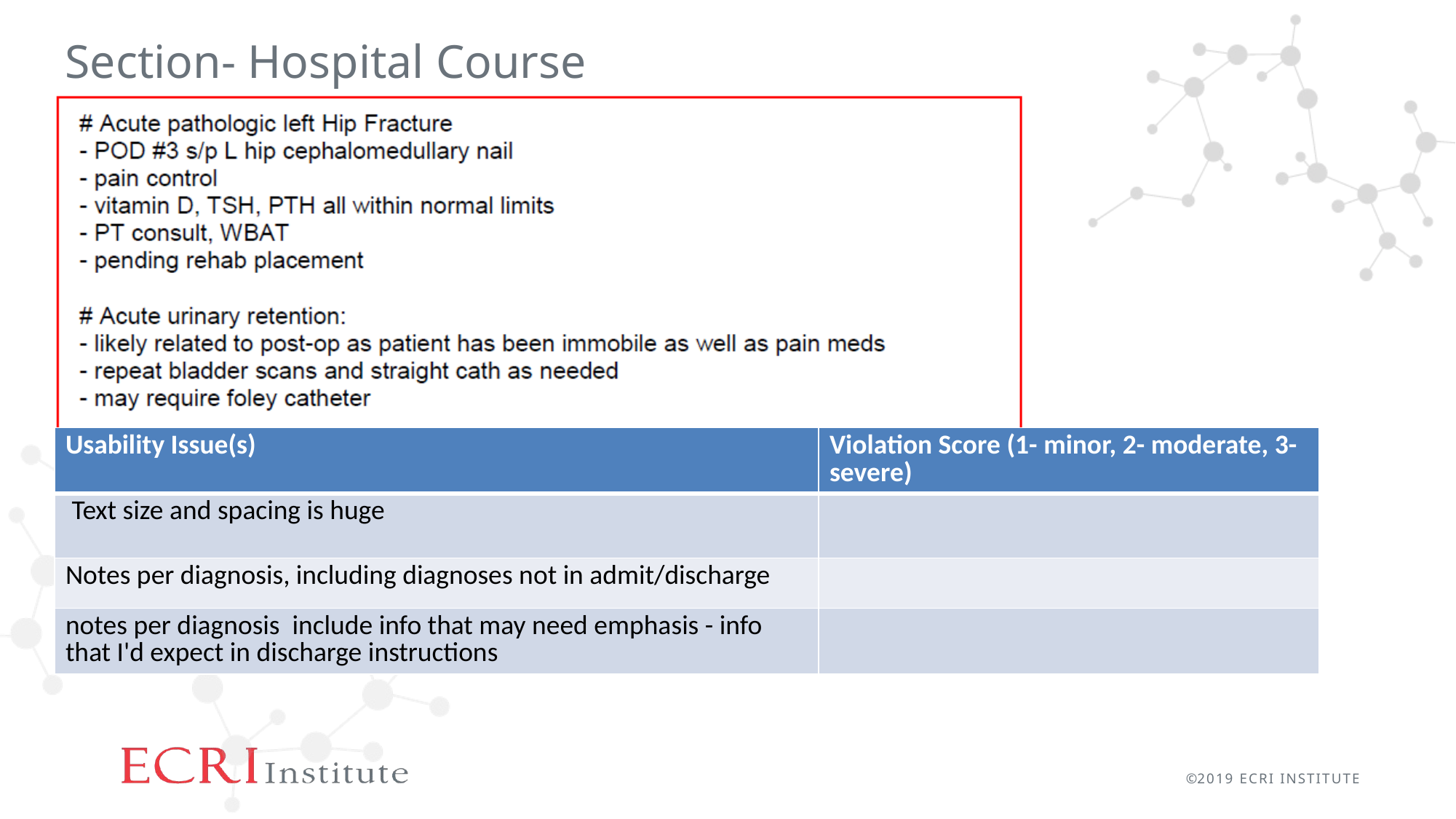

# Section- Hospital Course
| Usability Issue(s) | Violation Score (1- minor, 2- moderate, 3- severe) |
| --- | --- |
| Text size and spacing is huge | |
| Notes per diagnosis, including diagnoses not in admit/discharge | |
| notes per diagnosis include info that may need emphasis - info that I'd expect in discharge instructions | |

## Slide 10
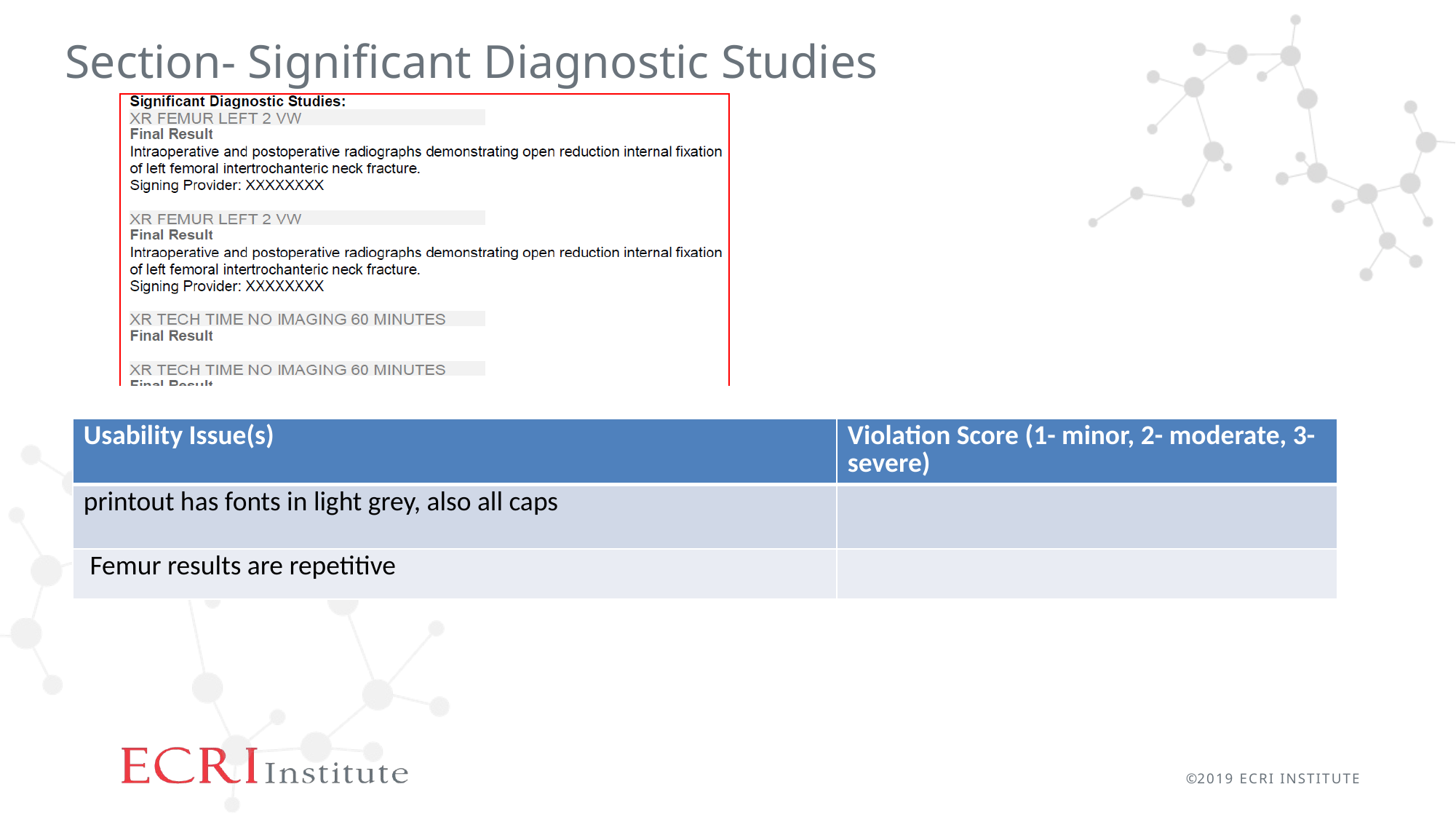

# Section- Significant Diagnostic Studies
| Usability Issue(s) | Violation Score (1- minor, 2- moderate, 3- severe) |
| --- | --- |
| printout has fonts in light grey, also all caps | |
| Femur results are repetitive | |

## Slide 11
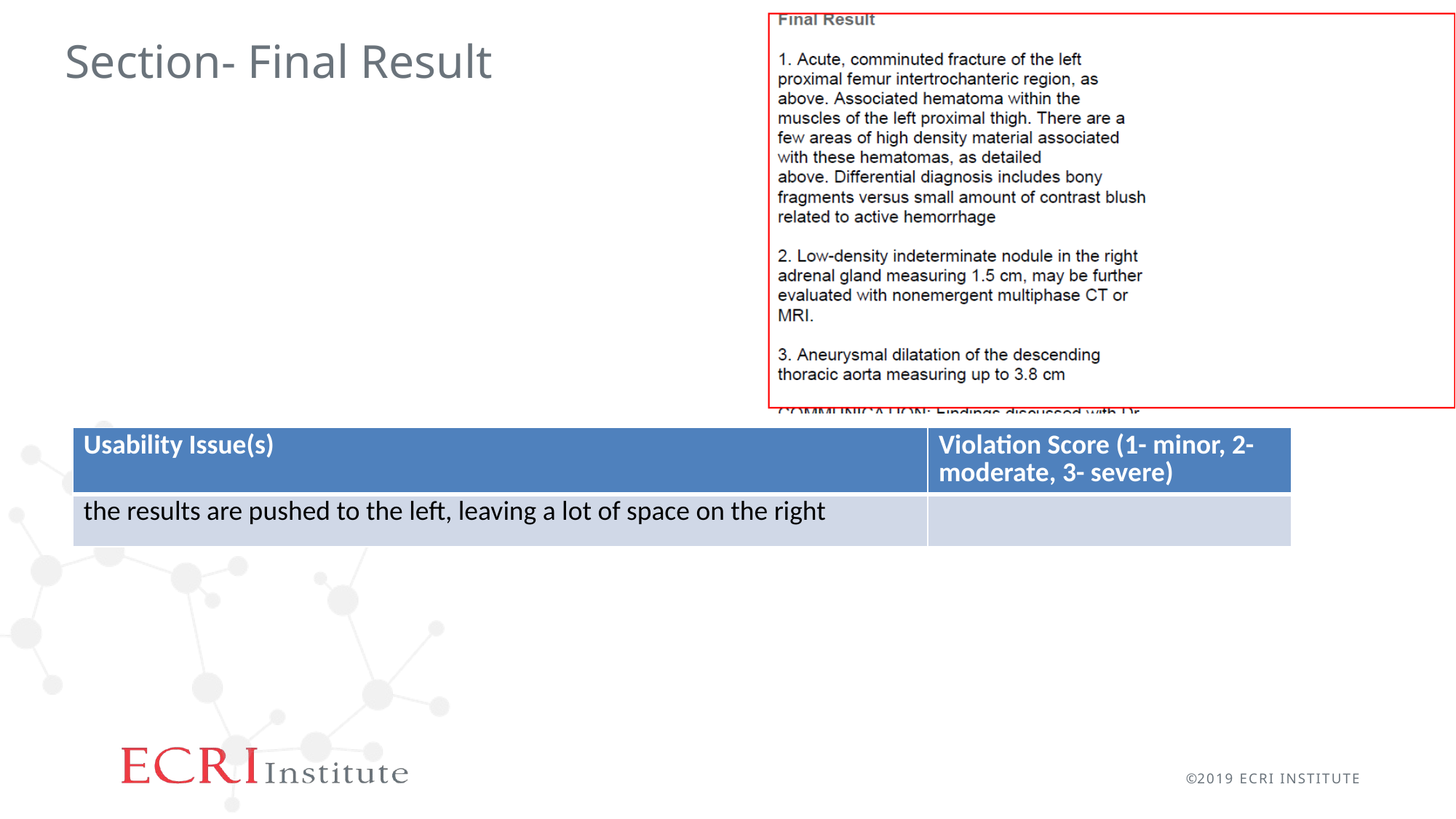

# Section- Final Result
| Usability Issue(s) | Violation Score (1- minor, 2- moderate, 3- severe) |
| --- | --- |
| the results are pushed to the left, leaving a lot of space on the right | |

## Slide 12
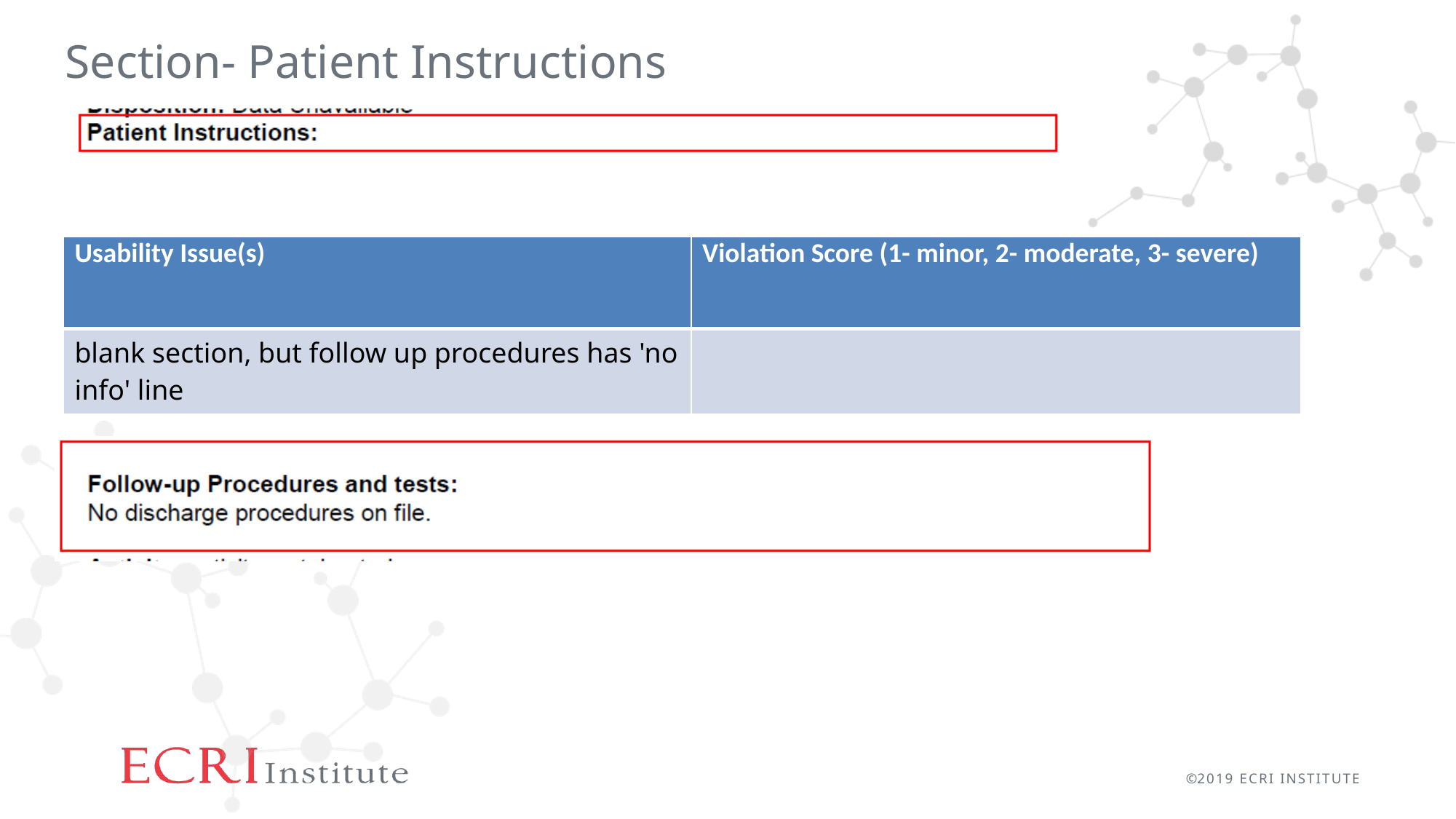

# Section- Patient Instructions
| Usability Issue(s) | Violation Score (1- minor, 2- moderate, 3- severe) |
| --- | --- |
| blank section, but follow up procedures has 'no info' line | |

## Slide 13
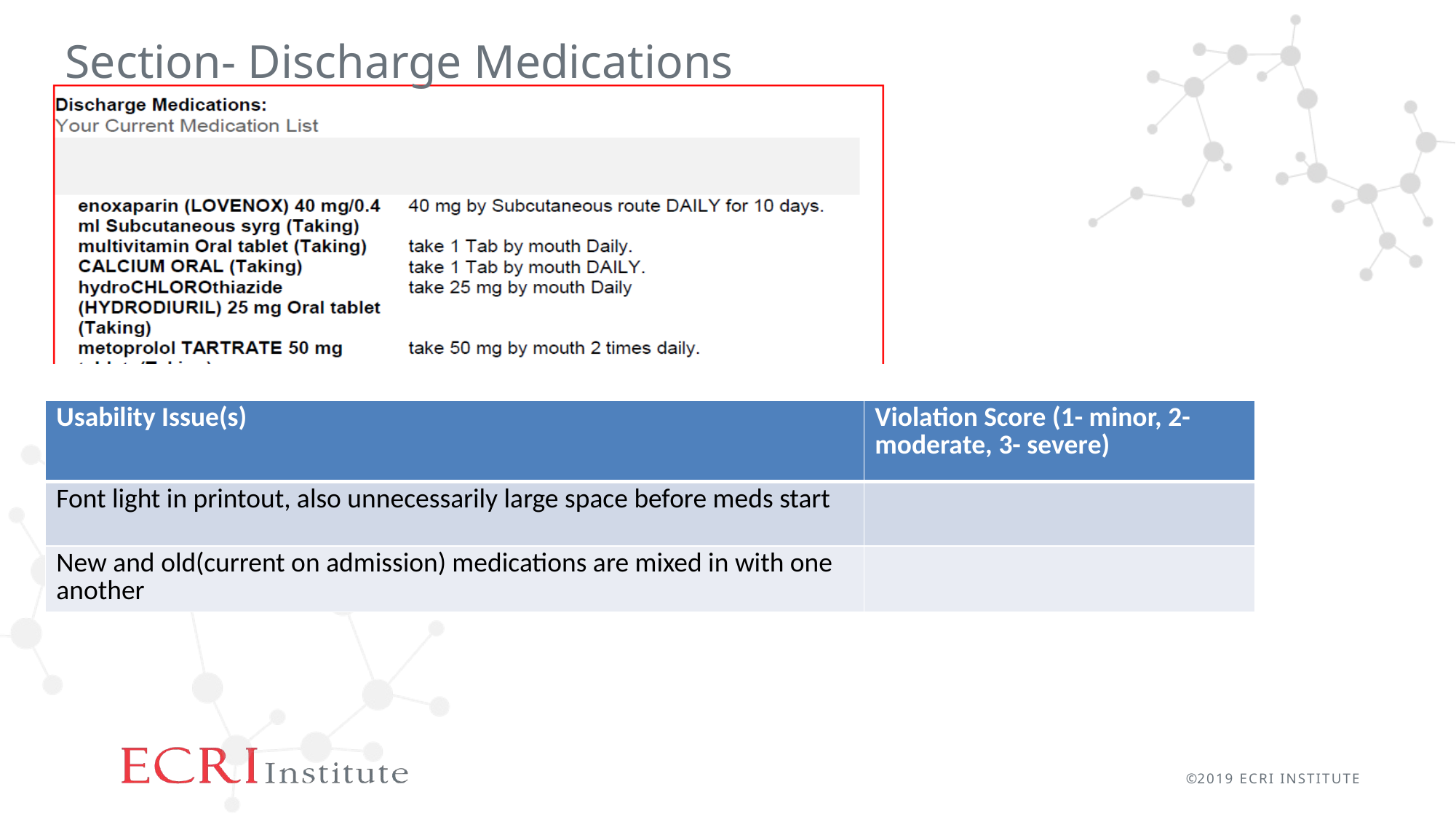

# Section- Discharge Medications
| Usability Issue(s) | Violation Score (1- minor, 2- moderate, 3- severe) |
| --- | --- |
| Font light in printout, also unnecessarily large space before meds start | |
| New and old(current on admission) medications are mixed in with one another | |

## Slide 14
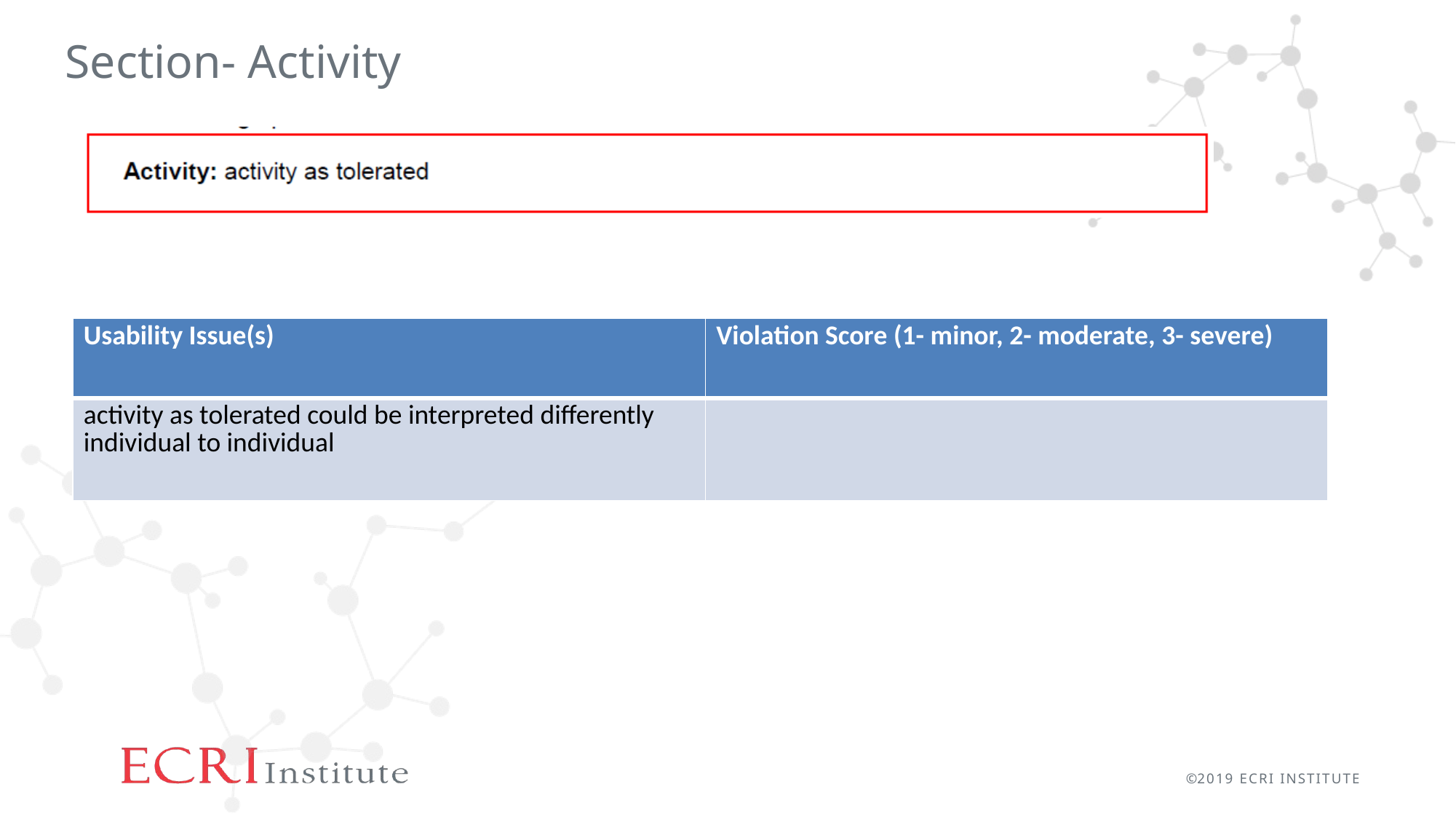

# Section- Activity
| Usability Issue(s) | Violation Score (1- minor, 2- moderate, 3- severe) |
| --- | --- |
| activity as tolerated could be interpreted differently individual to individual | |

## Slide 15
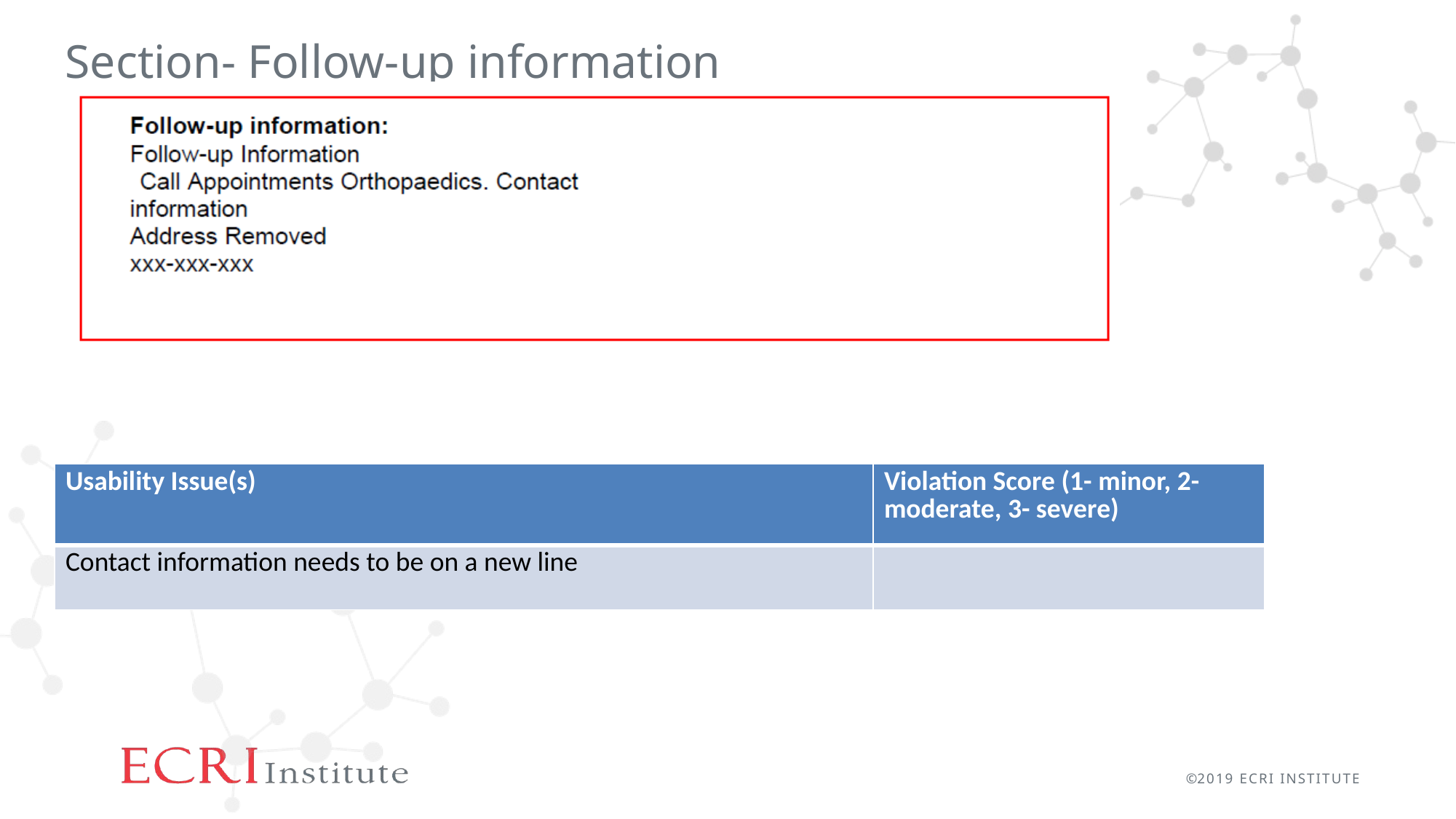

# Section- Follow-up information
| Usability Issue(s) | Violation Score (1- minor, 2- moderate, 3- severe) |
| --- | --- |
| Contact information needs to be on a new line | |

## Slide 16
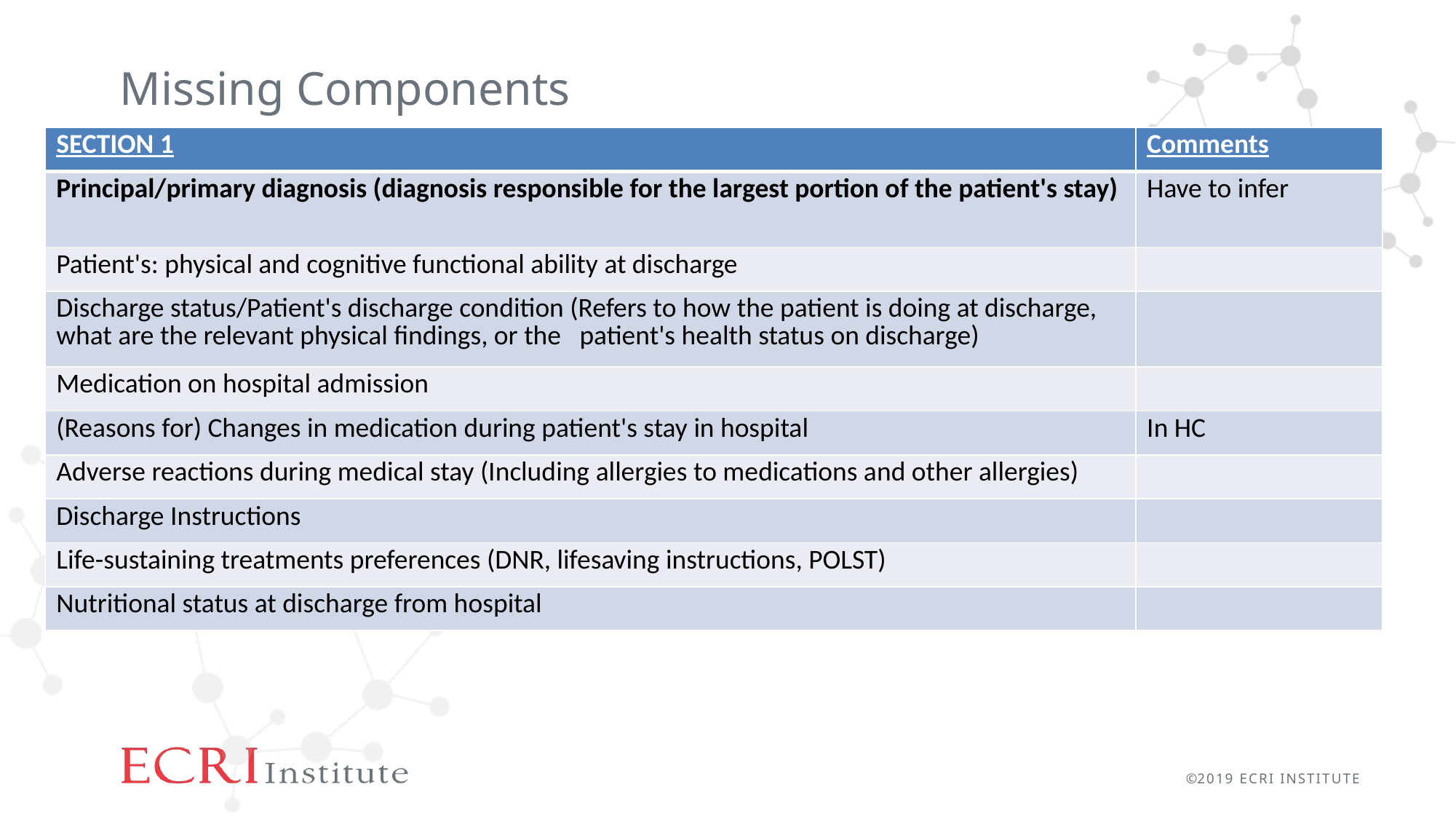

# Missing Components
| SECTION 1 | Comments |
| --- | --- |
| Principal/primary diagnosis (diagnosis responsible for the largest portion of the patient's stay) | Have to infer |
| Patient's: physical and cognitive functional ability at discharge | |
| Discharge status/Patient's discharge condition (Refers to how the patient is doing at discharge, what are the relevant physical findings, or the patient's health status on discharge) | |
| Medication on hospital admission | |
| (Reasons for) Changes in medication during patient's stay in hospital | In HC |
| Adverse reactions during medical stay (Including allergies to medications and other allergies) | |
| Discharge Instructions | |
| Life-sustaining treatments preferences (DNR, lifesaving instructions, POLST) | |
| Nutritional status at discharge from hospital | |

## Slide 17
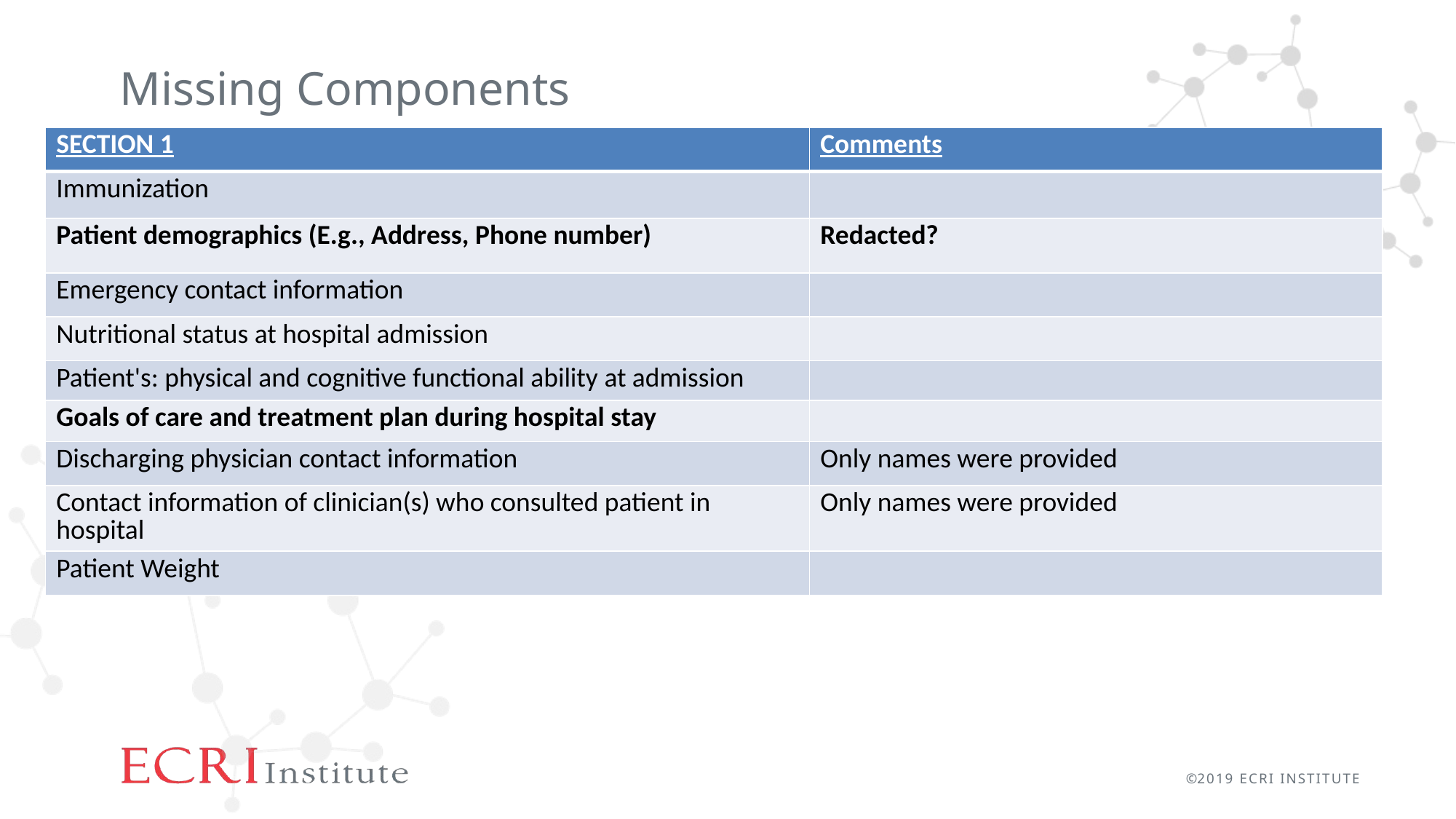

# Missing Components
| SECTION 1 | Comments |
| --- | --- |
| Immunization | |
| Patient demographics (E.g., Address, Phone number) | Redacted? |
| Emergency contact information | |
| Nutritional status at hospital admission | |
| Patient's: physical and cognitive functional ability at admission | |
| Goals of care and treatment plan during hospital stay | |
| Discharging physician contact information | Only names were provided |
| Contact information of clinician(s) who consulted patient in hospital | Only names were provided |
| Patient Weight | |

## Slide 18
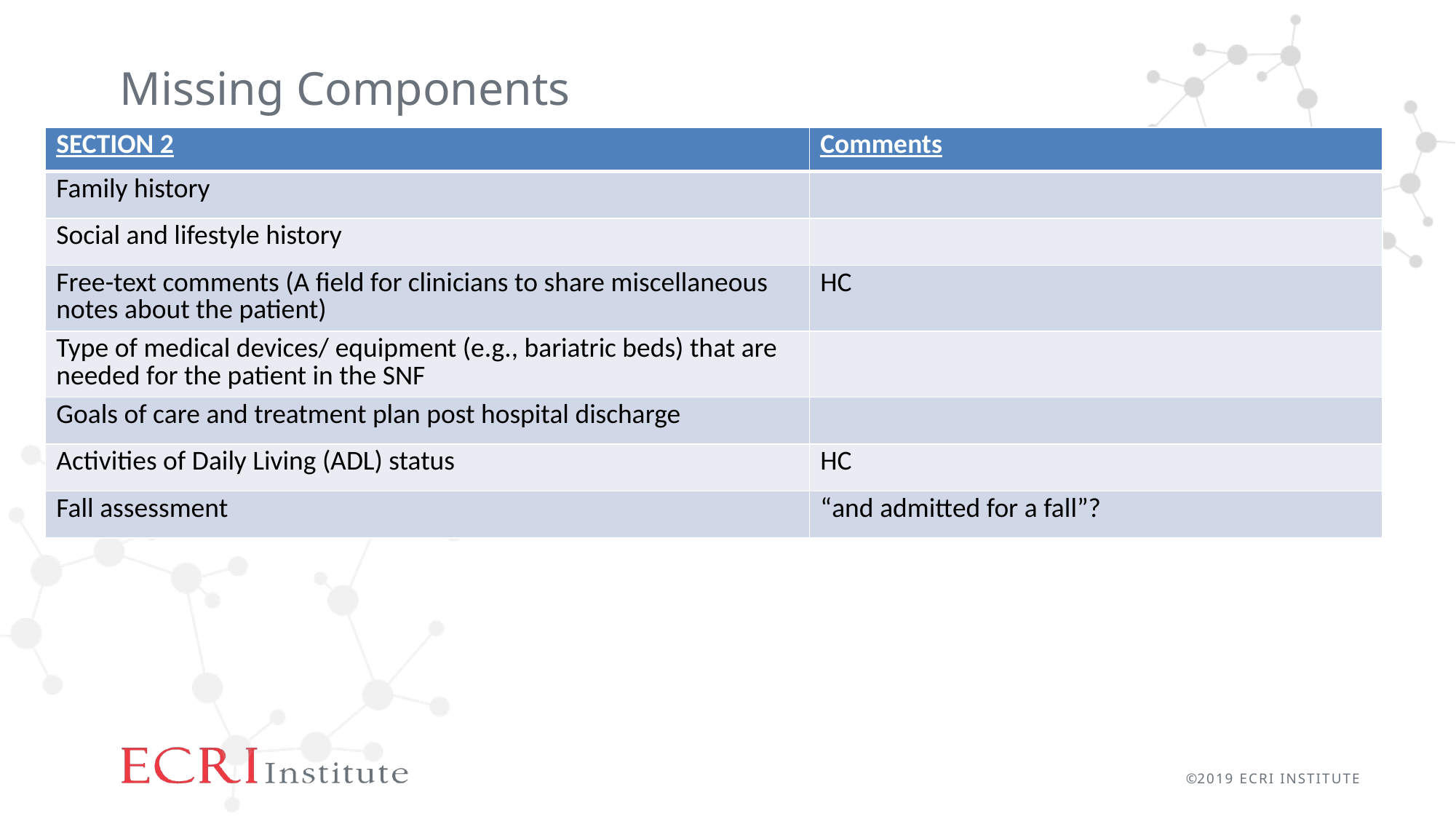

# Missing Components
| SECTION 2 | Comments |
| --- | --- |
| Family history | |
| Social and lifestyle history | |
| Free-text comments (A field for clinicians to share miscellaneous notes about the patient) | HC |
| Type of medical devices/ equipment (e.g., bariatric beds) that are needed for the patient in the SNF | |
| Goals of care and treatment plan post hospital discharge | |
| Activities of Daily Living (ADL) status | HC |
| Fall assessment | “and admitted for a fall”? |

## Slide 19
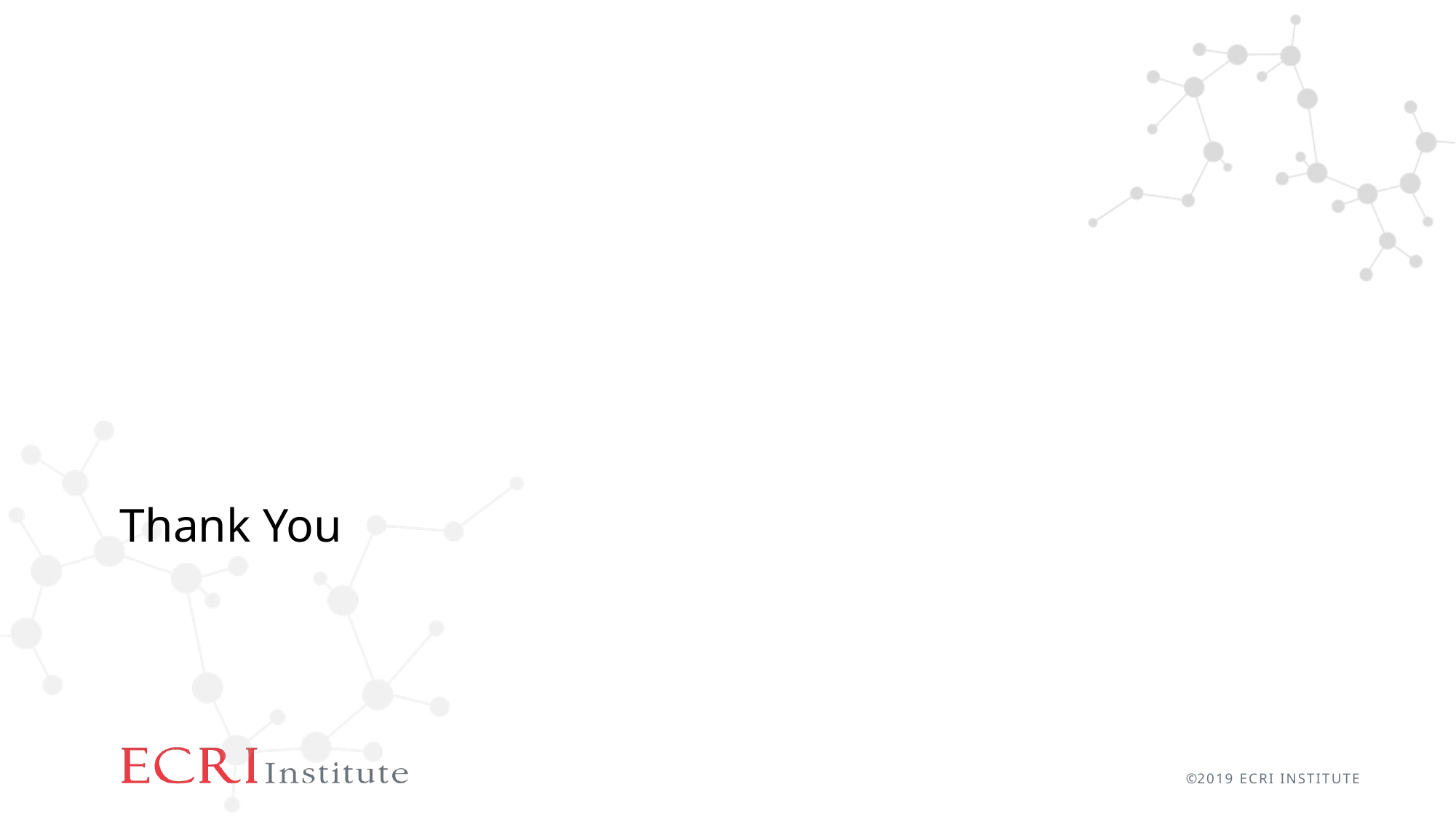

#
