# Supplementary material for: Usability of Electronic Health Record–Generated Discharge Summaries: Heuristic Evaluation
Source: J Med Internet Res. 2021 Apr 15;23(4):e25657. doi: 10.2196/25657 (PMC8085750; doi:10.2196/25657)
Supplement: Multimedia Appendix 13 [file jmir_v23i4e25657_app13.docx]

Appendix 13

Table A13. Locations of several DS issues identified by clinical experts and the recommendations that are related to those issues. (Descriptions of the potential Issues identified by human factors team are shown in Appendixes 6-9.)

| **Discharge Summary ID and section** | **Issue Identified by Clinical Expert(s)** | **Recommendation(s)** |
| --- | --- | --- |
| H1P1 hospital course | Feeding tube-This looks like a complex patient being intubated (feeding tube). The feeding tube is only mentioned in the HC, it is not mentioned anywhere else, so the SNF would have to be call the hospital and ask them about the feeding tube and dietary restrictions | Ensure that *all* procedures undergone during acute visit are listed; Examples include: the use of feeding tubes, dietary restrictions, or TPN, urinary catheter. |
| H1P1 Physical exam | Height, weight not mentioned | New guideline #1 (Table 2) “patient weight” |
| H1P1 Medication list | 1. There is no diagnosis with corresponding medications 2. There is no listing between new starts, discontinuation 3. Insulin- 0-14 units, what is the adjustment rate? There needs to be specific instructions and adjustment rates | Require users to make sure that medication information is complete. |
| H1P2 Discharge Diagnoses | For Hepatitis C, this is treatable. So they need to know some additional discharge status and what the steps would be. Additional information is needed. | New guideline # 2 (Table 3) “Goals of care and treatment plan post discharge” |
| H1P2 Medications | 1. Need information on the PRN. PRN is as needed. So need to know the as needed reason 2. Perdisone- is an incomplete order. Frequency is not listed | Require users to make sure that medication information is complete. |
| H1P2 Discharge medication | D/C medication changes should be in the D/C meds section. Meds dictates what the orders going to be. | Require users to make sure that medication information is complete. |
| H2P1 Current medications list | 1. Medications needs to be prioritized. The most important medication (warfarin) is buried. 2. Estrogen is not even on a separate line. 3. Usually “taking” was indicated, but then on certain medications like aspirin, it is not mentioned at all, creating confusion | Require users to make sure that medication information is complete. |
| H2P1 Follow-up information | Post up patient with no wound care instructions | New guideline # 2 (Table 3) “Goals of care and treatment plan post discharge” |
| H2P2 Final Result | Labelling 'final result' is confusing since this is specific to the CT abdomen results | Ensure that the content matches the headings and subheadings within each section**.** |
| H2P2 Discharge diagnosis | "Active hospital" and "Diagnosis'- you don’t need two separate lines. Just one line is sufficient | Apply consistent font style, font sizing, spacing, layout, indentation, and heading style. |
